# Supplementary material for: Let‐7a‐regulated translational readthrough of mammalian AGO1 generates a microRNA pathway inhibitor
Source: EMBO J. 2019 Jul 22;38(16):e100727. doi: 10.15252/embj.2018100727 (PMC6694283; doi:10.15252/embj.2018100727)
Supplement: Supplementary file 1 — Appendix [file EMBJ-38-e100727-s001.pdf]

Appendix

Table of contents

1. Appendix Figure S1 to S5
2. Legends to Appendix Supplementary Figures
3. Appendix Table S1 to S3

Appendix Figure S1

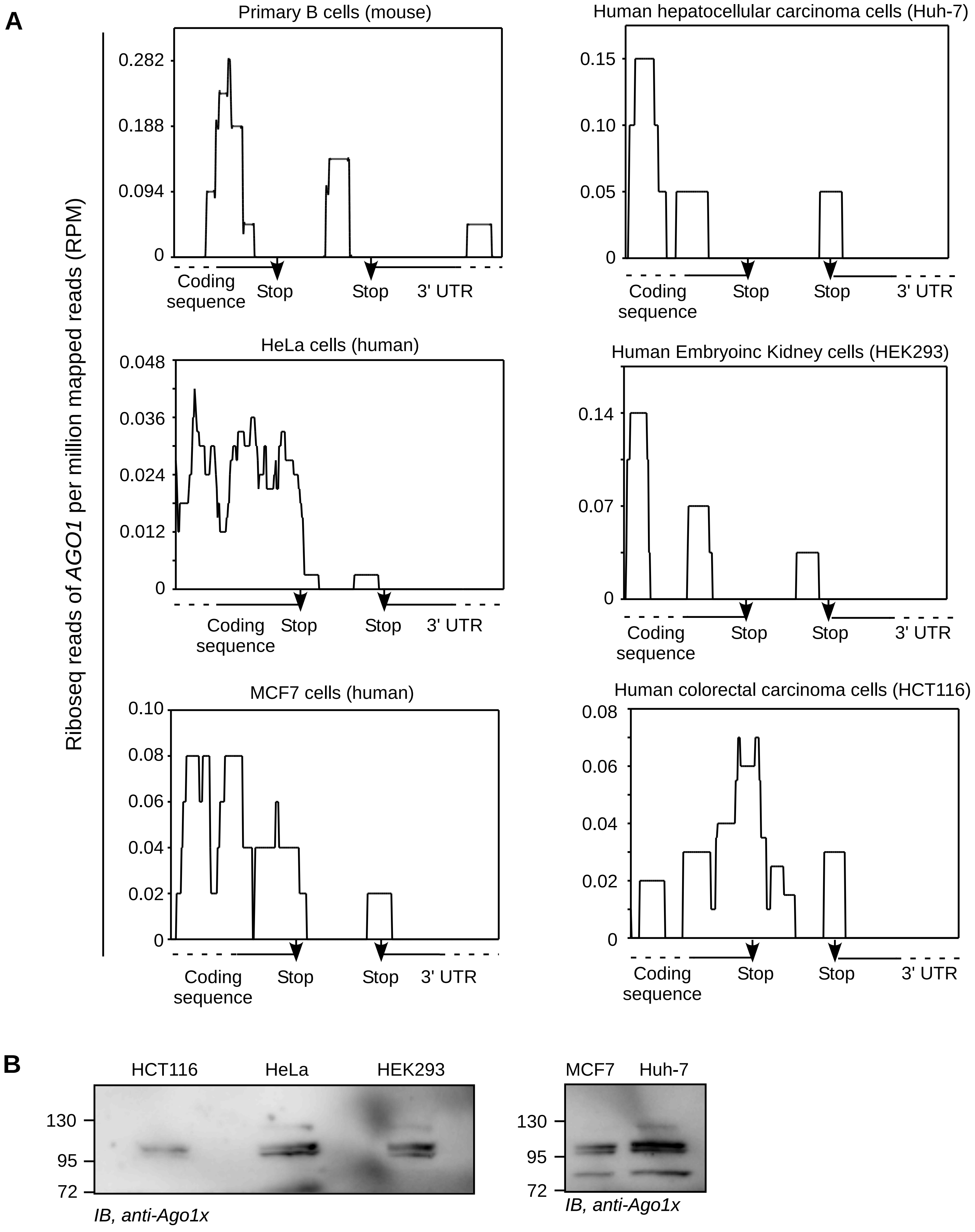

Appendix Figure S2

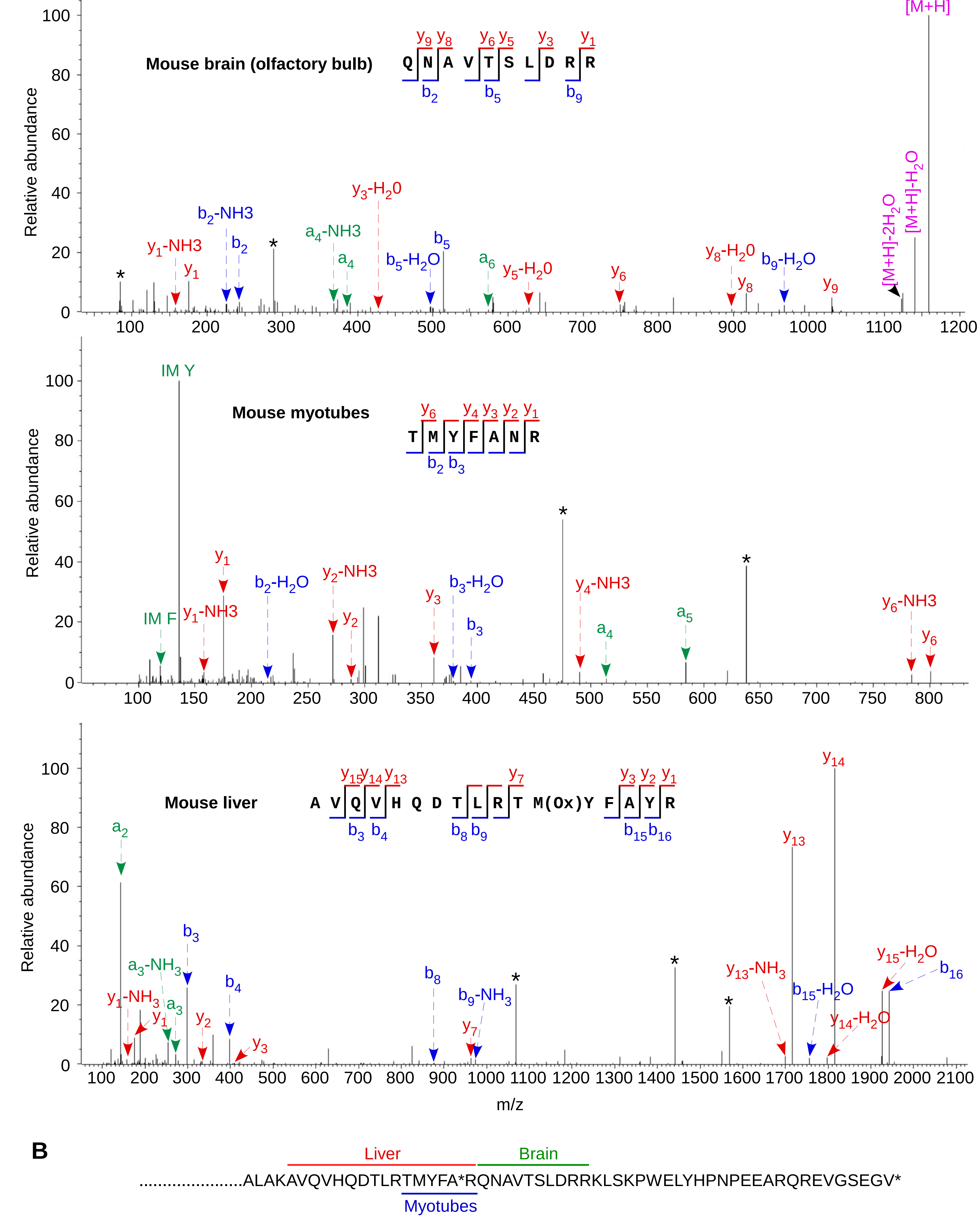

Appendix Figure S3

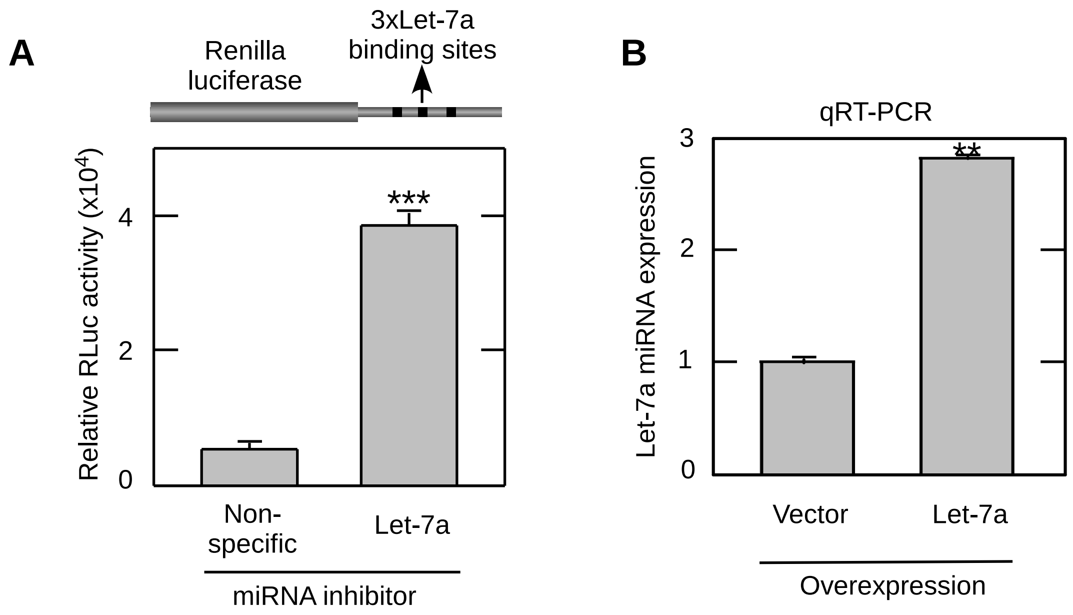

Appendix Figure S4

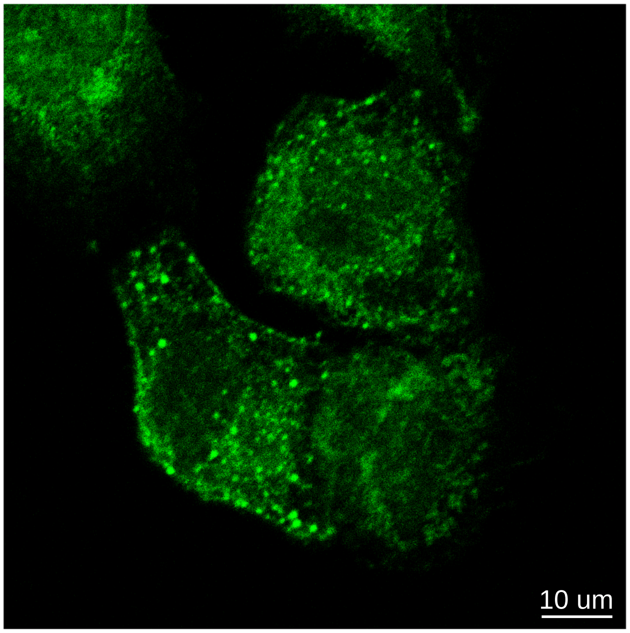

IF, anti-Ago1x

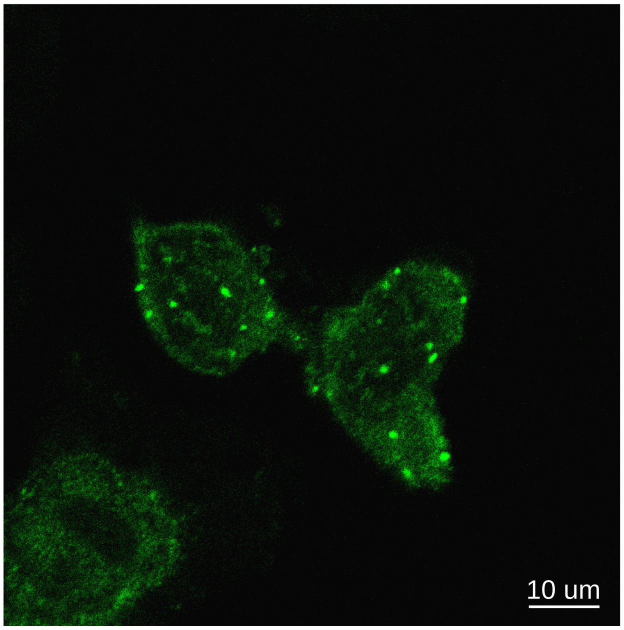

IF, anti-Ago1

Appendix Figure S5

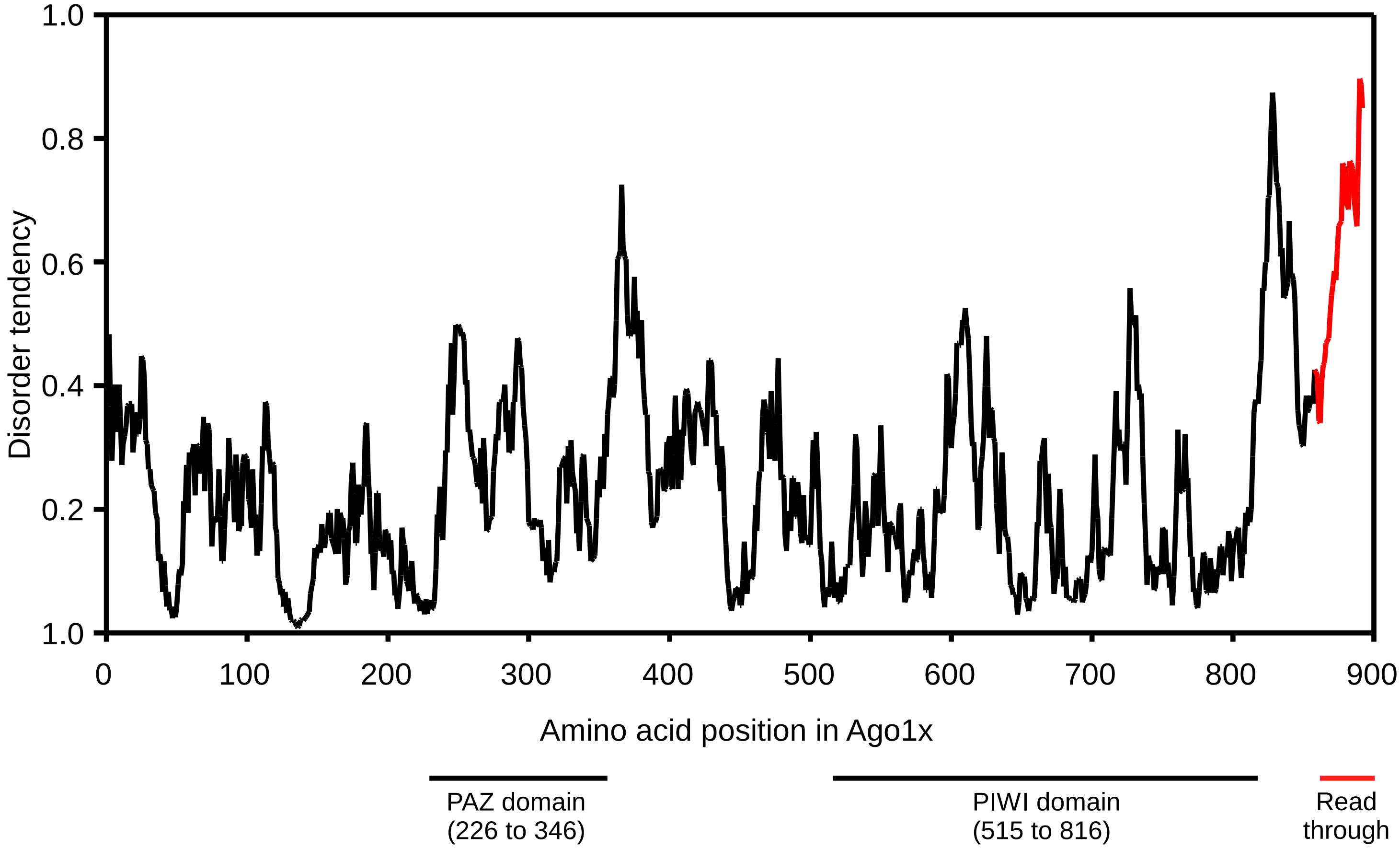

Legends to Appendix Supplementary Figures

Appendix Figure S1. (A) Presence of ribosome footprints in the Inter stop codon region of *AGO1* transcript in mouse primary B cells (NCBI Accession no. SRR1605309), Huh-7 cells (NCBI Accession no. SRR2052945), HeLa cells (NCBI Accession no. SRR5013257), HEK293 cells (NCBI Accession no. SRR2096965), MCF7 cells (NCBI Accession no. SRR5345623) and HCT116 cells (NCBI Accession no. SRR4293695). Ribosome profiling data was taken from NCBI's sequence read archive (SRA) and ribosome footprints on *AGO1* transcript were analyzed as described in Methods section. Arrows indicate the position of canonical stop codon and the downstream in-frame stop codon. (B) Expression of Ago1x in multiple cell lines.

Appendix Figure S2. (A) MS/MS spectra of Ago1x-specific peptides found in mouse brain (olfactory bulb), liver and myotubes. The spectra contain several contaminant ions (*) probably because of low abundance. (B) The position of these peptides in the C-terminus of Ago1x is shown. *, position of two stop codons.

Appendix Figure S3. (A) Validation of Let-7a miRNA inhibitor. RLuc with 3x Let-7a miRNA binding sites in its 3′UTR was expressed in HeLa cells treated with Let-7a miRNA inhibitor or control inhibitor. RLuc activity along with the activity of co-transfected FLuc was measured after 48 h. ***, $P < 0.0001$ (Student's t test). (B) Demonstration of overexpression of Let-7a miRNA in HeLa cells stably expressing Pri-Let-7a. Quantitative real time PCR was done using Let-7a-specific Taqman probe. **, $P = 0.0015$ (Student's t test with Welch's correction).

Appendix Figure S4. Confocal immunofluorescence images of HeLa cells showing punctate distribution of Ago1 and Ago1x.

Appendix Figure S5. Estimation of disorder tendency at every amino acid position in Ago1x. The analysis was done using IUPred web server.

Appendix Table S1. List of miRNAs interacting with both Ago1 and Ago1x

	miRDeep2 ID	miRBase ID	Mean RPM in Ago1 IP samples (N=2)	Mean RPM in Ago1x IP samples (N=2)
1	chr10_515951	NA	12926.475	6673.24
2	chr10_515952	NA	8157.835	3825.53
3	chr10_520588	NA	458.505	251.835
4	chr10_521097	NA	631.31	308.105
5	chr10_533286	hsa-miR-146b-5p	122.12	222.33
6	chr10_537166	NA	384.525	222.33
7	chr10_547194	NA	210.435	500.92
8	chr10_550195	NA	1803.06	752.755
9	chr10_553552	hsa-miR-107	309.265	637.47
10	chr10_555599	NA	1406.75	1502.76
11	chr10_560303	NA	125.95	388.385
12	chr10_561004	NA	190.985	530.425
13	chr1_100648	NA	1027.615	1001.84
14	chr1_1068	NA	705.295	559.94
15	chr1_10724	hsa-miR-30e-5p	663.83	723.25
16	chr1_1291	NA	1757.755	4045.11
17	chr11_562701	NA	1239.33	835.785
18	chr11_562755	NA	2243.385	2456.575
19	chr11_562912	NA	336.39	334.865
20	chr11_563574	NA	311.82	640.22
21	chr11_567981	NA	649.485	139.3
22	chr11_570824	NA	471.565	308.105
23	chr11_577072	NA	87.04	278.595
24	chr11_577740	NA	6773.45	5443.58
25	chr11_580089	NA	406.535	334.865
26	chr1_15855	NA	954.91	2818.195
27	chr11_585557	NA	1641.76	2785.945
28	chr11_587294	NA	7175.14	4296.945
29	chr11_593469	NA	1045.79	1398.47
30	chr11_606128	NA	6778.565	5582.875
31	chr11_610495	NA	3442.26	3908.56
32	chr11_610655	hsa-miR-125b-5p	141.57	278.595
33	chr11_610667	hsa-let-7a-5p	627.48	699.23
34	chr11_610685	hsa-miR-100-5p	2285.125	1146.635
35	chr11_611704	NA	3565.925	4723.08
36	chr1_18163	NA	296.2	693.735
37	chr1_22683	NA	584.46	785.01

38	chr12_621477	NA	16721.01	23312.79
39	chr12_625662	hsa-miR-196a-5p	507.915	583.95
40	chr12_625839	hsa-miR-148b-3p	1919.05	1256.42
41	chr12_627852	hsa-let-7i-5p	18726.83	24982.22
42	chr12_634411	NA	209.16	305.355
43	chr12_634492	NA	1526.31	1896.64
44	chr12_638876	NA	574.225	752.755
45	chr12_644268	NA	367.625	278.595
46	chr12_648707	NA	211.715	308.105
47	chr12_652098	NA	897.555	589.445
48	chr12_653472	NA	6833.37	4270.185
49	chr12_653813	hsa-miR-26a-5p	5714.6	6635.49
50	chr12_659503	NA	419.595	251.835
51	chr12_660371	NA	352.01	500.92
52	chr12_660934	NA	622.085	613.455
53	chr12_665621	NA	105.22	471.415
54	chr12_665637	NA	248.07	195.565
55	chr12_666349	NA	229.89	139.3
56	chr12_666626	NA	740.365	814.52
57	chr1_32007	hsa-miR-92b-3p	674.33	918.81
58	chr1_32671	NA	175.365	361.62
59	chr1_32709	NA	658.44	278.595
60	chr13_667956	NA	742.925	616.205
61	chr13_672506	NA	794.895	975.08
62	chr13_674508	hsa-miR-17-5p	1098.035	557.19
63	chr13_674514	hsa-miR-20a-5p	973.36	723.25
64	chr13_674517	hsa-miR-92a-3p	2224.2	2936.23
65	chr13_681308	NA	1767.715	2097.7
66	chr13_683411	NA	1631.53	1283.18
67	chr13_687732	NA	519.7	669.725
68	chr14_689848	NA	205.32	471.415
69	chr14_695564	NA	350.73	696.485
70	chr14_696052	NA	337.665	640.22
71	chr14_696883	NA	53.245	139.3
72	chr14_696884	NA	53.245	139.3
73	chr14_700198	NA	5796.795	6262.88
74	chr14_703755	NA	267.525	195.565
75	chr14_704155	NA	205.32	471.415
76	chr14_710542	NA	348.17	139.3
77	chr14_710656	NA	193.54	672.475
78	chr14_715404	NA	420.875	388.385

79	chr14_716600	NA	1168.915	1446.49
80	chr14_716649	NA	1319.98	2611.645
81	chr1_4901	NA	7037.135	5695.415
82	chr1_4902	NA	6876.115	5556.115
83	chr1_49605	NA	5992.54	2245.235
84	chr1_51950	NA	212.995	833.035
85	chr1_54130	NA	329.995	139.3
86	chr1_54547	NA	229.89	417.89
87	chr1_5456	NA	1951.02	3075.525
88	chr1_557	NA	137.73	334.865
89	chr15_727648	NA	211.715	222.33
90	chr15_728069	NA	141.57	222.33
91	chr15_728070	NA	159.745	278.595
92	chr15_728403	NA	3051.34	3308.135
93	chr15_728404	NA	3122.77	3308.135
94	chr15_730308	hsa-miR-7-5p	8055.99	5341.32
95	chr15_734615	NA	5153.43	4693.575
96	chr15_734847	NA	2131.775	2643.9
97	chr15_737274	NA	771.605	471.415
98	chr15_743512	NA	922.4	752.755
99	chr15_744400	NA	279.305	278.595
100	chr15_746053	NA	8311.545	5076.46
101	chr1_59006	NA	1028.625	251.835
102	chr1_61018	NA	6789.065	5526.61
103	chr1_63044	NA	441.61	586.695
104	chr1_65493	NA	106.5	251.835
105	chr1_67213	NA	368.905	251.835
106	chr16_751892	NA	87.04	417.89
107	chr16_754052	NA	821.02	1229.665
108	chr16_755309	hsa-miR-193b-3p	439.05	251.835
109	chr16_755329	hsa-miR-365b-3p	103.94	222.33
110	chr16_756237	NA	8184.855	13415.77
111	chr16_759433	NA	5994.445	8004.45
112	chr16_766319	hsa-miR-140-3p	229.89	388.385
113	chr16_769655	NA	314.375	782.26
114	chr16_772866	NA	209.16	388.385
115	chr16_774510	NA	271.355	195.565
116	chr16_774999	NA	967.7	980.575
117	chr16_775717	NA	5474.935	8246.005
118	chr16_775718	NA	5511.285	8133.465
119	chr16_776552	NA	8184.855	13415.77

120	chr16_776886	NA	8184.855	13415.77
121	chr16_777593	NA	3065.135	4021.1
122	chr16_778903	NA	105.22	361.62
123	chr16_781331	NA	35.07	222.33
124	chr16_782558	NA	262.405	278.595
125	chr16_789457	NA	6643.66	4130.89
126	chr16_789458	NA	6624.21	4047.86
127	chr16_790032	NA	316.935	278.595
128	chr1_69800	hsa-miR-101-3p	883.49	476.91
129	chr1_71005	hsa-miR-186-5p	2341.205	1591.285
130	chr1_71602	NA	6917.58	5443.58
131	chr1_73352	NA	566.28	894.8
132	chr1_75296	NA	106.5	251.835
133	chr1_76099	NA	559.89	1108.88
134	chr1_77720	NA	2322.755	865.29
135	chr17_793251	NA	153.35	139.3
136	chr17_793252	NA	293.645	391.135
137	chr17_793285	NA	1314.59	924.305
138	chr17_801209	hsa-miR-423-3p	976.925	2236.995
139	chr17_801574	NA	190.985	892.055
140	chr17_802143	hsa-miR-365b-3p	205.32	222.33
141	chr17_802354	NA	190.985	892.055
142	chr17_808740	NA	6773.45	5443.58
143	chr17_808903	NA	584.46	222.33
144	chr17_810968	hsa-miR-21-5p	30877.645	15816.08
145	chr17_814203	NA	474.12	474.16
146	chr17_815626	NA	4960.08	6273.87
147	chr17_822778	hsa-miR-324-3p	139.015	334.865
148	chr17_827132	NA	248.07	222.33
149	chr17_828328	NA	172.81	308.105
150	chr17_830622	NA	192.26	278.595
151	chr17_830646	NA	122.12	251.835
152	chr17_830670	NA	1912.385	1567.27
153	chr17_832661	NA	7276.25	4465.75
154	chr17_833160	NA	87.04	444.65
155	chr17_834991	hsa-miR-10a-5p	279.305	139.3
156	chr17_835033	hsa-miR-196a-5p	457.225	583.95
157	chr17_835105	NA	12119.14	17919.27
158	chr17_842466	NA	336.39	530.425
159	chr17_843305	NA	4340.36	3158.555
160	chr17_844209	NA	676.885	1371.705

161	chr1_8291	NA	261.13	278.595
162	chr1_8292	NA	345.615	278.595
163	chr1_84457	NA	450.835	1146.635
164	chr18_849269	NA	140.295	222.33
165	chr18_854970	NA	8202.215	7833.605
166	chr18_864531	NA	539.155	696.485
167	chr1_91983	NA	298.76	308.105
168	chr1_92569	hsa-miR-181a-5p	348.17	616.205
169	chr1_92699	NA	10901.66	14347.61
170	chr1_93620	NA	10466.96	20763.52
171	chr1_95190	hsa-miR-29b-3p	172.81	388.385
172	chr19_864676	NA	6987.725	5443.58
173	chr19_866213	NA	212.995	471.415
174	chr19_867550	hsa-miR-7-5p	8022.195	5341.32
175	chr19_870255	NA	346.89	474.16
176	chr19_871200	hsa-miR-181d-5p	592.4	417.89
177	chr19_871999	NA	3903.05	9459.18
178	chr19_879863	NA	348.17	444.65
179	chr19_880540	hsa-miR-769-5p	1231.66	308.105
180	chr19_882832	hsa-miR-99b-5p	1005.6	1736.075
181	chr19_882834	hsa-let-7e-5p	70.145	583.95
182	chr19_884542	NA	1570.61	222.33
183	chr19_885366	NA	209.16	391.135
184	chr19_893131	hsa-miR-24-3p	1333.04	1028.6
185	chr19_893132	hsa-miR-24-3p	1333.04	1028.6
186	chr19_893134	hsa-miR-27a-3p	2120.54	2172.485
187	chr19_893136	hsa-miR-23a-3p	1127.72	1505.51
188	chr19_894202	NA	280.58	139.3
189	chr19_896093	NA	71.425	195.565
190	chr19_899247	NA	428.545	195.565
191	chr19_900532	NA	559.89	554.445
192	chr19_902305	NA	805.4	2555.38
193	chr19_903014	NA	747.035	1333.955
194	chr1_99994	NA	9751.275	12904.575
195	chr20_903705	NA	9119.605	2269.25
196	chr20_904393	NA	1552.16	1564.525
197	chr20_904665	hsa-miR-103a-3p	1004.325	1221.42
198	chr20_905315	NA	623.365	533.175
199	chr20_906955	NA	6917.58	5443.58
200	chr20_908602	NA	2242.11	2116.215
201	chr20_910048	NA	24590.81	30934.345

202	chr20_910354	NA	106.5	476.91
203	chr20_911998	NA	437.77	305.355
204	chr20_916773	NA	2251.335	3150.31
205	chr20_919086	NA	1063.7	699.23
206	chr20_922372	NA	1143.34	2697.42
207	chr20_925845	NA	4207.74	4195.4
208	chr2_104262	NA	1143.34	1976.925
209	chr2_112290	NA	2291.52	1819.1
210	chr2_115447	NA	348.17	195.565
211	chr2_116746	NA	6551.505	4406.735
212	chr2_118220	NA	2845.75	2011.925
213	chr2_118617	NA	1192.205	921.56
214	chr2_123783	NA	1786.43	3458.415
215	chr2_124989	hsa-miR-128-3p	120.835	139.3
216	chr2_127594	NA	1433.88	1259.17
217	chr2_128179	NA	2842.73	2628.125
218	chr2_129488	hsa-miR-7704	423.43	557.19
219	chr2_135714	NA	35.07	278.595
220	chr2_144039	NA	1335.6	1390.225
221	chr2_148478	NA	3564.645	2818.195
222	chr2_149753	NA	123.395	305.355
223	chr2_156104	NA	192.26	251.835
224	chr2_157775	NA	3904.875	2676.155
225	chr2_160605	NA	886.045	977.83
226	chr2_170075	NA	1494.8	809.025
227	chr2_170532	NA	682.005	1861.635
228	chr21_929294	hsa-miR-99a-5p	2616.675	1985.16
229	chr21_929296	hsa-let-7c-5p	684.56	696.485
230	chr21_930333	NA	2300.2	2759.185
231	chr21_932092	NA	720.915	586.695
232	chr21_934676	NA	2368.06	2601.37
233	chr21_934983	NA	2009.385	2121.71
234	chr21_935968	NA	387.08	139.3
235	chr21_936000	NA	3440.985	3544.19
236	chr21_939166	NA	2183.745	3916.8
237	chr22_942024	NA	205.32	471.415
238	chr22_943965	NA	7464.87	12377.605
239	chr22_948026	NA	5830.51	2676.155
240	chr22_950730	NA	675.61	251.835
241	chr22_951595	NA	479.24	278.595
242	chr22_951716	hsa-let-7a-5p	627.48	699.23

243	chr22_951933	NA	187.145	500.92
244	chr22_952191	NA	4028.815	4969.42
245	chr22_955865	NA	923.68	1084.87
246	chr22_956078	NA	702.74	308.105
247	chr22_956080	NA	1279.25	755.5
248	chr22_959748	NA	1719.85	2006.43
249	chr22_963646	NA	453.39	779.515
250	Chr22_963646.1	NA	350.73	779.515
251	chr3_177407	NA	157.19	83.03
252	chr3_178180	NA	6643.66	4130.89
253	chr3_180564	hsa-miR-26a-5p	5623.72	6635.49
254	chr3_185468	NA	739.09	447.4
255	chr3_197690	NA	229.89	471.415
256	chr3_203486	NA	50313.72	52895.275
257	chr3_209684	NA	244.23	334.865
258	chr3_212445	hsa-miR-191-5p	674.33	833.035
259	chr3_213633	NA	215.555	391.135
260	chr3_213832	NA	7925.385	11567.165
261	chr3_213840	NA	7925.385	11567.165
262	chr3_214157	hsa-let-7g-5p	3328.36	3479.68
263	chr3_233160	NA	161.025	195.565
264	chr4_234450	NA	601.355	889.305
265	chr4_235708	NA	6689.51	8173.255
266	chr4_235908	NA	1339.435	444.65
267	chr4_236851	NA	3120.21	2673.41
268	chr4_237130	NA	1231.39	2228.755
269	chr4_240973	NA	609.3	305.355
270	chr4_249460	NA	910.615	476.91
271	chr4_250922	NA	305.15	195.565
272	chr4_251730	NA	910.615	1639.315
273	chr4_254139	NA	2298.185	2389.32
274	chr4_255580	hsa-miR-378d	176.645	139.3
275	chr4_259375	NA	335.11	364.37
276	chr4_262115	NA	196.095	361.62
277	chr4_267902	NA	284.42	417.89
278	chr5_276250	NA	1562.66	1066.355
279	chr5_283090	NA	477.96	388.385
280	chr5_292783	NA	757.265	613.455
281	chr5_295371	NA	261.13	278.595
282	chr5_295894	NA	159.745	251.835
283	chr5_296799	hsa-miR-378a-3p	2203.2	2261.01

284	chr5_300725	NA	466.18	251.835
285	chr5_315244	NA	791.06	833.035
286	chr5_319207	NA	177.92	251.835
287	chr5_323590	NA	387.08	420.64
288	chr5_325879	NA	397.58	557.19
289	chr5_327216	hsa-miR-103a-3p	1004.325	1221.42
290	chr5_329681	hsa-miR-340-5p	231.175	278.595
291	chr6_340304	NA	266.245	334.865
292	chr6_340851	NA	337.665	361.62
293	chr6_344036	NA	811.795	1034.095
294	chr6_345321	NA	1484.845	918.81
295	chr6_350173	NA	6968.27	5443.58
296	chr6_354527	NA	261.13	361.62
297	chr6_364859	hsa-miR-30c-5p	640.535	1371.705
298	chr6_364939	hsa-miR-30a-5p	12494.44	11479.35
299	chr6_369309	NA	505.36	720.5
300	chr6_369976	NA	363.79	616.205
301	chr6_375592	NA	2299.195	3016.51
302	chr6_376857	NA	4709.725	5355.055
303	chr7_386204	NA	354.565	554.445
304	chr7_393364	NA	565	583.95
305	chr7_393536	NA	946.97	980.575
306	chr7_394572	NA	1031.18	500.92
307	chr7_399287	NA	338.945	139.3
308	chr7_400245	NA	105.22	444.65
309	chr7_404189	NA	226.055	251.835
310	chr7_405477	NA	2153.785	3581.23
311	chr7_405642	NA	997.66	1143.885
312	chr7_409612	NA	811.795	1004.59
313	chr7_409849	hsa-miR-148a-3p	1409.585	1058.11
314	chr7_409850	hsa-miR-148a-3p	1409.585	1058.11
315	chr7_409950	NA	107.775	139.3
316	chr7_413092	NA	1034.01	586.695
317	chr7_418688	NA	4248.935	3107.785
318	chr7_421152	hsa-miR-25-3p	1164.07	1585.79
319	chr7_421823	NA	6813.635	5582.875
320	chr7_423642	NA	2038.335	924.305
321	chr7_423917	NA	2284.31	2898.48
322	chr7_425927	hsa-miR-182-5p	766.49	1063.605
323	chr7_426422	hsa-miR-29a-3p	346.89	666.98
324	chr7_426428	hsa-miR-29b-3p	172.81	388.385

325	chr7_430719	NA	8519.42	5381.815
326	chr7_430867	NA	6934.475	5443.58
327	chr8_437436	NA	280.58	334.865
328	chr8_445431	NA	1249.565	2673.41
329	chr8_449083	NA	1530.145	758.25
330	chr8_450739	NA	613.135	642.965
331	chr8_452267	NA	2056.24	2622.63
332	chr8_453113	NA	1572.89	1484.24
333	chr8_453114	NA	1609.245	1232.41
334	chr8_454300	NA	819.735	758.25
335	chr8_454356	NA	1344.55	1368.955
336	chr8_454412	NA	477.96	361.62
337	chr8_454861	NA	13797.445	10613.345
338	chr8_456393	NA	65668.74	41976.57
339	chr8_459630	NA	545.55	503.67
340	chr8_467149	hsa-miR-30d-5p	819.735	1146.635
341	chr8_468218	hsa-miR-151a-3p	1601.575	1821.85
342	chr9_471735	hsa-miR-101-3p	883.49	476.91
343	chr9_472515	NA	2330.7	2619.885
344	chr9_472827	NA	226.055	251.835
345	chr9_473001	NA	1085.705	1671.565
346	chr9_477638	NA	301.315	447.4
347	chr9_481032	hsa-let-7f-5p	6787.515	6297.88
348	chr9_481559	hsa-miR-23b-3p	762.655	1055.365
349	chr9_481561	hsa-miR-27b-3p	2311.515	1256.42
350	chr9_481565	hsa-miR-24-3p	1333.04	1028.6
351	chr9_486631	NA	4098.685	2732.42
352	chr9_486754	hsa-miR-181a-5p	348.17	616.205
353	chr9_486756	hsa-miR-181b-5p	938.015	1419.735
354	chr9_487076	NA	22429.49	28115.435
355	chr9_490593	NA	1352.225	1642.06
356	chr9_491059	NA	6481.36	3935.325
357	chr9_491885	hsa-miR-126-3p	87.04	195.565
358	chr9_495866	NA	2012.215	1775.86
359	chr9_499731	NA	301.315	447.4
360	chr9_499943	NA	301.315	447.4
361	chr9_500023	NA	301.315	447.4
362	chr9_501941	hsa-miR-7-5p	8196.285	5397.585
363	chr9_507681	NA	6694.355	3908.56
364	chr9_508187	NA	904.22	835.785
365	chr9_511459	NA	122.12	195.565

366	chrX_1000062	hsa-miR-92a-3p	2224.2	2936.23
367	chrX_1000064	hsa-miR-19b-3p	68.865	139.3
368	chrX_1001721	hsa-miR-224-5p	1134.115	865.29
369	chrX_1001729	hsa-miR-452-5p	403.975	417.89
370	chrX_1002674	NA	159.745	305.355
371	chrX_969888	NA	176.645	139.3
372	chrX_972388	hsa-miR-532-5p	507.915	586.695
373	chrX_974360	NA	1066.525	2542.35
374	chrX_979246	NA	158.47	278.595
375	chrX_982631	NA	405.255	278.595
376	chrX_982672	NA	701.455	447.4
377	chrX_987263	NA	1062.69	530.425
378	chrX_989305	NA	12988.755	19116.68
379	chrX_989740	hsa-miR-221-3p	663.83	776.765
380	chrX_989742	hsa-miR-222-3p	346.89	364.37
381	chrX_994139	hsa-miR-374b-5p	85.765	222.33
382	chrX_994636	NA	491.02	696.485
383	chrX_997089	NA	6510.96	6868.805
384	chrX_999003	NA	1782.325	417.89
385	chrX_999004	NA	1782.325	417.89
386	GL383521.1_1029153	NA	1335.6	1390.225
387	KI270822.1_1037272	NA	477.96	361.62
388	KI270845.1_1015186	NA	1168.915	1446.49
389	KI270853.1_1018239	NA	8184.855	13415.77
390	KI270862.1_1021399	NA	153.35	139.3
391	KI270862.1_1021400	NA	293.645	391.135
392	KI270862.1_1021433	NA	1314.59	924.305
393	KI270910.1_1022355	NA	153.35	139.3
394	KI270910.1_1022389	NA	1314.59	924.305
395	KQ090013.1_1030120	NA	1231.39	2228.755

Appendix Table S2. List of miRNAs interacting with only Ago1x

	miRDeep2 ID	miRBase ID	Mean RPM in Ago1 IP samples (N=2)	Mean RPM in Ago1x IP samples (N=2)
1	chr17_833827	NA	0	195.565
2	chr19_871197	hsa-miR-181c-5p	0	139.3

Appendix Table S3. List of miRNAs interacting with only Ago1

	miRDeep2 ID	miRBase ID	Mean RPM in Ago1 IP samples (N=2)	Mean RPM in Ago1x IP samples (N=2)
1	chr10_528312	NA	70.145	0
2	chr11_598270	NA	35.07	0
3	chr12_613316	NA	105.22	0
4	chr12_642295	NA	107.775	0
5	chr15_734407	NA	89.6	0
6	chr16_785688	NA	71.425	0
7	chr17_814257	NA	124.675	0
8	chr17_829532	NA	53.245	0
9	chr17_834904	hsa-miR-152-3p	51.97	0
10	chr17_835854	NA	106.5	0
11	chr19_898325	NA	53.245	0
12	chr20_905353	NA	141.57	0
13	chr2_128048	NA	51.97	0
14	chr2_155319	NA	87.04	0
15	chr22_941895	NA	158.47	0
16	chr3_184321	NA	35.07	0
17	chr4_255334	NA	89.6	0
18	chr5_273898	NA	103.94	0
19	chr5_325091	hsa-miR-584-5p	71.425	0
20	chr6_360902	NA	35.07	0
21	chr7_397078	NA	53.245	0
22	chr7_405634	NA	176.645	0
23	chr7_413546	NA	70.145	0
24	chrX_1000105	hsa-miR-503-5p	87.04	0
25	chrX_1000106	hsa-miR-503-5p	87.04	0
26	chrX_994318	hsa-miR-374a-5p	103.94	0
27	GL000225.1_1007780	NA	158.47	0
28	KI270792.1_1031708	NA	103.94	0
29	KI270857.1_1023826	NA	53.245	0

RPM, Reads per million mapped miRNA reads

IP, Immunoprecipitation
