# Supplementary material for: Let‐7a‐regulated translational readthrough of mammalian AGO1 generates a microRNA pathway inhibitor
Source: EMBO J. 2019 Jul 22;38(16):e100727. doi: 10.15252/embj.2018100727 (PMC6694283; doi:10.15252/embj.2018100727)
Supplement: Supplementary file 2 — Expanded View Figures PDF [file EMBJ-38-e100727-s002.pdf]

Expanded View Figures

Figure EV1. Translational readthrough in *AGO1* (related to Fig 1).

A Comparison of efficiency of translational readthrough of *AGO1* with that of *VEGFA*. The assay was done as described in Fig 1C. $^{**}P = 0.0051$ (with Welch's correction); $^{***}P = 0.002$. Numbers indicate % of readthrough.

B Significance of the identity of the canonical stop codon in *AGO1* translational readthrough. This *in vitro* assay was done as described in Fig 1E. $^{***}P < 0.0001$.

C Demonstration of translational readthrough of *AGO1* using Myc-tag reporter assay. Plasmids containing in-frame *AGO1*-TGA (or GCA)-ISR-Myc/His were transfected in HEK293 cells. His-tagged proteins were enriched using the HisPur Ni-NTA resin, and the readthrough product was detected by Western blot.

Data information: Bar graphs (mean ± SE) are representative of at least two independent experiments. Statistical significance was calculated using Student's *t*-test. ISR, inter-stop codon region; ΔISR, construct without ISR.

© 2019 The Authors

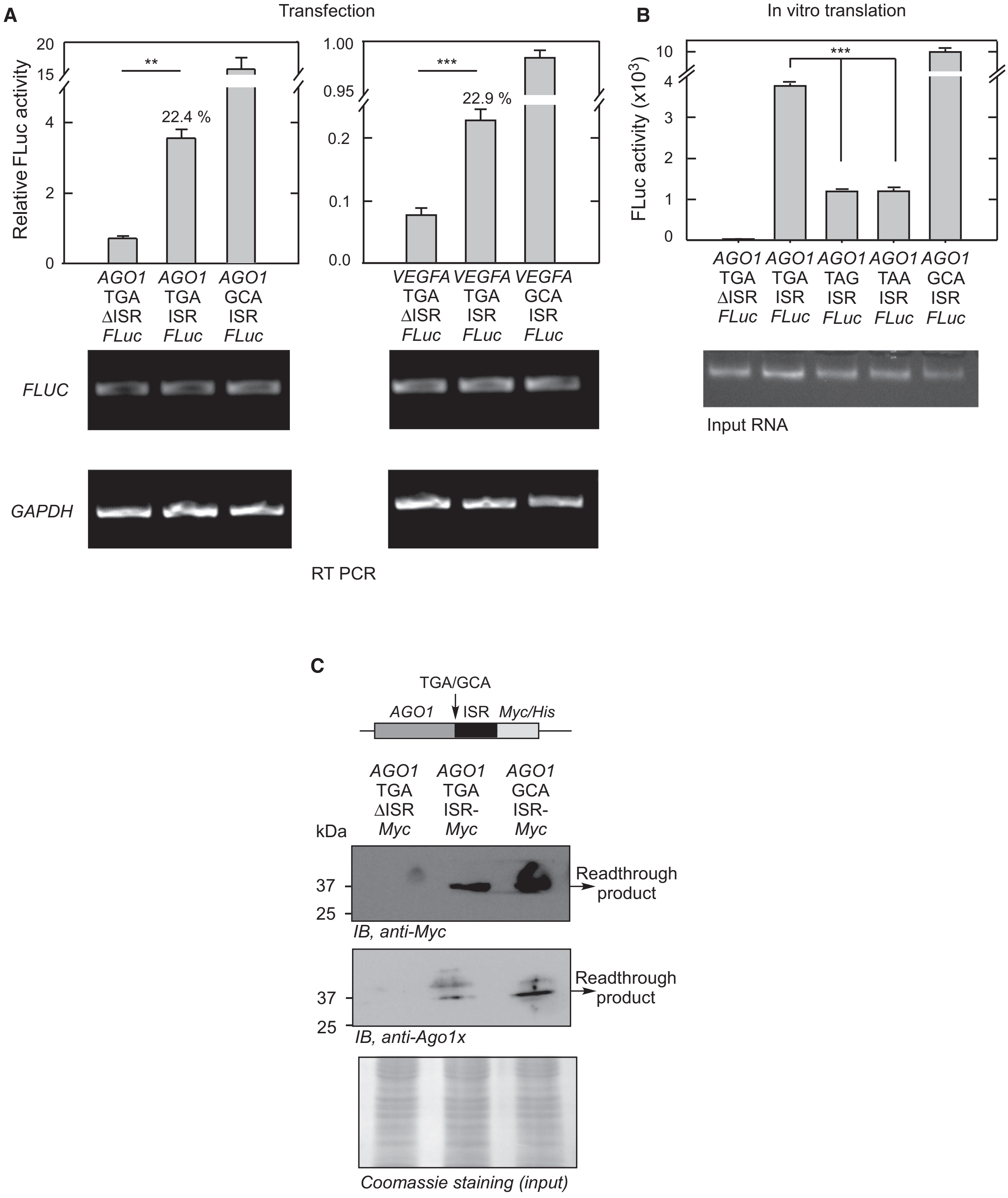

Figure EV1.

© 2019 The Authors

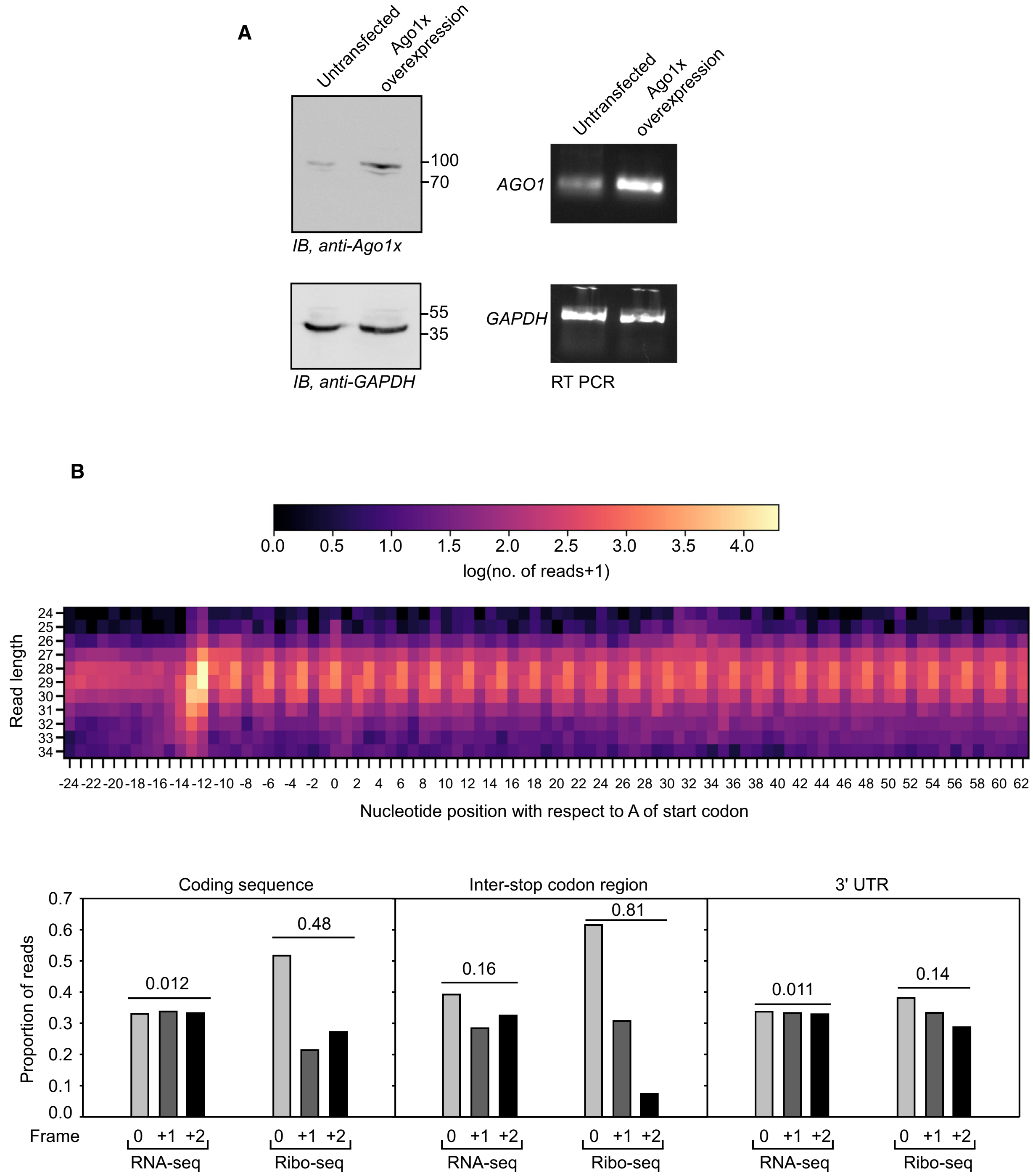

Figure EV2. Validation of anti-Ago1x antibody and analysis of 3-nucleotide periodicity in ribosome profiling data (related to Fig 2).

A Anti-Ago1x antibody can detect overexpressed Ago1x in HEK293 cells. RT–PCR results demonstrate the overexpression of *AGO1* transcript.

B Heat map showing the 3-nucleotide periodicity found in ribosome profiling data from U2-OS cells. 0 in *x*-axis represents A of the start codon. Graphs below show the proportion of RNA-seq and Ribo-seq (ribosome profiling) reads in three frames found in coding sequence, inter-stop codon region, and 3′UTR of *AGO1* transcript. Numbers on the bars indicate coefficient of variation (CV). Expectedly, RNA-seq reads showed a near-uniform distribution across the three frames (≈1/3 in each frame) as indicated by very low CV, i.e., 0.012, 0.16, and 0.011. The distribution of Ribo-seq reads in coding sequence and inter-stop codon region was non-uniform with a majority of the reads in the 0th frame (CV: 0.48 and 0.81). However, Ribo-seq reads in 3′UTR showed a near-uniform distribution (CV: 0.14) as it is an untranslated region.

Source data are available online for this figure.

© 2019 The Authors

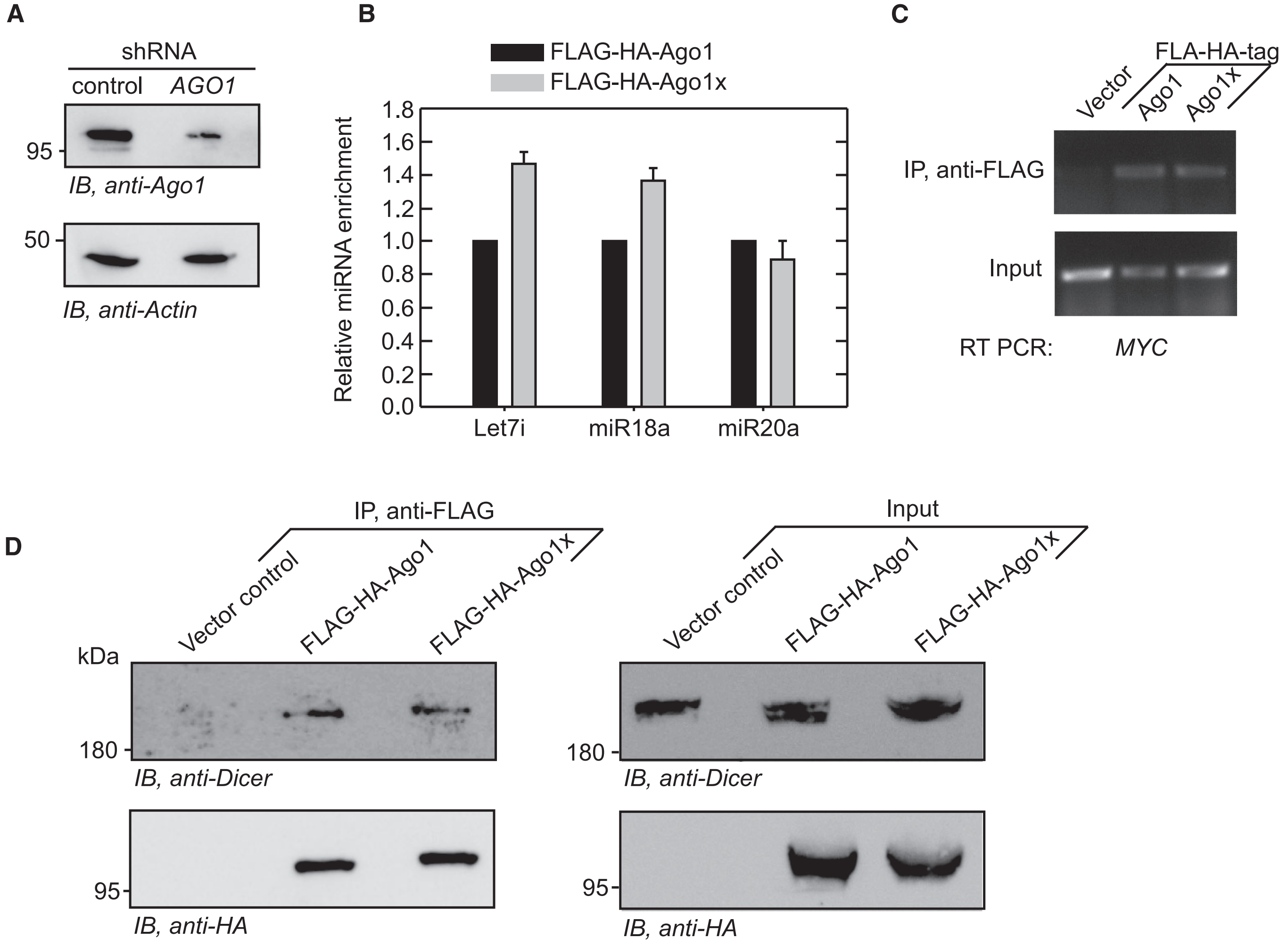

Figure EV3. Exogenous Ago1x can load miRNAs onto target mRNA in *AGO1* knockdown cells (related to Fig 5).

A Western blot showing the knockdown of *AGO1* in HeLa cells. Cells were stably transfected with shRNA that targets the distal 3′UTR of *AGO1*.

B qRT–PCR results showing the enrichment of three miRNAs in FLAG-HA-Ago1x immunoprecipitate relative to that in FLAG-HA-Ago1 immunoprecipitate in *AGO1* knockdown HeLa cells. Bars: mean ± SE (N = 3)

C RT–PCR results showing *MYC* mRNA co-immunoprecipitated with FLAG-HA-Ago1 or FLAG-HA-Ago1x in *AGO1* knockdown HeLa cells.

D Result of co-immunoprecipitation experiments demonstrating the interaction of Dicer protein with FLAG-HA-Ago1 and FLAG-HA-Ago1x in *AGO1* knockdown HeLa cells.

Source data are available online for this figure.

Figure EV4. Additional evidences to show that Ago1x does not interact with GW182 (related to Figs 6 and 7).

A Result of co-immunoprecipitation experiment in *AGO1* knockdown HeLa cells shows that GW182 is co-immunoprecipitated with FLAG-HA-Ago1, but not with FLAG-HA-Ago1x.

B Result of co-immunoprecipitation experiment in HeLa cells shows that FLAG-HA-Ago1, but not FLAG-HA-Ago1x, is co-immunoprecipitated with endogenous GW182.

C Confocal microscopy images of proximity ligation assay (PLA) in HeLa cells showing interaction of GW182 with Ago2, but not with Ago2ISR^{Ago1}. Rabbit anti-GW182 and mouse anti-HA were used to achieve proximity ligation. Interaction is indicated by red dots that result from proximity ligation. Quantification of dots per cell is shown (***P = 0.0001, Mann–Whitney test). Each black circle represents a microscopic field. Means ± SE are shown as horizontal lines (n = 7 fields for vector control; n = 14 fields for Ago2; n = 11 fields for Ago2ISR^{Ago1}). At least 80 cells were counted for dots in each category.

Source data are available online for this figure.

© 2019 The Authors

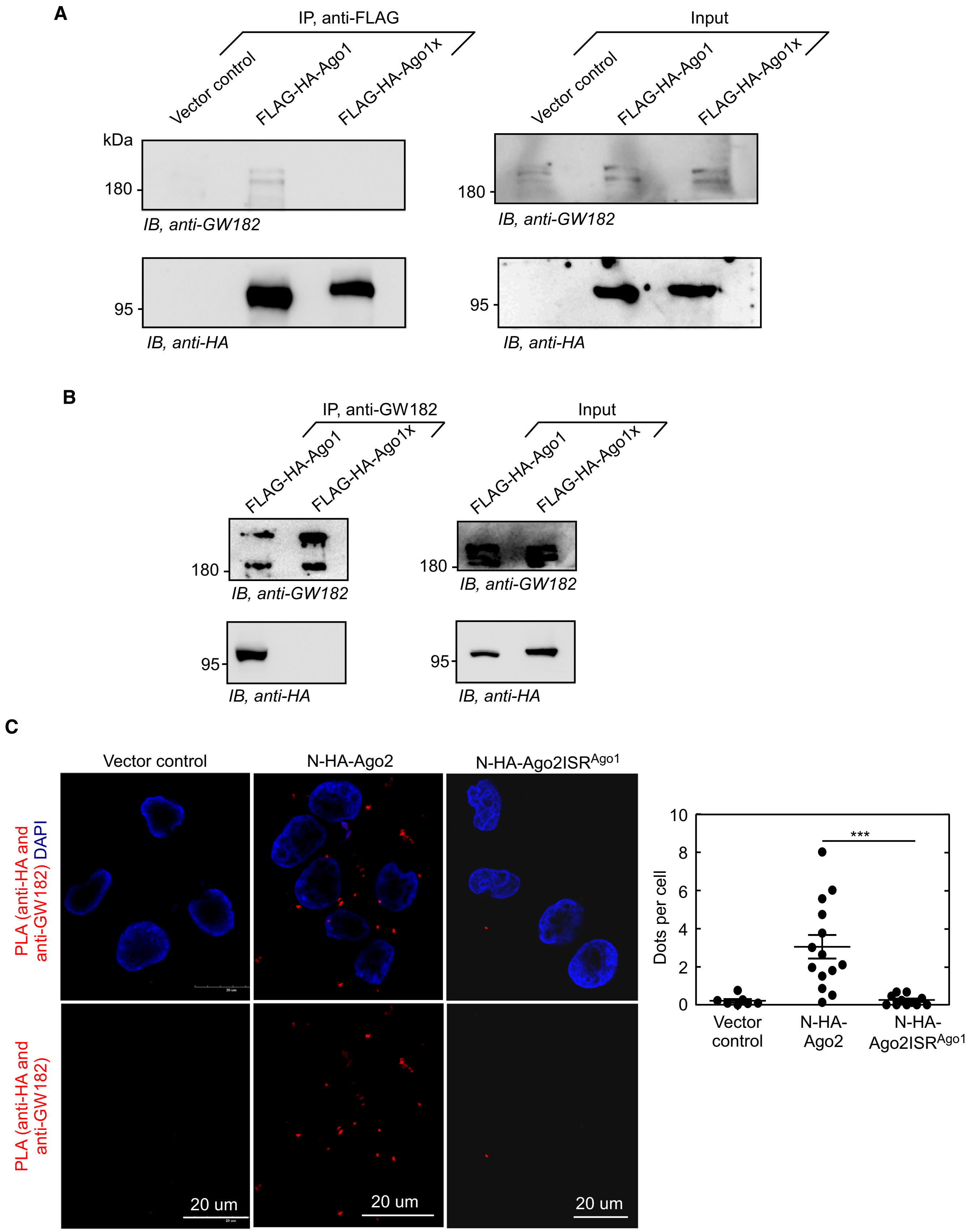
A
IP, anti-FLAG
Input
Vector control
FLAG-HA-Ago1
FLAG-HA-Ago1x
kDa
180
IB, anti-GW182
95
IB, anti-HA
B
IP, anti-GW182
Input
FLAG-HA-Ago1
FLAG-HA-Ago1x
180
IB, anti-GW182
95
IB, anti-HA
C
Vector control
N-HA-Ago2
N-HA-Ago2ISR Ago1
PLA (anti-HA and anti-GW182) DAPI
PLA (anti-HA and anti-GW182)
20 um
Dots per cell
***
Vector control
N-HA-Ago2
N-HA-Ago2ISR Ago1

Figure EV4.

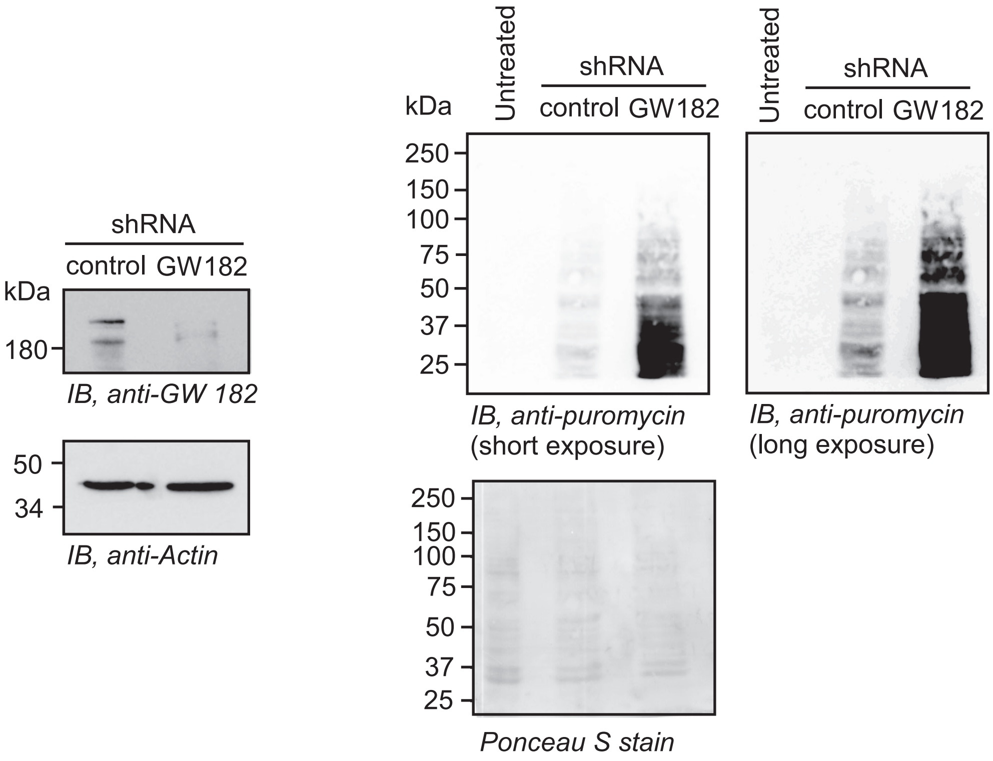

Figure EV5. Validation of ribopuromycylation (related to Fig 8).
Ribopuromycylation assay showing global translation profile of GW182 knockdown HeLa cells. GW182 knockdown was confirmed by Western blot (left).

Source data are available online for this figure.

 © 2019 The Authors
