# Supplementary material for: Let‐7a‐regulated translational readthrough of mammalian AGO1 generates a microRNA pathway inhibitor
Source: EMBO J. 2019 Jul 22;38(16):e100727. doi: 10.15252/embj.2018100727 (PMC6694283; doi:10.15252/embj.2018100727)
Supplement: Supplementary file 3 — Table EV1 [file EMBJ-38-e100727-s003.docx]

**Table EV1. Details of ribosome profiling datasets which showed positive evidence for translational readthrough of *AGO1***

| **Cell type** | **N** | **RPBM Values (Mean ± S.E.) (10^-3^)** | | | **Ratio of ISR to CDS** | **Ratio of ISR to**  **3′UTR** |
| --- | --- | --- | --- | --- | --- | --- |
|  |  | **CDS** | **ISR** | **3′UTR** |  |  |
| U2OS | 16 | 3.89 ± 0.86 | 0.41 ± 0.08 | 0.02 ± 0.006 | 0.11 | 20.5 |
| B cells | 2 | 1.49 ± 0.24 | 1.03 ± 0.35 | 0.14 ± 0.03 | 0.69 | 7.36 |
| HCT116 | 2 | 1.21 ± 0.13 | 0.36 ± 0.26 | 0.03 ± 0.002 | 0.30 | 12 |
| Huh-7 | 2 | 5.85 ± 2.21 | 5.06 ± 4.49 | 1.21 ± 1.17 | 0.86 | 4.18 |
| MCF7 | 2 | 1.48 ± 0.02 | 0.20 ± 0.04 | 0.01 ± 0.002 | 0.14 | 20 |
| HeLa | 1 | 1.83 | 0.03 | 0.002 | 0.02 | 15 |
| HEK293 | 1 | 1.62 | 0.40 | 0.03 | 0.25 | 13.333 |
| MEF | 1 | 1.56 | 0.34 | 0.008 | 0.22 | 42.5 |

CDS: coding sequence

ISR: Inter-stop codon region

N: number of datasets (Ribo-seq) showing evidence for readthrough

3'UTR excludes the ISR region

RPBM: Reads per base per million
