# Supplementary material for: Let‐7a‐regulated translational readthrough of mammalian AGO1 generates a microRNA pathway inhibitor
Source: EMBO J. 2019 Jul 22;38(16):e100727. doi: 10.15252/embj.2018100727 (PMC6694283; doi:10.15252/embj.2018100727)
Supplement: Supplementary file 4 — Table EV2 [file EMBJ-38-e100727-s004.docx]

**Table EV2. List of *AGO1*-derived peptides detected in proteomes of mouse organs**

| **Peptide sequence** | **Source** | **Score** | **Reference** |
| --- | --- | --- | --- |
| AVQVHQDTLR | Mouse brain | 60.518 | (Sharma K, et al 2015) |
| DGVPEGQLPQILHYELLAIR | Mouse brain | 44.729 |  |
| ELLIQFYK | Mouse brain | 73.039 |  |
| EVVEYMVQHFKPQIFGDR | Mouse brain | 69.148 |  |
| HTYLPLEVCNIVAGQR | Mouse brain | 100.4 |  |
| KLTDNQTSTMIK | Mouse brain | 179.19 |  |
| LGGINNILVPHQR | Mouse brain | 50.805 |  |
| LLANYFEVDIPK | Mouse brain | 94.297 |  |
| LTDNQTSTMIK | Mouse brain | 78.939 |  |
| NASYNLDPYIQEFGIK | Mouse brain | 133.15 |  |
| RPASHQTFPLQLESGQTVECTVAQYFK | Mouse brain | 116.24 |  |
| VLPAPILQYGGR | Mouse brain | 81.594 |  |
| WLAIVSWR | Mouse brain | 60.682 |  |
| YAQGADSVEPMFR | Mouse brain | 128.03 |  |
| YPHLPCLQVGQEQK | Mouse brain | 98.754 |  |
| YRVCNVTR | Mouse brain | 44.753 |  |
| QNAVTSLDRR**^#^** | Mouse brain | 26.384 |  |
| AVQVHQDTLR | Mouse muscle | 108.47 | (Deshmukh AS, et al 2015) |
| DAGMPIQGQPCFCK | Mouse muscle | 119.16 |  |
| ELLIQFYK | Mouse muscle | 132.96 |  |
| HTYLPLEVCNIVAGQR | Mouse muscle | 185.95 |  |
| LGGINNILVPHQR | Mouse muscle | 77.867 |  |
| TPVYAEVK | Mouse muscle | 74.301 |  |
| TSPQTLSNLCLK | Mouse muscle | 59.067 |  |
| YAQGADSVEPMFR | Mouse muscle | 130.01 |  |
| TMYFANR**^#^** | Mouse muscle | 16.154 |  |
| AVQVHQDTLRTM(ox)YFAYR**^#$^** | Mouse liver | 55.885 | (Azimifar SB, et al 2014) |

**^#^**generated after translational readthrough; specific to Ago1x isoform.

**^$^** this peptide was detected in five different mouse liver samples.
