# Supplementary material for: Let‐7a‐regulated translational readthrough of mammalian AGO1 generates a microRNA pathway inhibitor
Source: EMBO J. 2019 Jul 22;38(16):e100727. doi: 10.15252/embj.2018100727 (PMC6694283; doi:10.15252/embj.2018100727)
Supplement: Supplementary file 5 — Source Data for Expanded View [file EMBJ-38-e100727-s013.zip › embj2018100727-sup-0013-SDataEV/FIG_EV1_FINAL.pdf]

Fig EV1 A

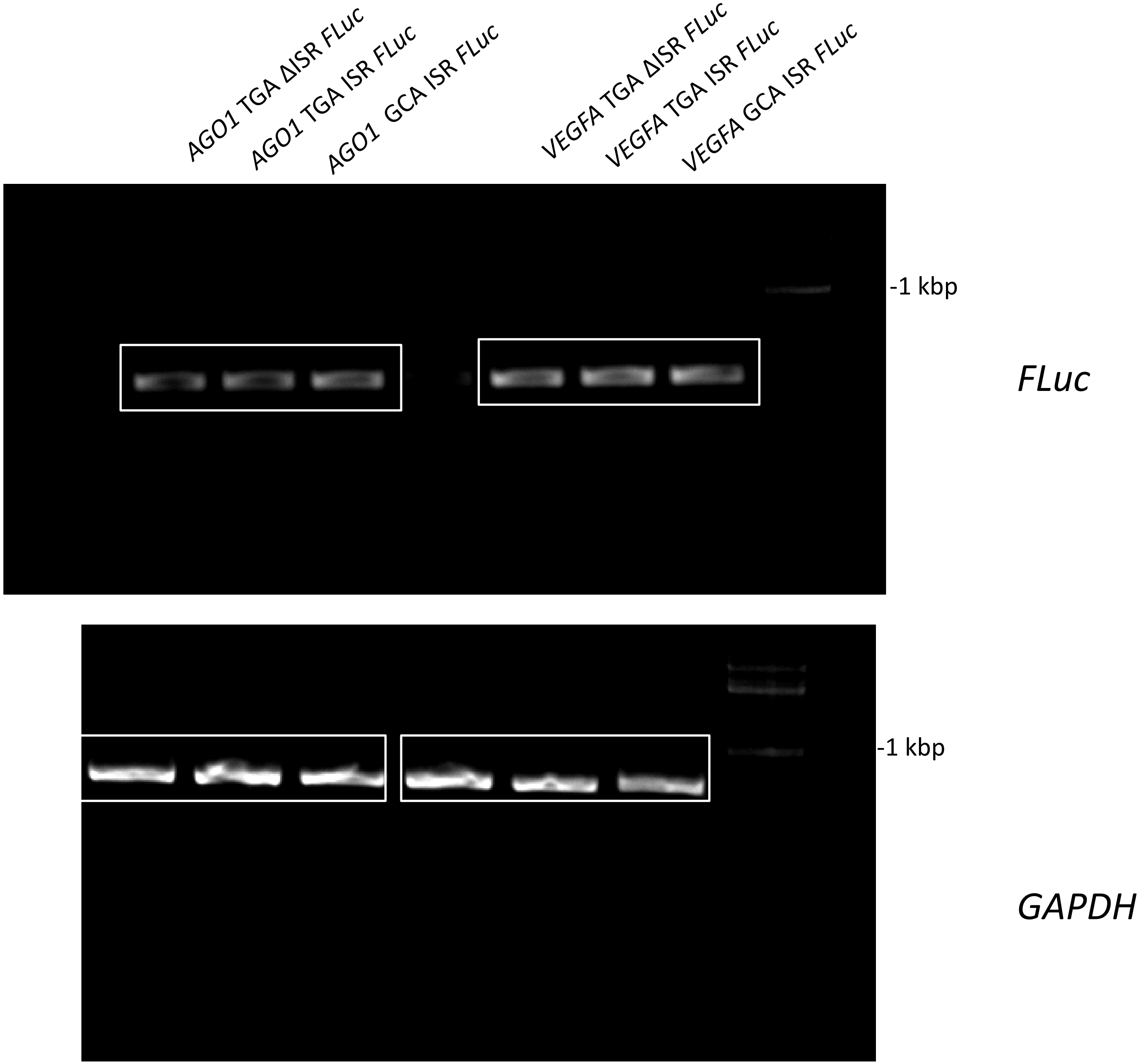
AGO1 TGA ΔISR FLuc
AGO1 TGA ISR FLuc
AGO1 GCA ISR FLuc
VEGFA TGA ΔISR FLuc
VEGFA TGA ISR FLuc
VEGFA GCA ISR FLuc
-1 kbp
FLuc
-1 kbp
GAPDH

Fig EV1 B

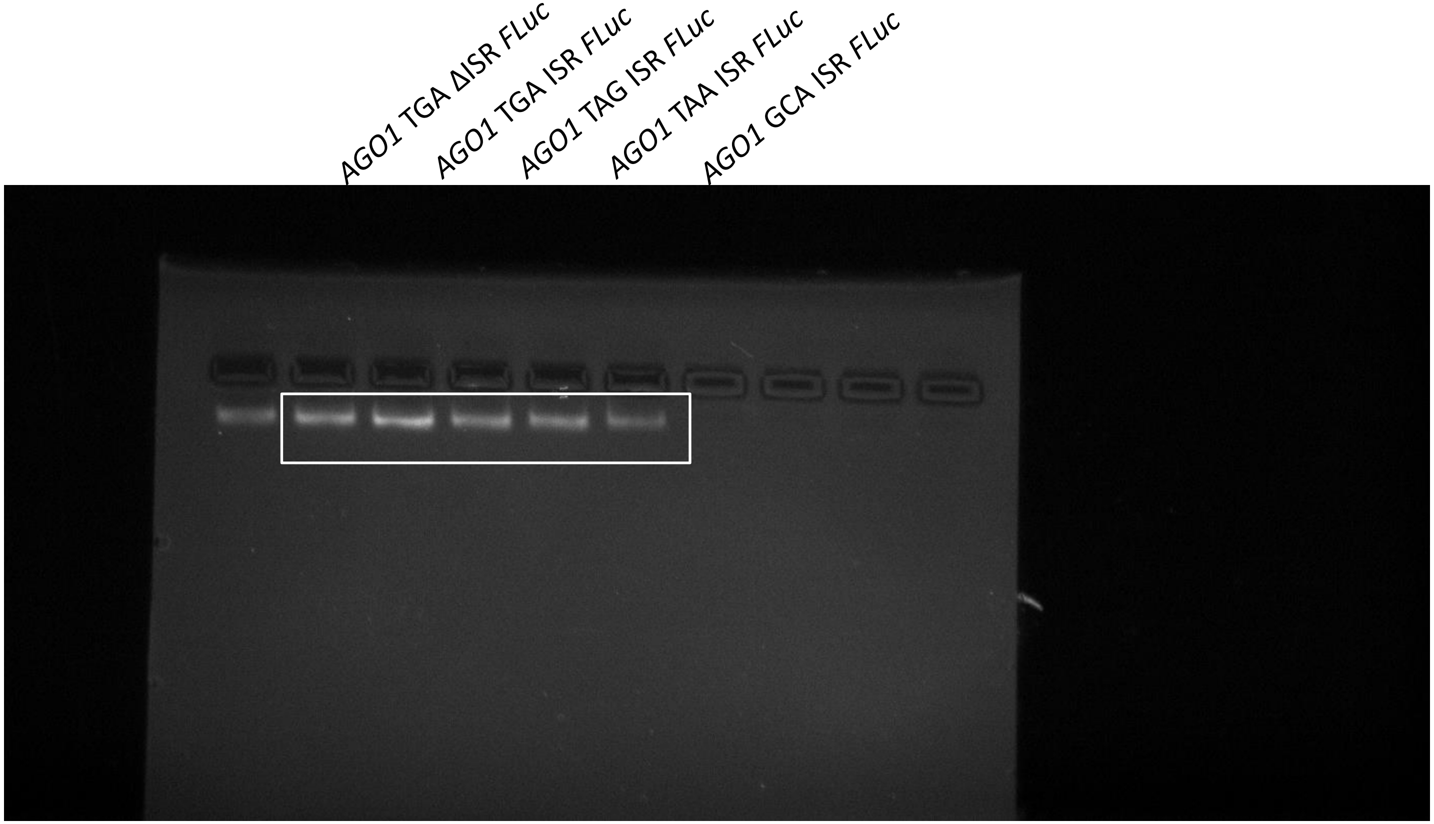

Input RNA

Fig EV1 C

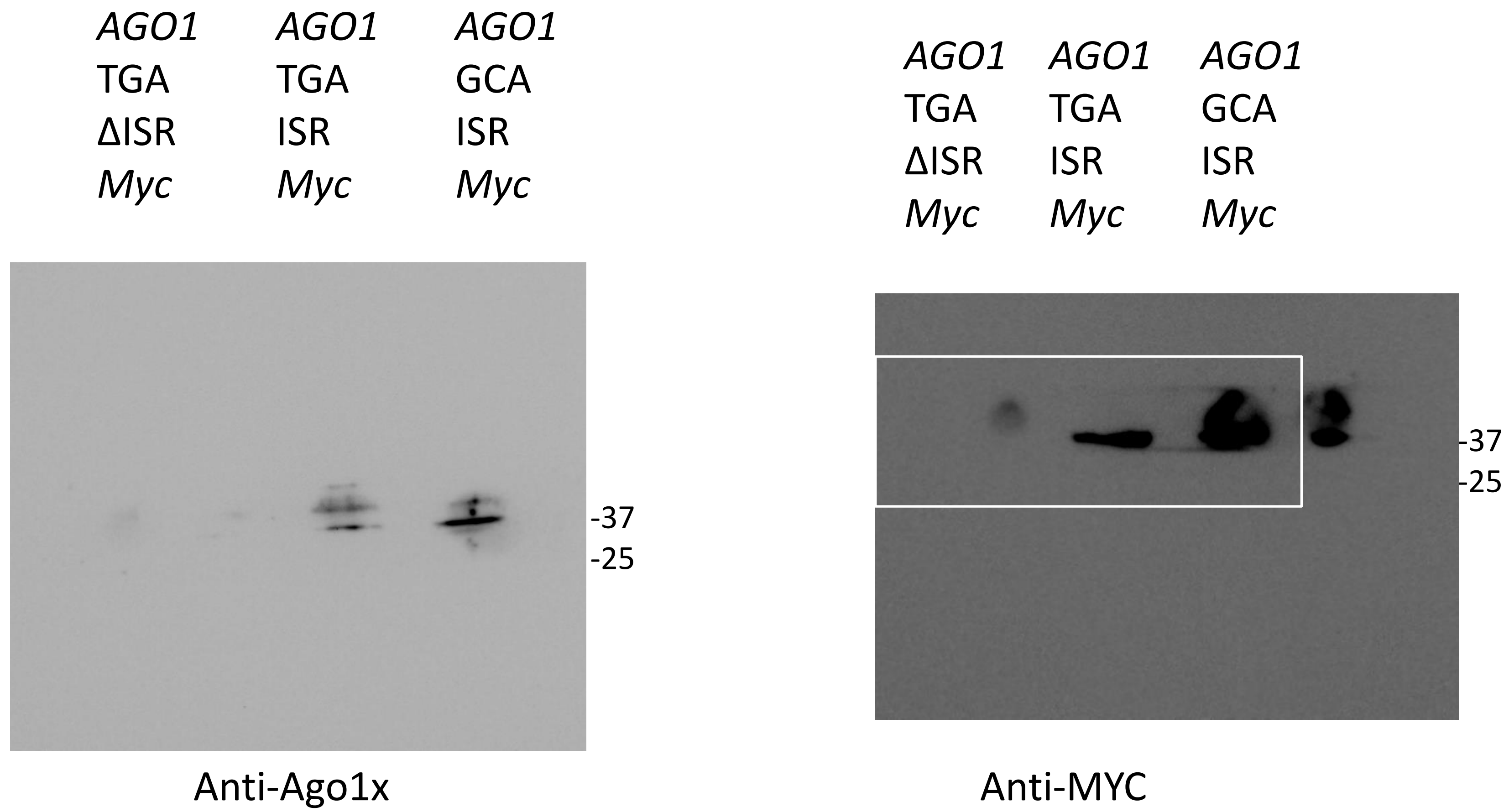
AGO1 TGA ΔISR Myc
AGO1 TGA ISR Myc
AGO1 GCA ISR Myc
-37
-25
Anti-Ago1x
AGO1 TGA ΔISR Myc
AGO1 TGA ISR Myc
AGO1 GCA ISR Myc
-37
-25
Anti-MYC

AGO1	*AGO1*	*AGO1*
TGA	TGA	GCA
ΔISR	ISR	ISR
Myc	*Myc*	*Myc*

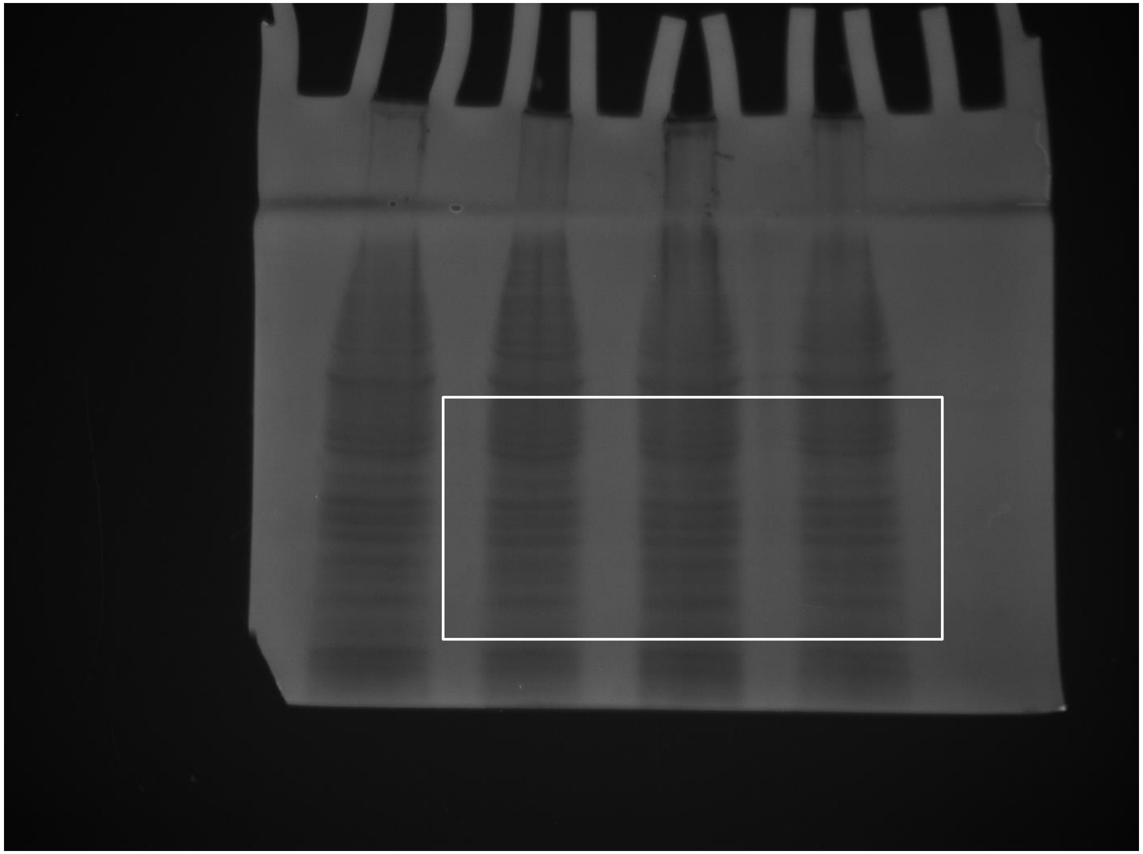

Coomassie

FIG_EV1_A						
	Rep-1	Rep-2	Rep-3	Average	SEM	P-value:
AGO1 TGA Delta ISR Fluc	0.6025	0.6995	0.8324	0.711454	0.0666	
AGO1 TGA ISR Fluc	3.1301	3.537	3.997	3.554713	0.2504	0.0051
AGO1 GCA ISR Fluc	14.1339	19.3694	14.0622	15.85519	1.7573	
VEGFA TGA DELTA ISR	0.054054	0.082456	0.090857	0.075789	0.0111	
VEGFA TGA ISR FLUC	0.221687	0.197143	0.257576	0.225468	0.0175	0.002
VEGFA GCA ISR FLUC	0.99	0.976471		0.983235	6.76E-03	

FIG_EV1_B						
	Rep-1	Rep-2	Rep-3	Average	SEM	P-value:
AGO1 TGA DELTA ISR FLUC	27	26	24	25.6667	0.8819	
AGO1 TGA ISR FLUC	3900	3700	3700	3766.667	66.6667	
AGO1 TAG ISR FLUC	1200	1300	1070	1190	6.6667	<0.0001
AGO1 TAA ISR FLUC	1340	893	1350	1194.333	66.5833	<0.0001
AGO1 GCA ISR FLUC	9980	10000	10000	9993.333	150.6943	
