# Supplementary material for: Let‐7a‐regulated translational readthrough of mammalian AGO1 generates a microRNA pathway inhibitor
Source: EMBO J. 2019 Jul 22;38(16):e100727. doi: 10.15252/embj.2018100727 (PMC6694283; doi:10.15252/embj.2018100727)
Supplement: Supplementary file 5 — Source Data for Expanded View [file EMBJ-38-e100727-s013.zip › embj2018100727-sup-0013-SDataEV/FIGURE_EV4.pdf]

Fig EV4 A

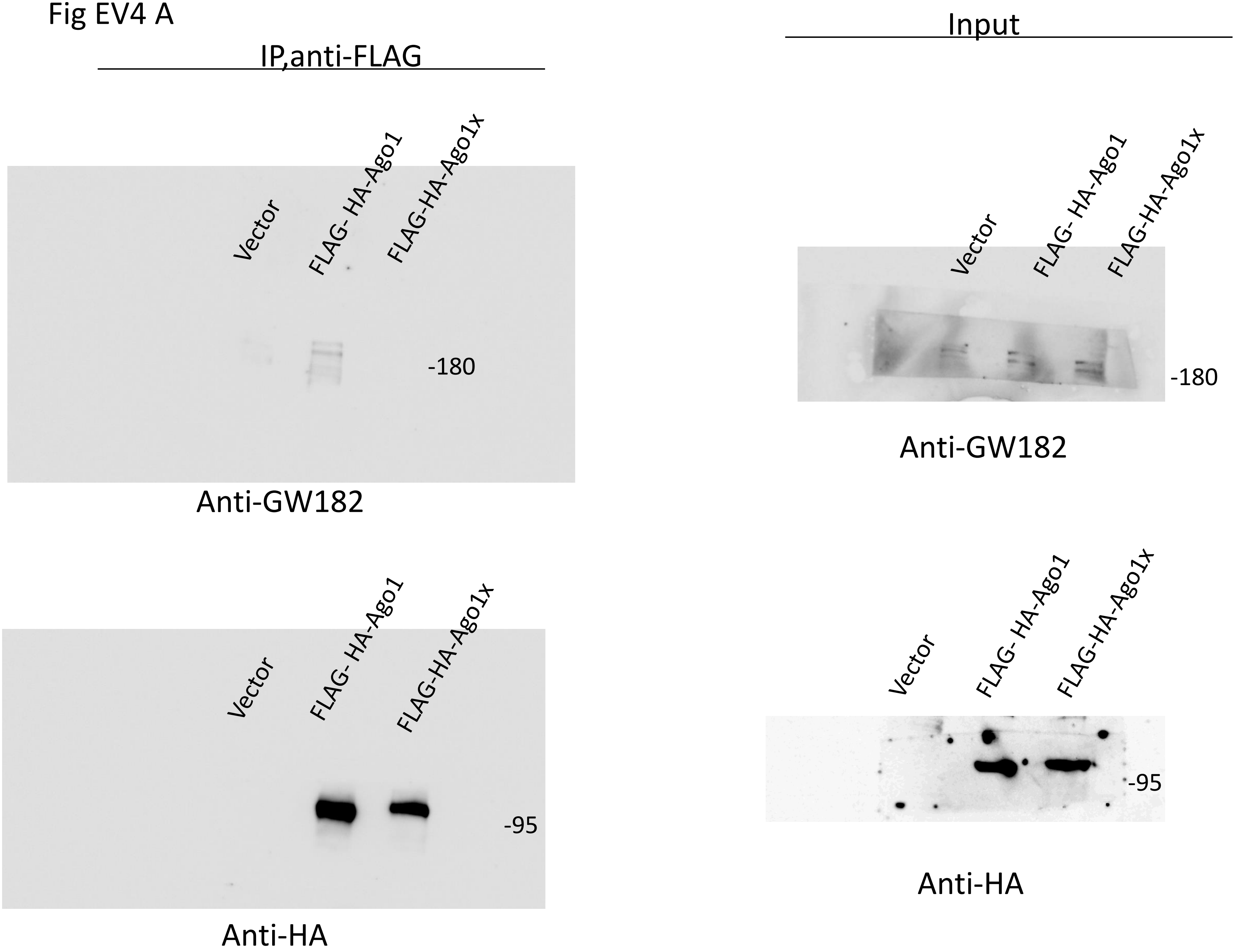

IP,anti-FLAG
Input
Vector
FLAG- HA-Ago1
FLAG-HA-Ago1x
-180
Anti-GW182
Vector
FLAG- HA-Ago1
FLAG-HA-Ago1x
-180
Anti-GW182
Vector
FLAG- HA-Ago1
FLAG-HA-Ago1x
-95
Anti-HA
Vector
FLAG- HA-Ago1
FLAG-HA-Ago1x
-95
Anti-HA

Fig EV4 B

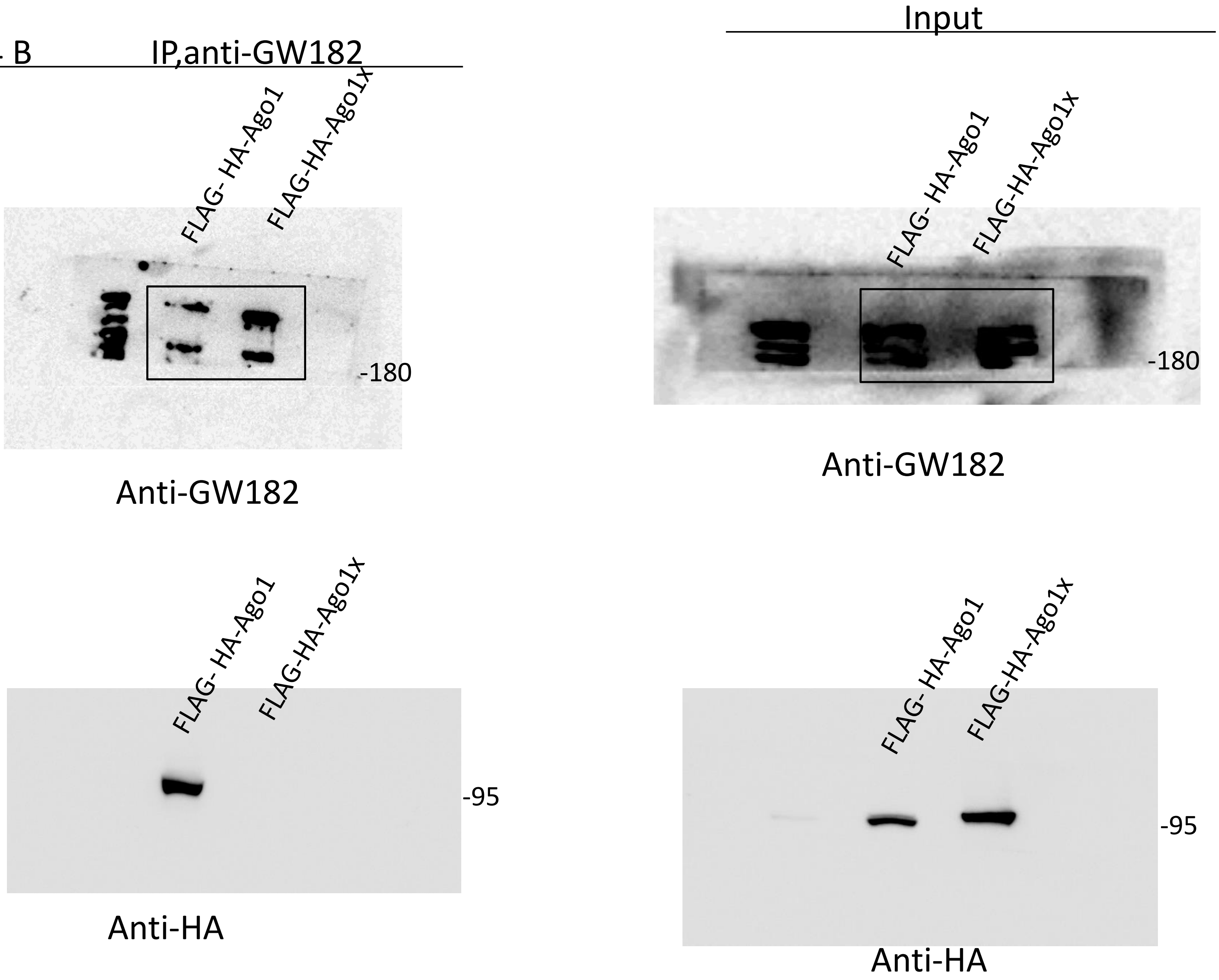
IP,anti-GW182
Input
FLAG- HA-Ago1
FLAG-HA-Ago1x
-180
Anti-GW182
-95
Anti-HA

FIG_EV4_C													
		vector					nha ago2					n ha ago2 isr ago1	
Microscopic field	No. of cells	No. of particles	particles/cell		Microscopic field	No. of cells	No. of particles	Particles/cell		Microscopic field	No. of cells	No. of particles	Particles/cell
1	23	2	0.086956522		1	20	17	0.85		1	8	0	0
2	19	5	0.263157895		2	2	6	3		2	3	2	0.666666667
3	18	4	0.222222222		3	21	41	1.952380952		3	6	0	0
4	15	1	0.066666667		4	2	3	1.5		4	3	2	0.666666667
5	23	2	0.086956522		5	1	8	8		5	3	0	0
6	20	0	0		6	9	50	5.555555556		6	8	1	0.125
7	4	3	0.75		7	2	12	6		7	12	0	0
					8	2	1	0.5		8	9	0	0
					9	8	1	0.125		9	11	5	0.454545455
					10	5	9	1.8		10	11	6	0.545454545
					11	8	21	2.625		11	9	3	0.333333333
					12	11	52	4.727272727					
					13	8	30	3.75					
					14	10	21	2.1					
	average	sem	p value										
vector	0.2109	0.0963	<0.0001	Mann Whitney test									
nha ago2	3.0347	0.6154											
nha ago2isr ago1	0.2538	0.0859											
