# Supplementary material for: Let‐7a‐regulated translational readthrough of mammalian AGO1 generates a microRNA pathway inhibitor
Source: EMBO J. 2019 Jul 22;38(16):e100727. doi: 10.15252/embj.2018100727 (PMC6694283; doi:10.15252/embj.2018100727)

Fig EV5

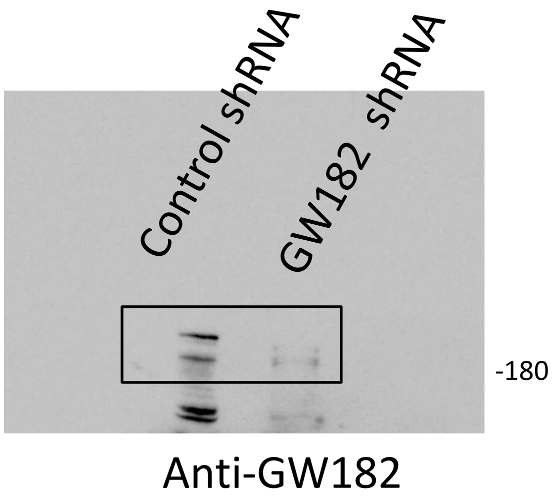
Control shRNA
GW182 shRNA
-180
Anti-GW182

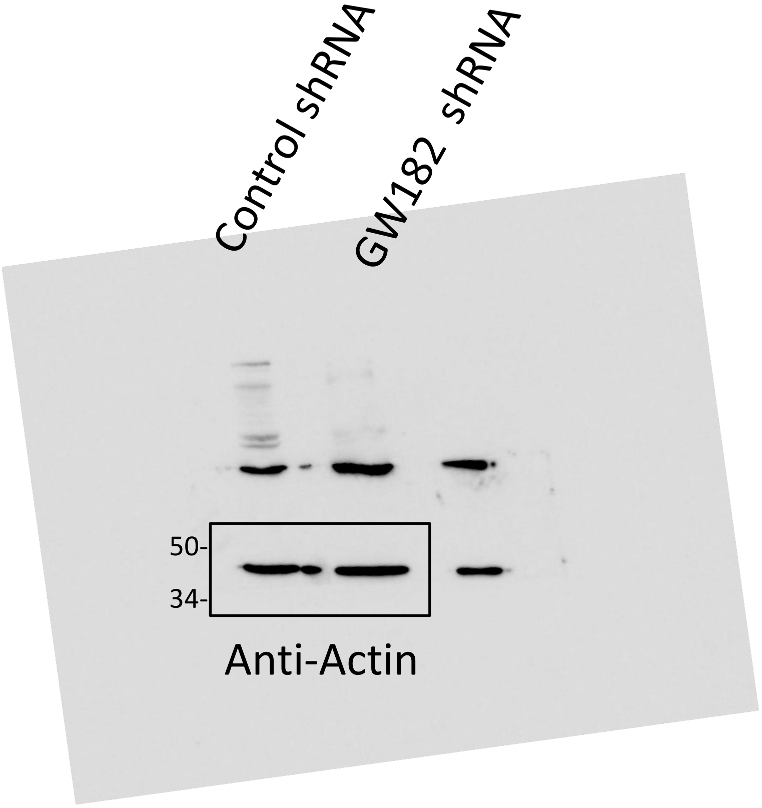
Control shRNA
GW182 shRNA
50-
34-
Anti-Actin

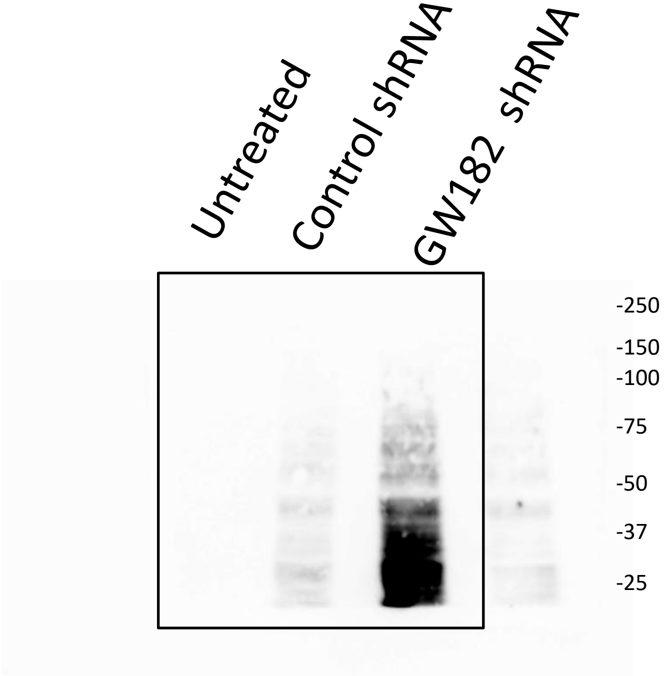

Anti-puromycin

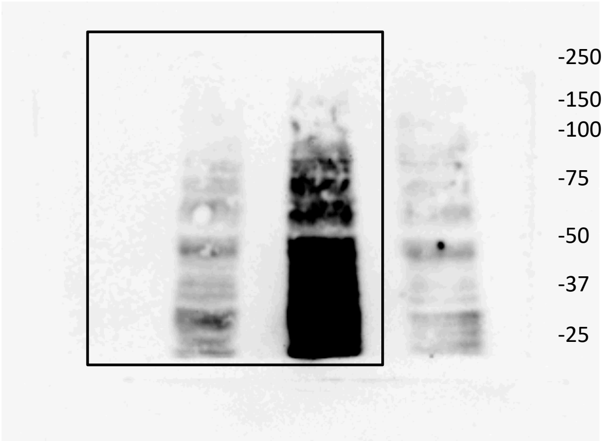

Anti-puromycin

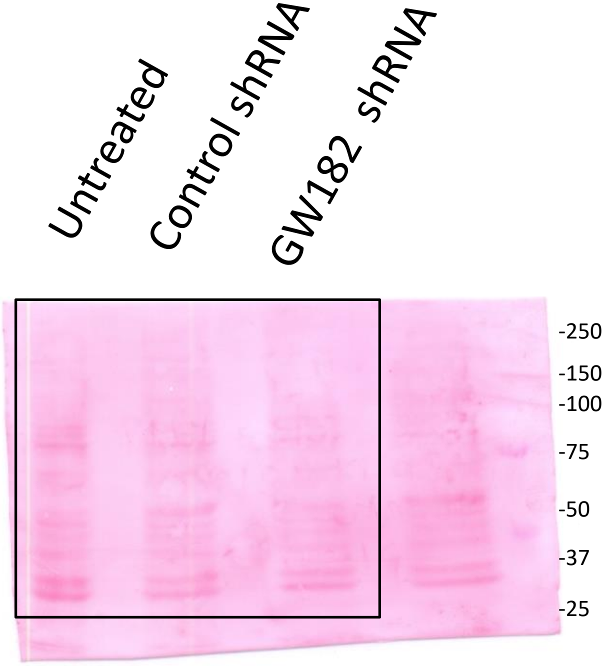

Supplement: Supplementary file 5 — Source Data for Expanded View [file EMBJ-38-e100727-s013.zip › embj2018100727-sup-0013-SDataEV/FIGURE_EV5.pdf]
