# Supplementary figures and images for: Let‐7a‐regulated translational readthrough of mammalian AGO1 generates a microRNA pathway inhibitor
Source: EMBO J. 2019 Jul 22;38(16):e100727. doi: 10.15252/embj.2018100727 (PMC6694283; doi:10.15252/embj.2018100727)

FIG EV2 A

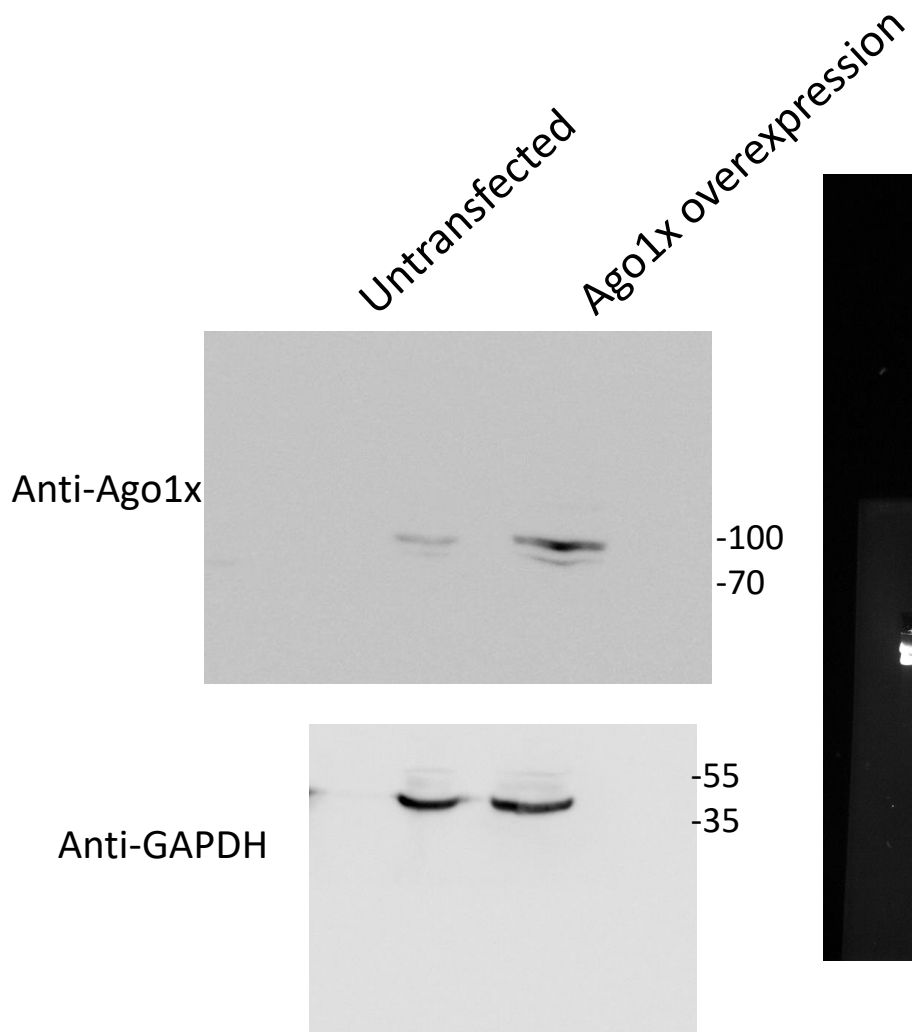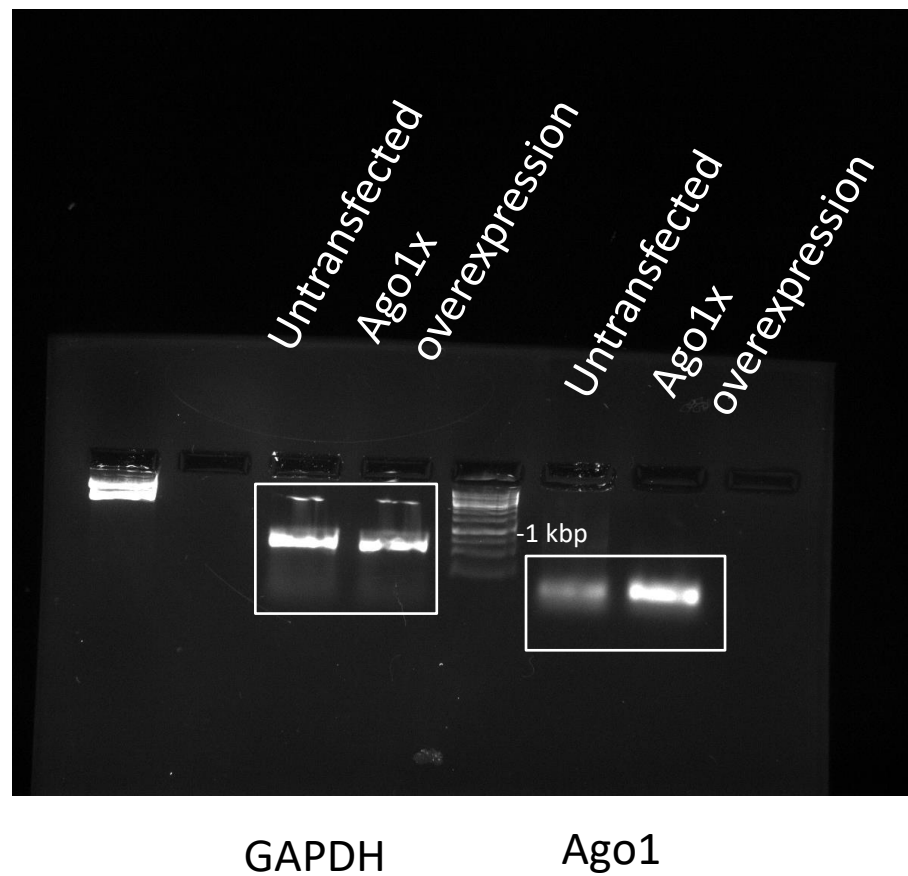

Supplement: Supplementary file 5 — Source Data for Expanded View [file EMBJ-38-e100727-s013.zip › embj2018100727-sup-0013-SDataEV/FIGURE_EV2.pdf]

Fig EV3 A

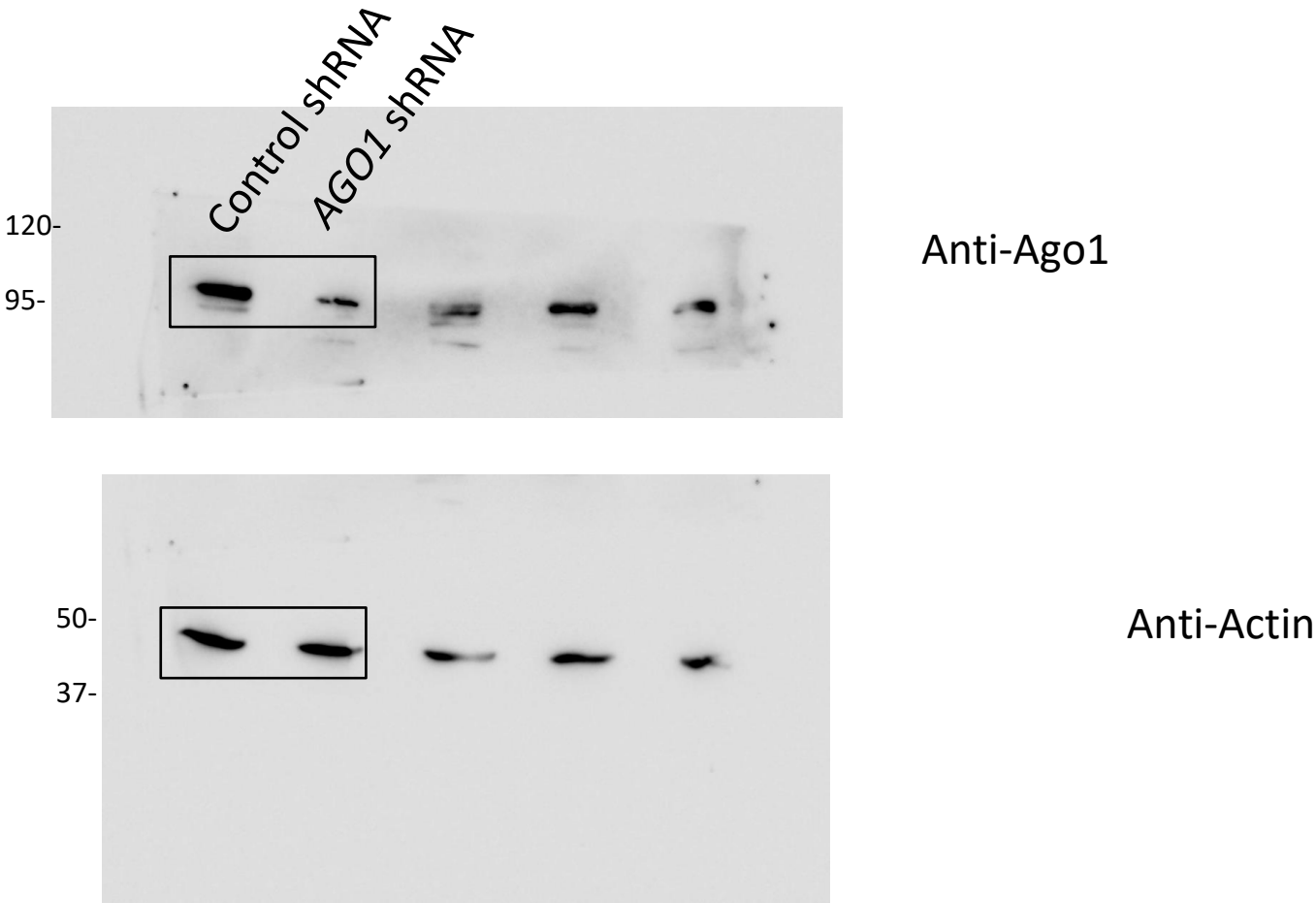

Fig EV3 C

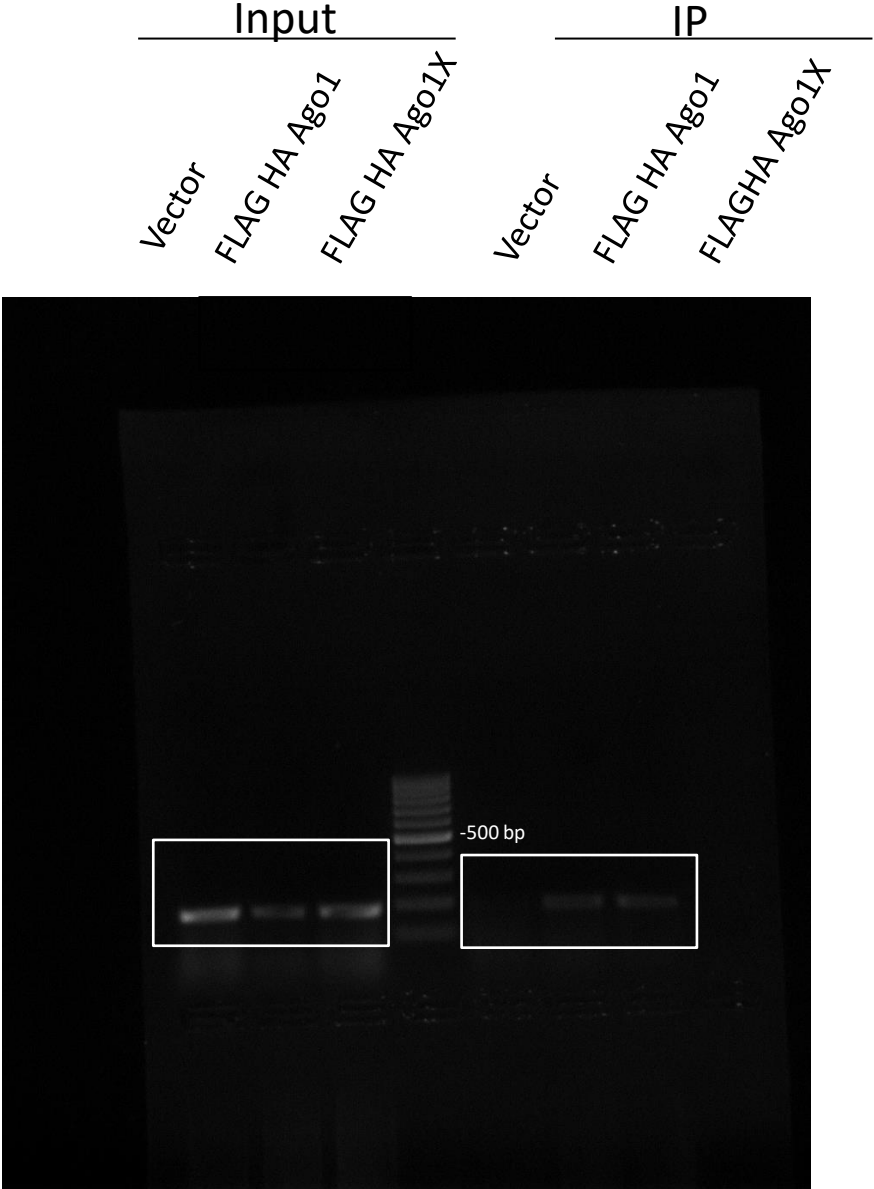

MYC

Fig EV3 D

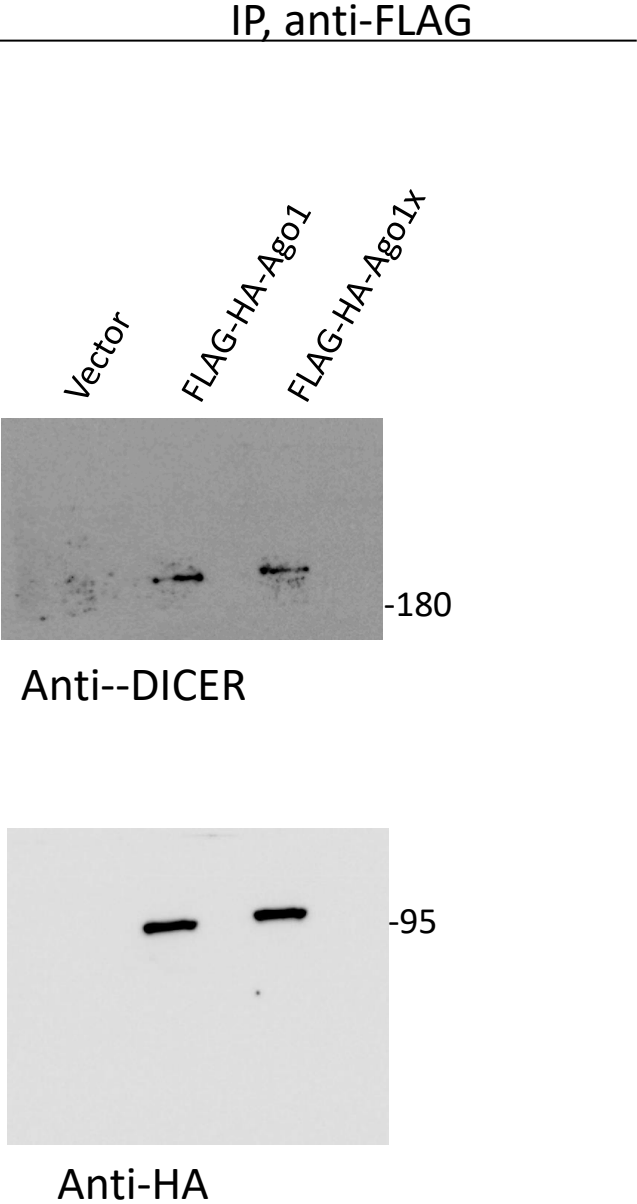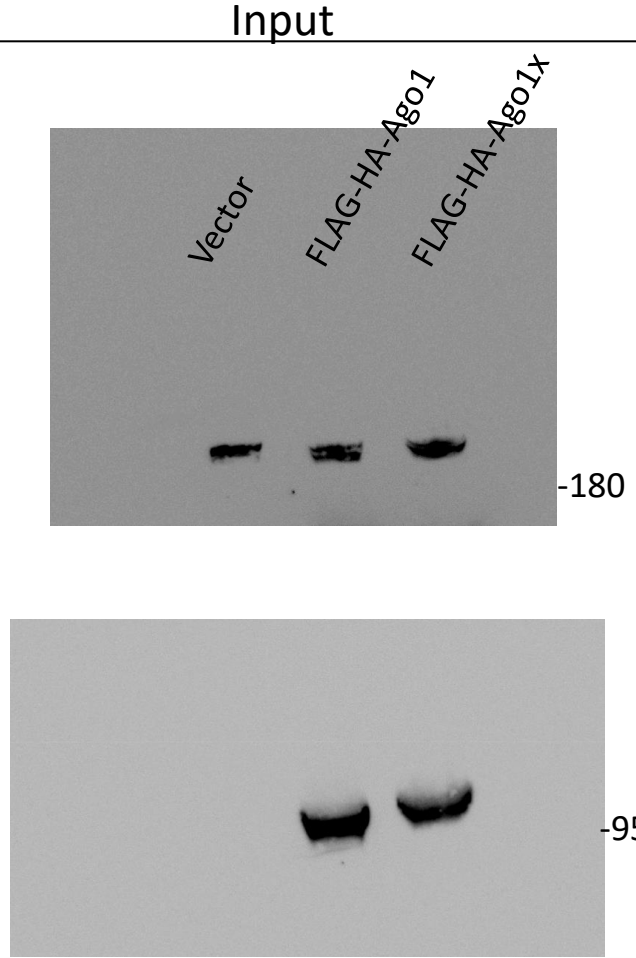

Supplement: Supplementary file 5 — Source Data for Expanded View [file EMBJ-38-e100727-s013.zip › embj2018100727-sup-0013-SDataEV/FIGURE_EV3.pdf]

Fig 5 C

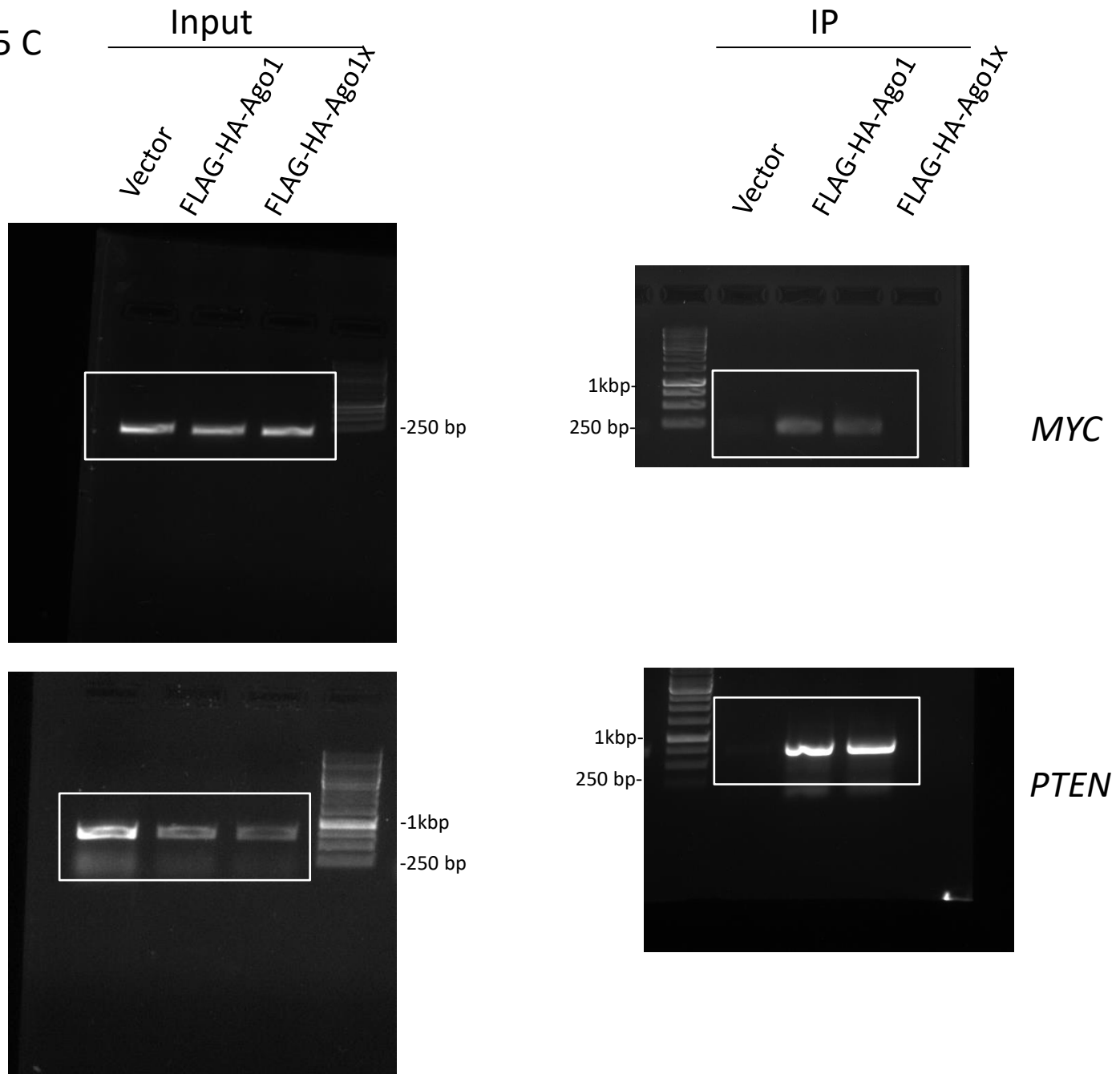

Fig 5 D

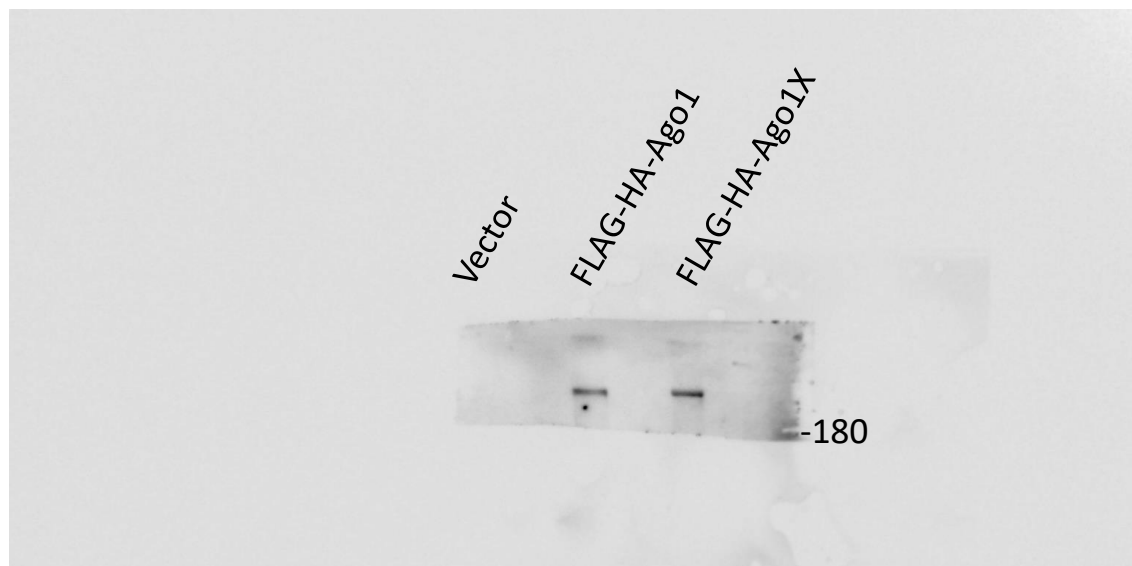

ANTI-DICER

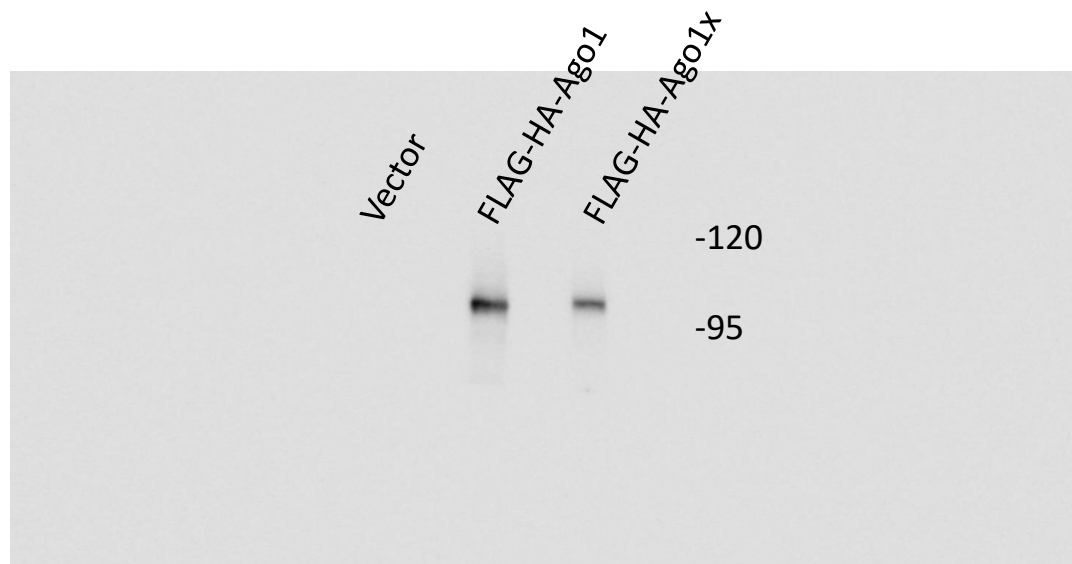

ANTI-HA

Fig 5 D

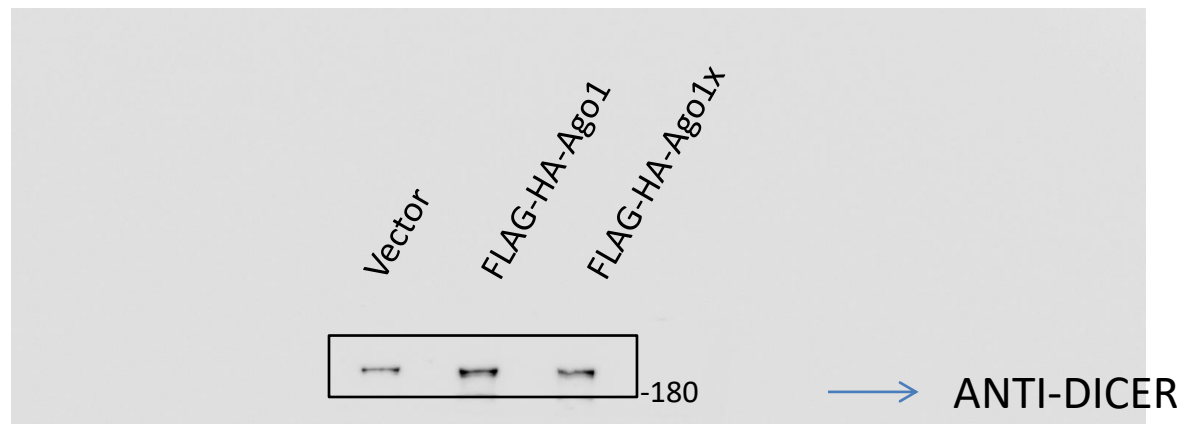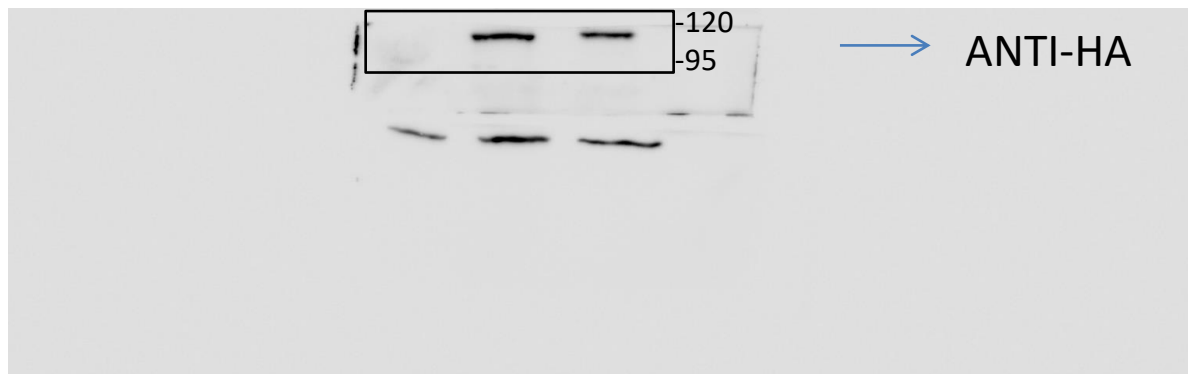

Supplement: Supplementary file 11 — Source Data for Figure 5 [file EMBJ-38-e100727-s009.pdf]
