# Supplementary material for: Let‐7a‐regulated translational readthrough of mammalian AGO1 generates a microRNA pathway inhibitor
Source: EMBO J. 2019 Jul 22;38(16):e100727. doi: 10.15252/embj.2018100727 (PMC6694283; doi:10.15252/embj.2018100727)
Supplement: Supplementary file 7 — Source Data for Figure 1 [file EMBJ-38-e100727-s005.pdf]

Fig 1 C

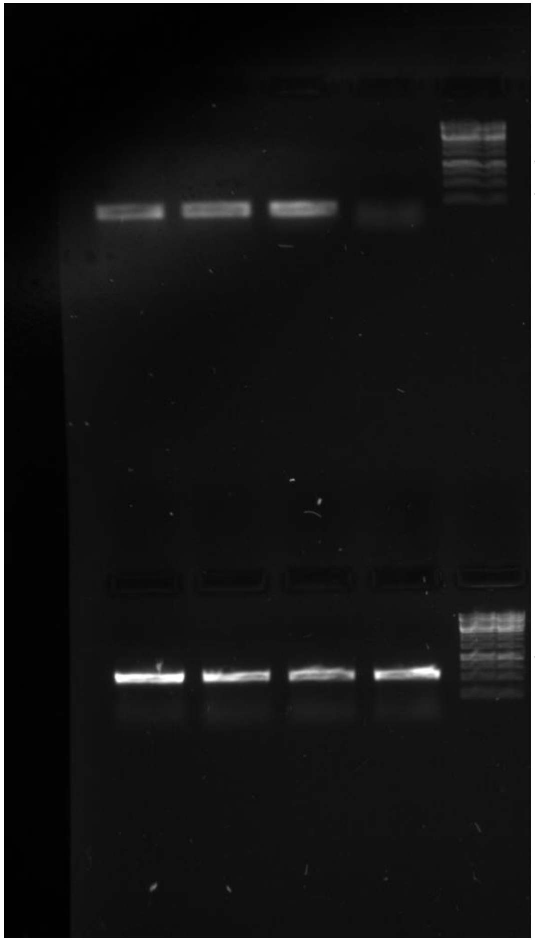

AGO1 TGA ΔISR *Fluc*
AGO1 TGA ISR *Fluc*
AGO1 GCA ISR *Fluc*
Only *RLuc*
-1 Kbp
-250 bp
FLuc
-1 Kbp
-250 bp
GAPDH

Fig 1 D

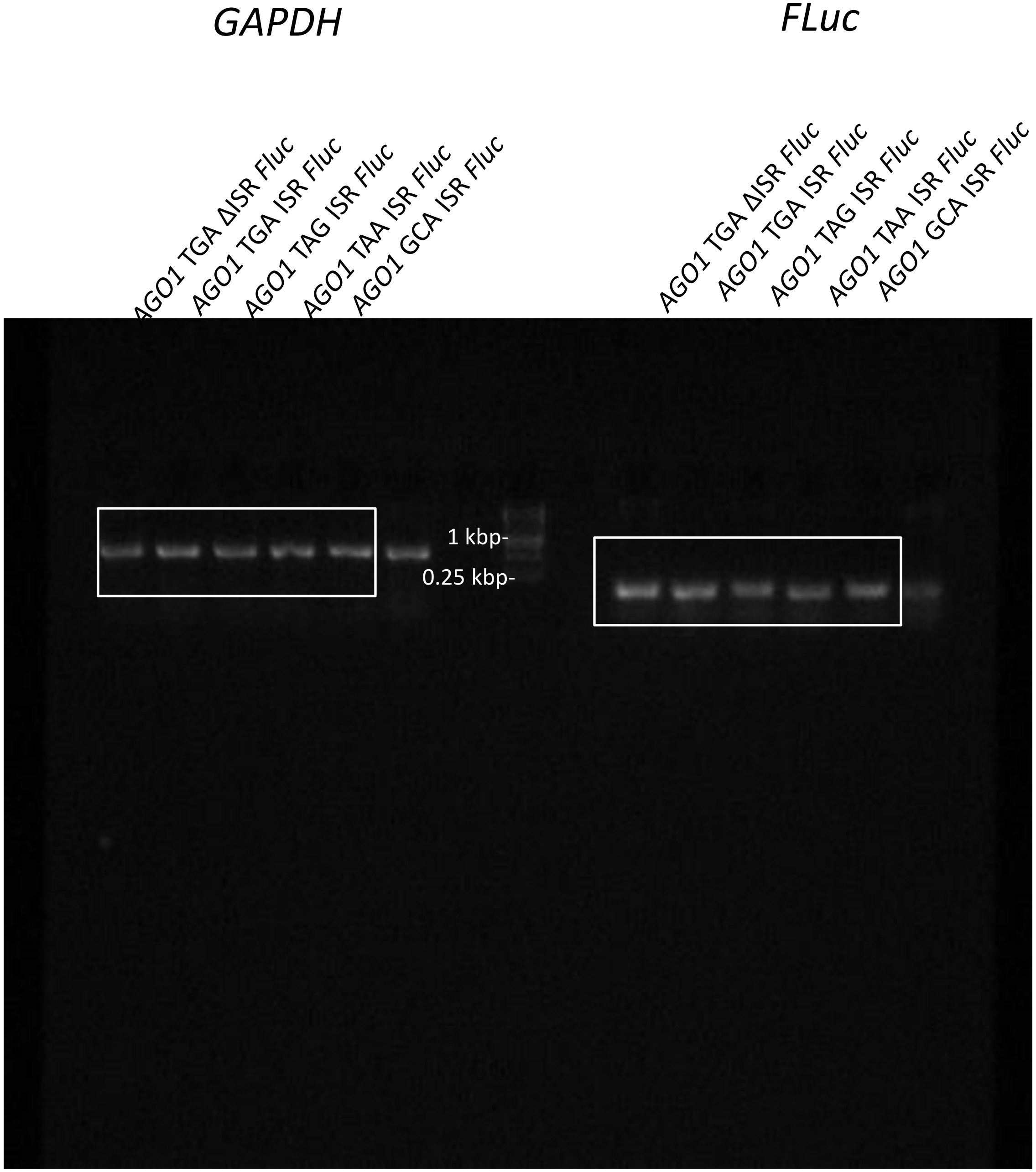

GAPDH
FLuc
AGO1 TGA ΔISR Fluc
AGO1 TGA ISR Fluc
AGO1 TAG ISR Fluc
AGO1 TAA ISR Fluc
AGO1 GCA ISR Fluc
AGO1 TGA ΔISR Fluc
AGO1 TGA ISR Fluc
AGO1 TAG ISR Fluc
AGO1 TAA ISR Fluc
AGO1 GCA ISR Fluc
1 kbp-
0.25 kbp-

Fig 1 E

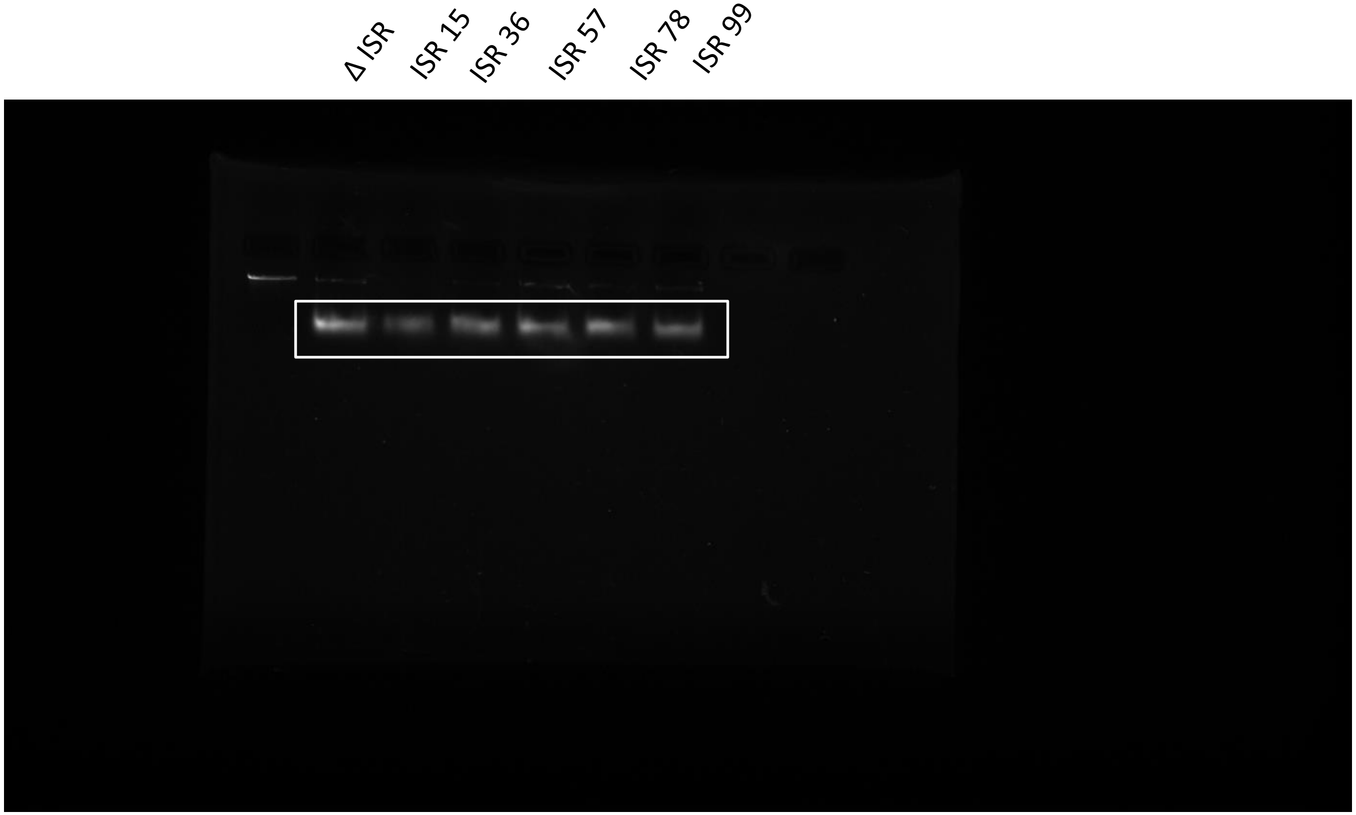

FIG_1_C				
	Fluc/Rluc	Average	SEM	
AGO1 TGA ISR Fluc	2.468487			
	2.534009	2.356964	0.1455	
	2.068396			
AGO1 GCA ISR Fluc	9.529091			
	9.243573	9.767424	0.3899	
	10.52961			
AGO1 TGA Delta ISR Fluc	0.163424			
	0.173405	0.142104	0.0265	
	0.089482			
only Rluc	0.131902	0.126549	3.03E-03	
	0.121429			
	0.126316			
P-value:	0.0001			

FIG_1_D			
	**Fluc/Rluc**	**Average**	**SEM**
**AGO1 TGA Delta ISR Fluc**	0.135218818	0.143158892	3.97E-03
	0.147237893		
	0.147019965		
**AGO1 TGA ISR Fluc**	1.822185133	1.985560101	0.1
	1.967191884		
	2.167303285		
**AGO1 GCA ISR Fluc**	4.461999689	5.794930634	0.7175
	6.001241745		
	6.92155047		
**AGO1 TAG ISR Fluc**	0.496834376	0.569640122	0.0486
	0.55038297		
	0.66170302		
**AGO1 TAA ISR Fluc**	0.523809524	0.591544973	0.084
	0.492350103		
	0.758475291		
	**P-value:**		
TGA and TAA	0.0004		
TGA and TAG	0.0002		

FIG_1_E					
	**Rep-1**	**Rep-2**	**Rep-3**	**Average**	**SEM**
**delta isr**	20	18	16	18	1.1547
**isr15**	21	19	18	19.3333	0.8819
**isr36**	65	41	32	46	9.8489
**isr57**	791	492	331	538	134.7677
**isr78**	516	506	445	489	22.1886
**isr99**	447	242	248	312.3333	67.3556
**P-value:**					
delta isr	0.012				
isr15	0.012				
isr36	0.017				

FIG_1_G					
	Rep-1	Rep-2	Rep-3	Average	SEM
Ago1 tga delta isr gfp	468	493	507	489.3333	11.4066
ago1 tga isr gfp	1664	1647	2498	1936.3333	280.8762
ago1 gca isr gfp	7150	5601	6415	6388.6667	447.3516
P-value:	0.007				
