# Supplementary material for: Let‐7a‐regulated translational readthrough of mammalian AGO1 generates a microRNA pathway inhibitor
Source: EMBO J. 2019 Jul 22;38(16):e100727. doi: 10.15252/embj.2018100727 (PMC6694283; doi:10.15252/embj.2018100727)
Supplement: Supplementary file 8 — Source Data for Figure 2 [file EMBJ-38-e100727-s006.pdf]

Fig 2 B

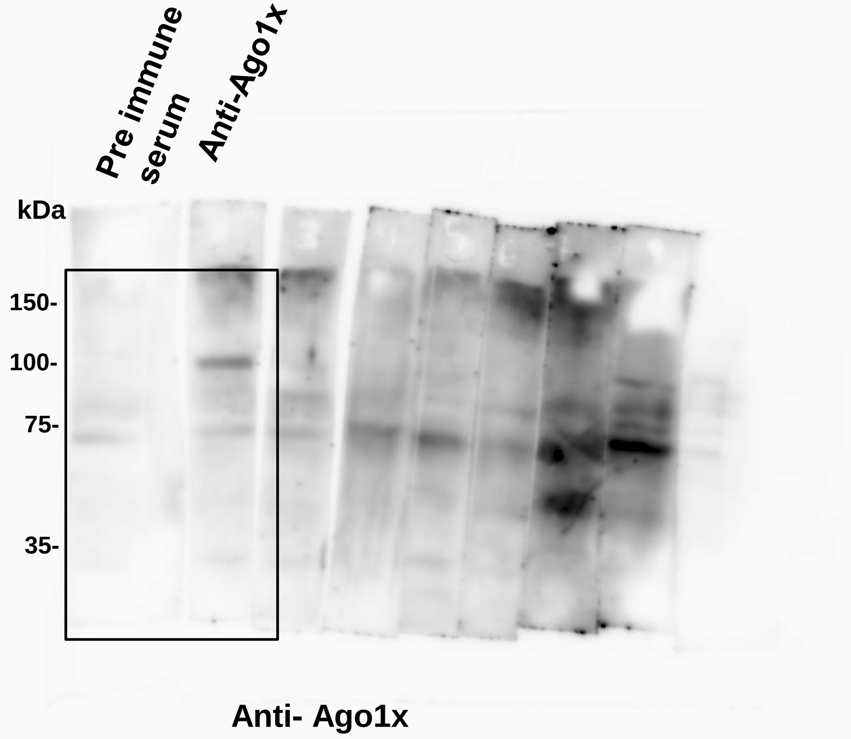

Fig 2 C

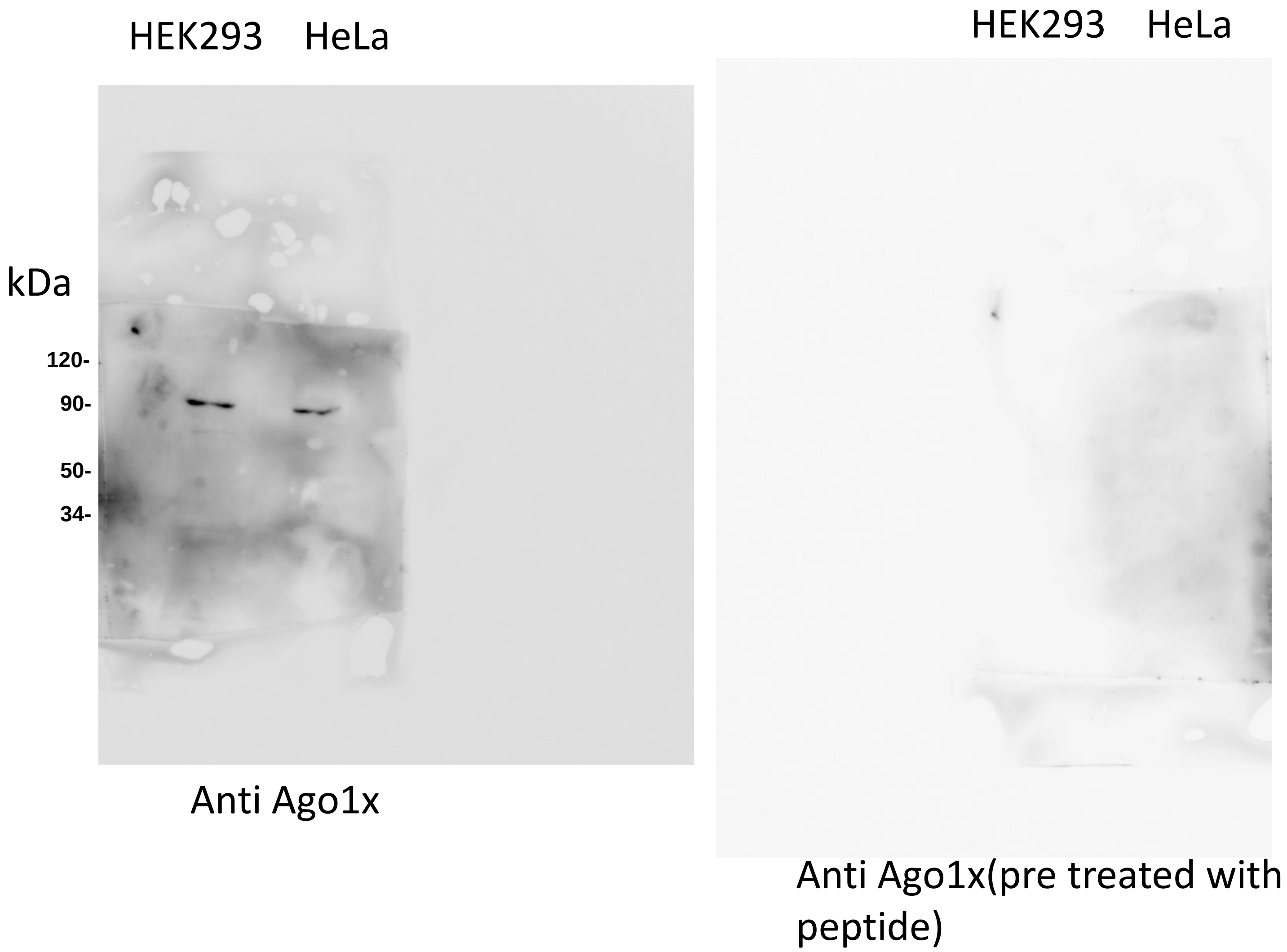
HEK293 HeLa
kDa
120-
90-
50-
34-
Anti Ago1x
HEK293 HeLa
Anti Ago1x(pre treated with peptide)

Fig 2 C

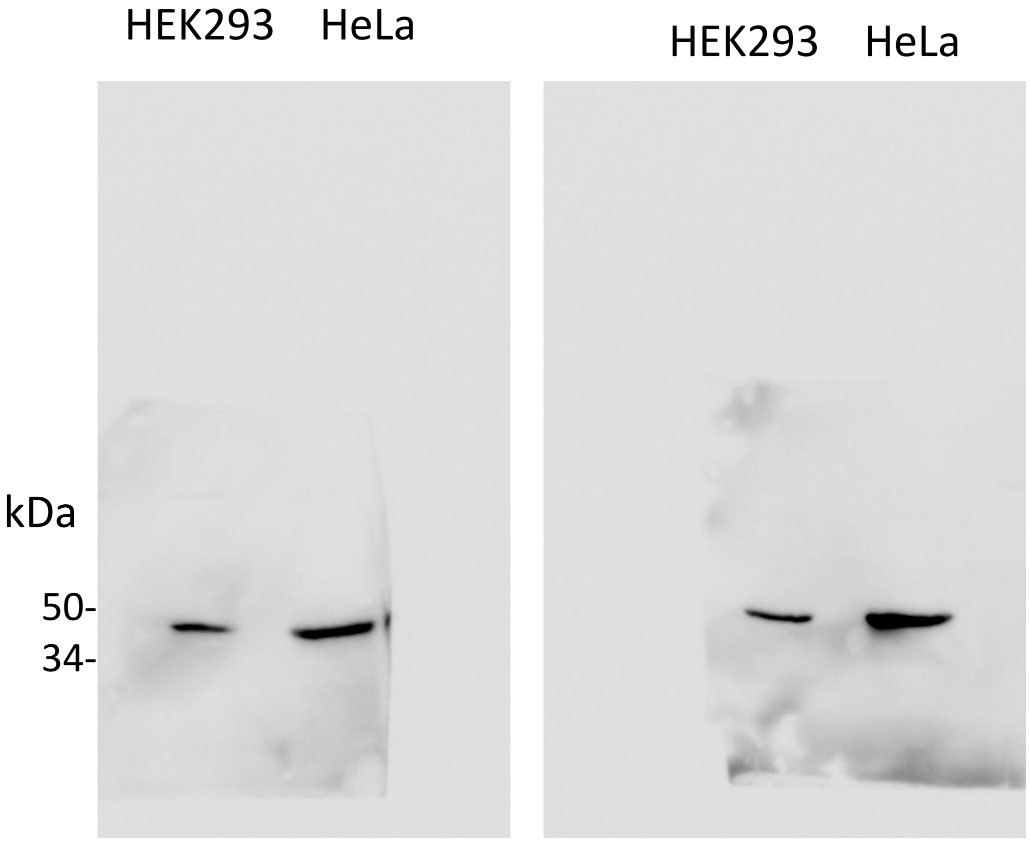

Anti -Actin

Fig 2 D

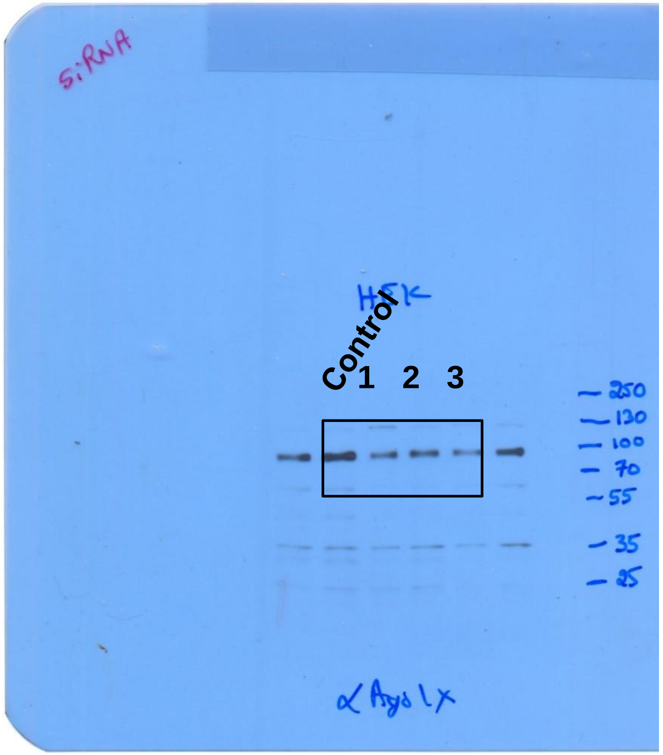

siRNA
HSK
Control
1
2
3
250
130
100
70
55
35
25
α Agd1x

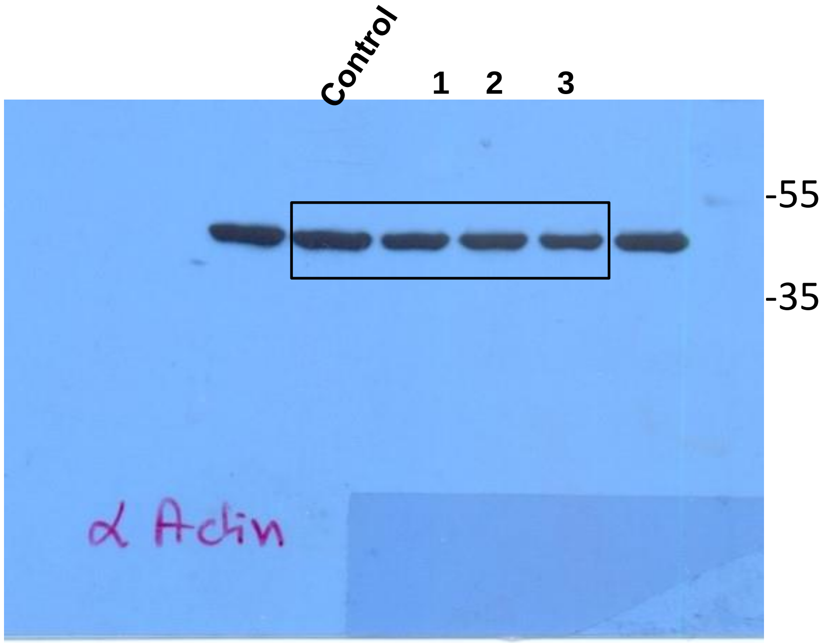

Control
1
2
3
-55
-35
α Actin

Fig 2 E

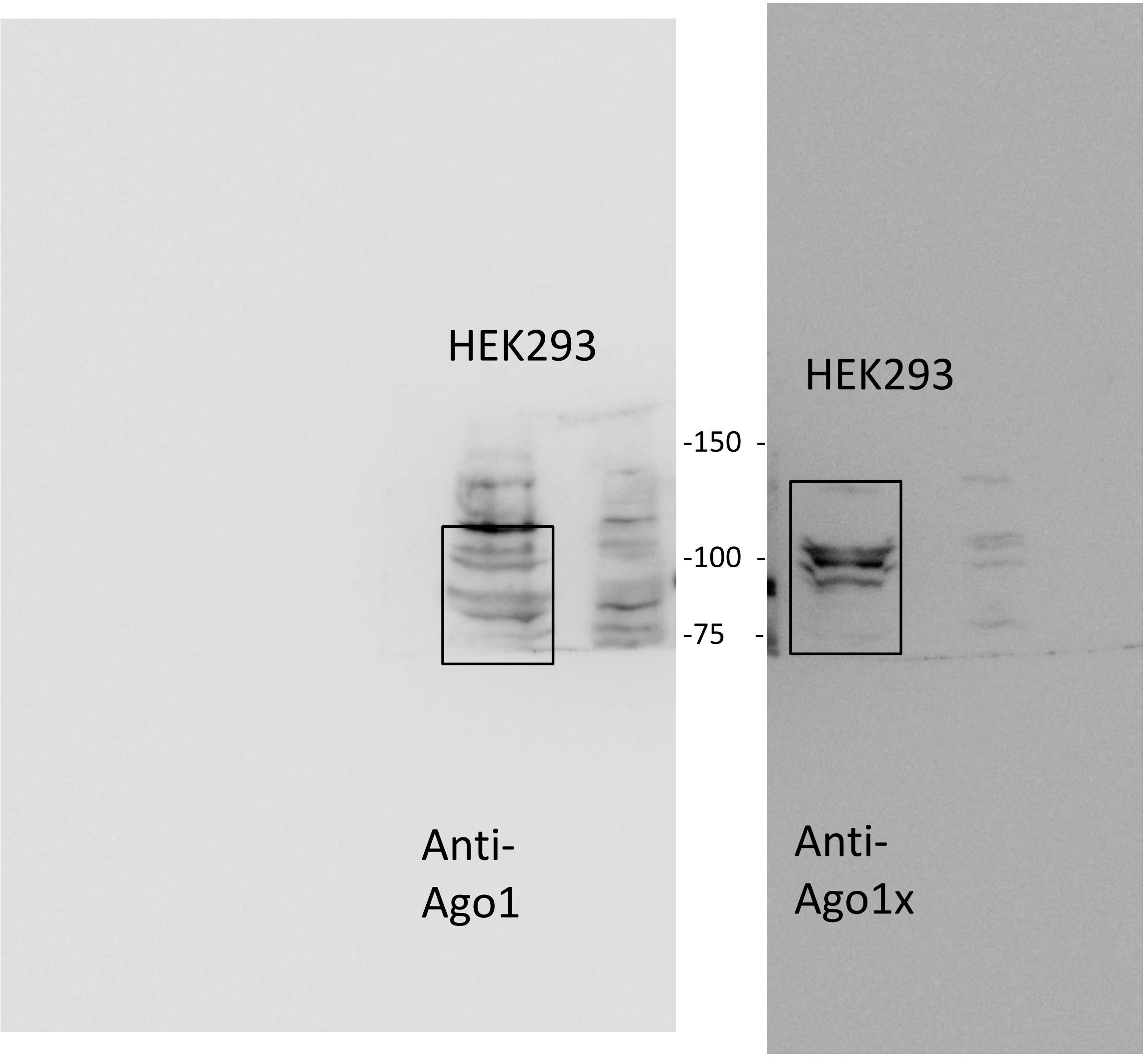

Fig 2 F

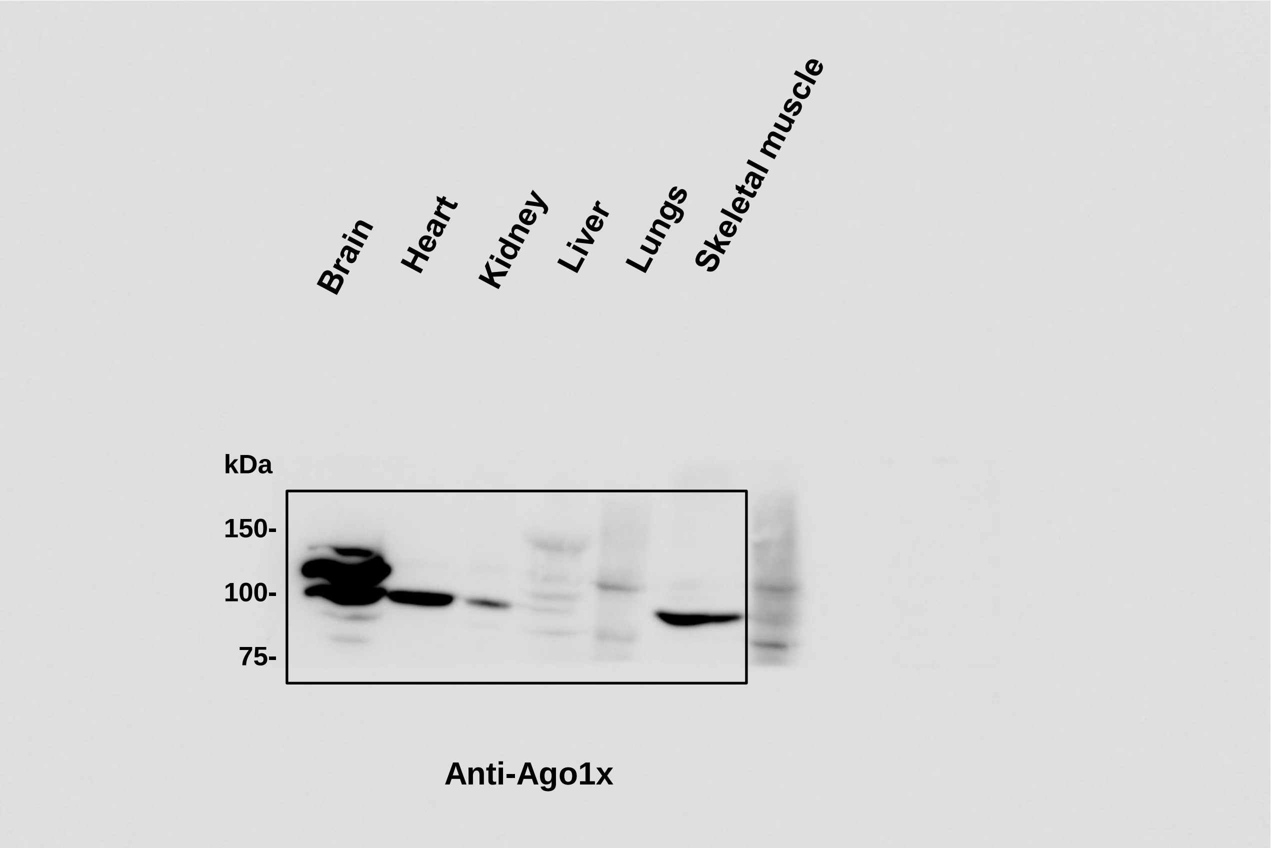

Brain
Heart
Kidney
Liver
Lungs
Skeletal muscle
kDa
150-
100-
75-
Anti-Ago1x

Fig 2 G

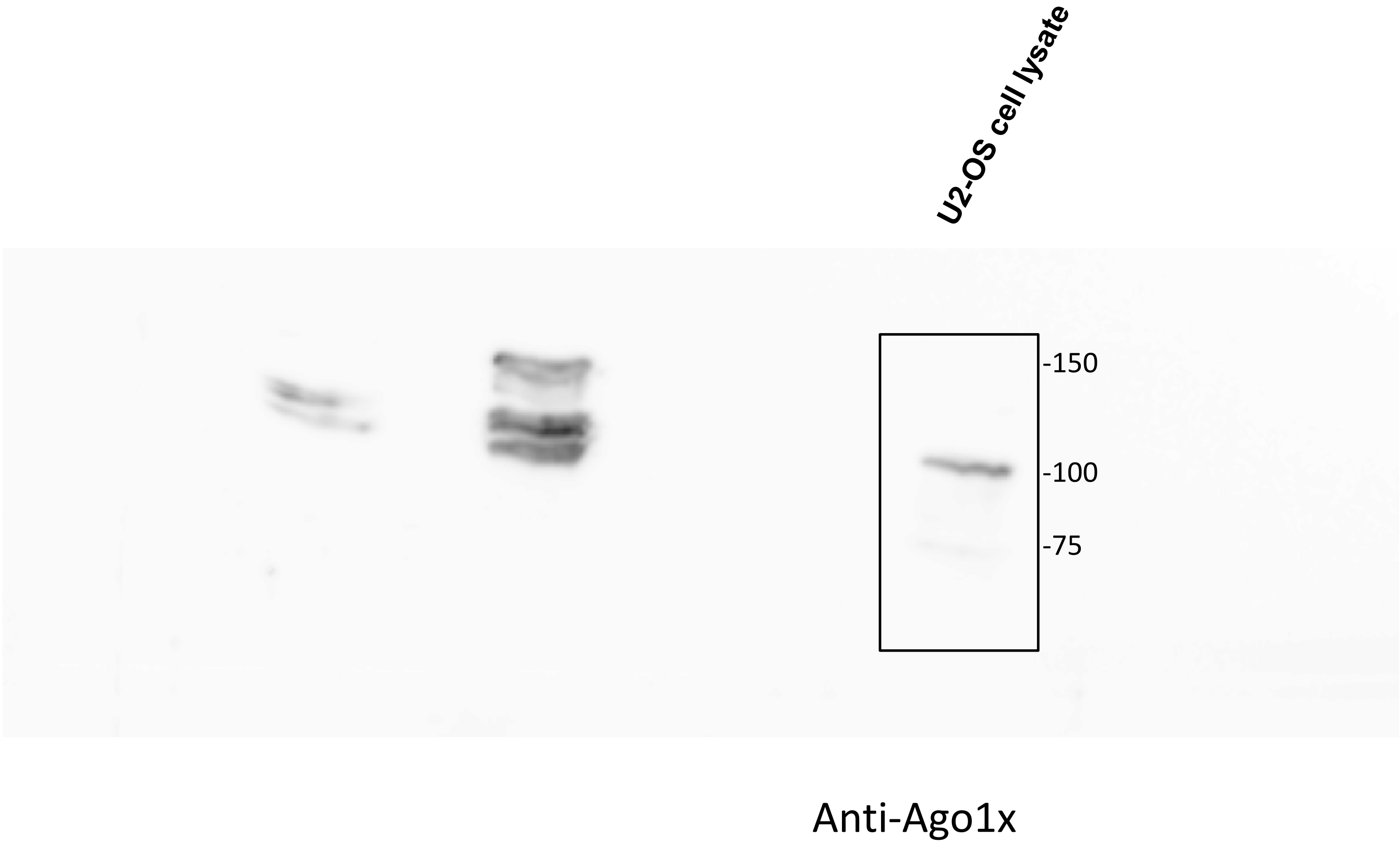

**FIG_2_D**						
**AGO1**						
				average	sem	**P-value:**
**Control**	1	0.9406	1.0992	1.0132	0.0801	<0.0001
**Ago1 siRNA 1**	0.0415	0.0571	0.1068	0.0685	0.0341	
**Ago1 siRNA 2**	0.0491	0.087	0.1139	0.0833	0.0325	
**Ago1 siRNA 3**	0.0358	0.0214	0.0304	0.0292	7.28E-03	

FIG_2_E		
	Ago1	Ago1x
Rep-1	51.3162	48.6838
Rep-2	63.5097	36.4903
Rep-3	68.7994	31.2006
average	61.20843	38.79157
