# Supplementary material for: Let‐7a‐regulated translational readthrough of mammalian AGO1 generates a microRNA pathway inhibitor
Source: EMBO J. 2019 Jul 22;38(16):e100727. doi: 10.15252/embj.2018100727 (PMC6694283; doi:10.15252/embj.2018100727)
Supplement: Supplementary file 9 — Source Data for Figure 3 [file EMBJ-38-e100727-s007.pdf]

Fig 3B

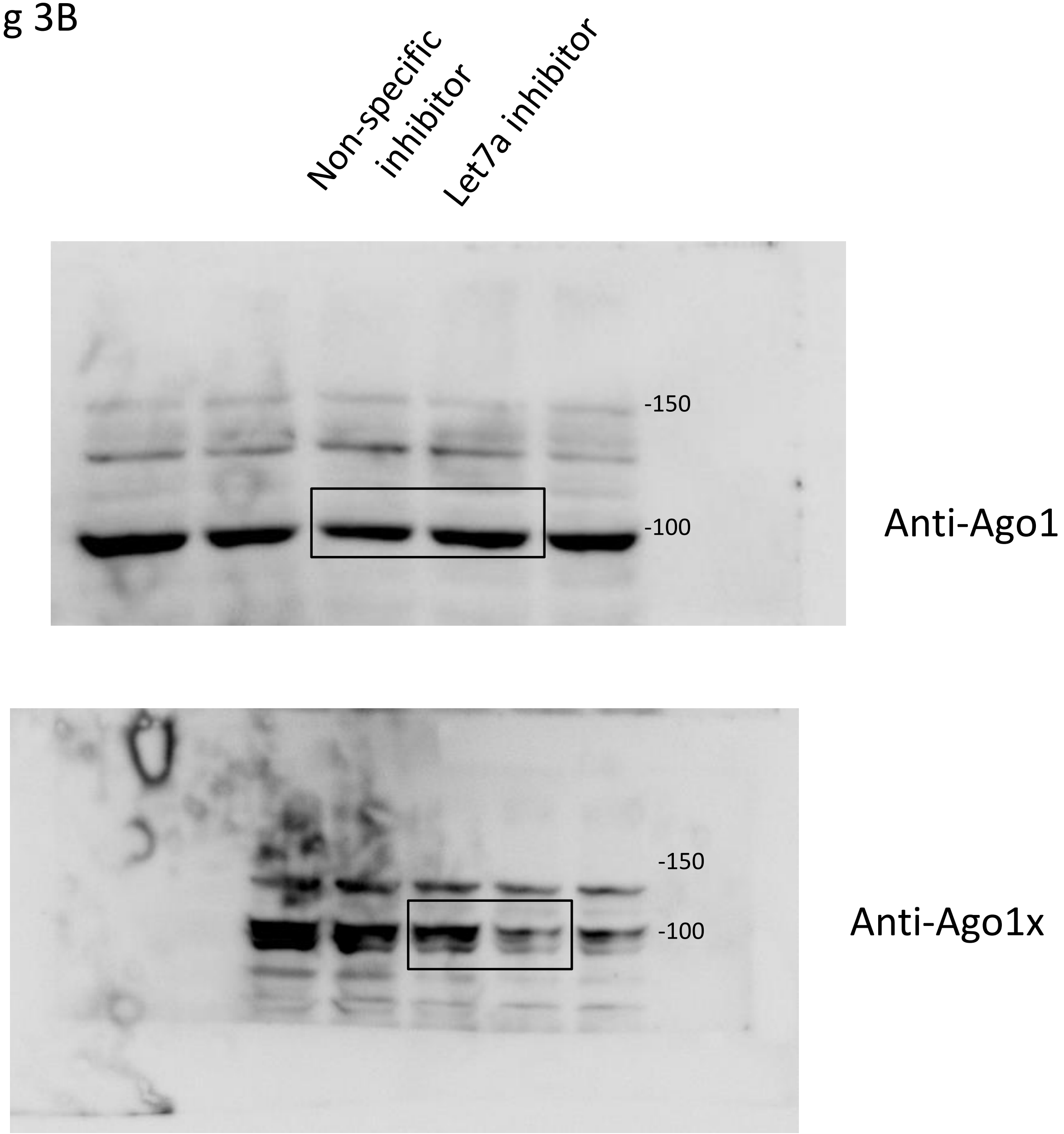
Non-specific inhibitor
Let7a inhibitor
-150
-100
Anti-Ago1
-150
-100
Anti-Ago1x

Fig 3C

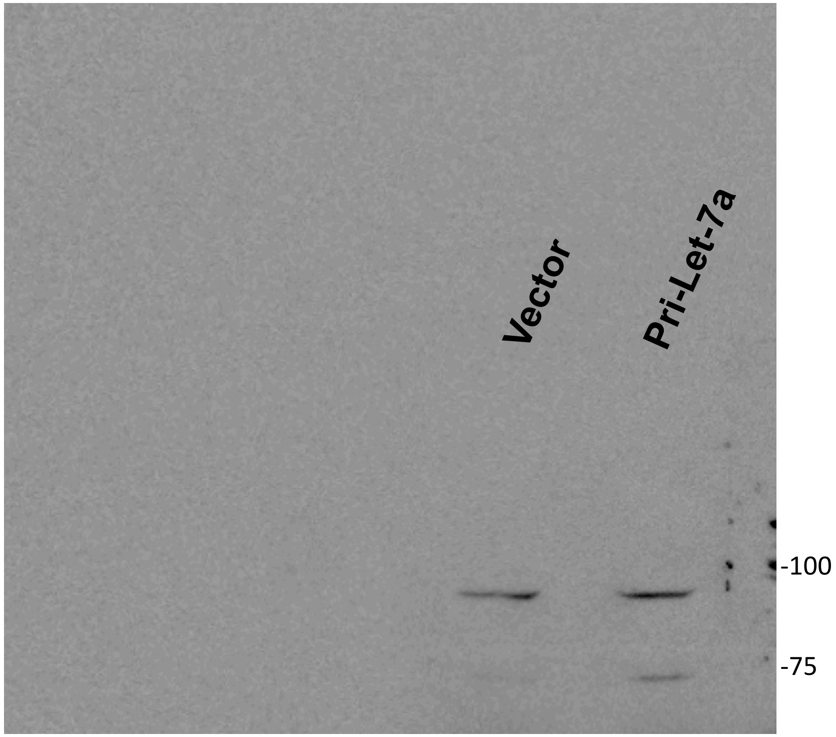

Anti-Ago1

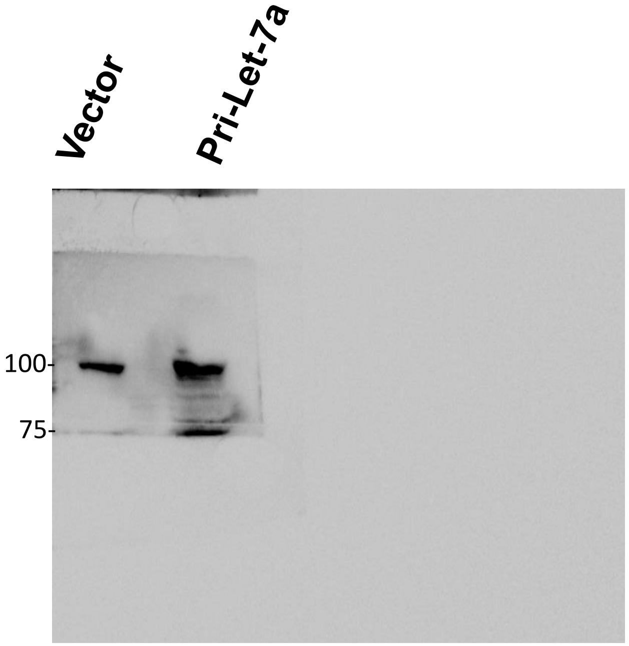

Anti-Ago1x

Fig 3D

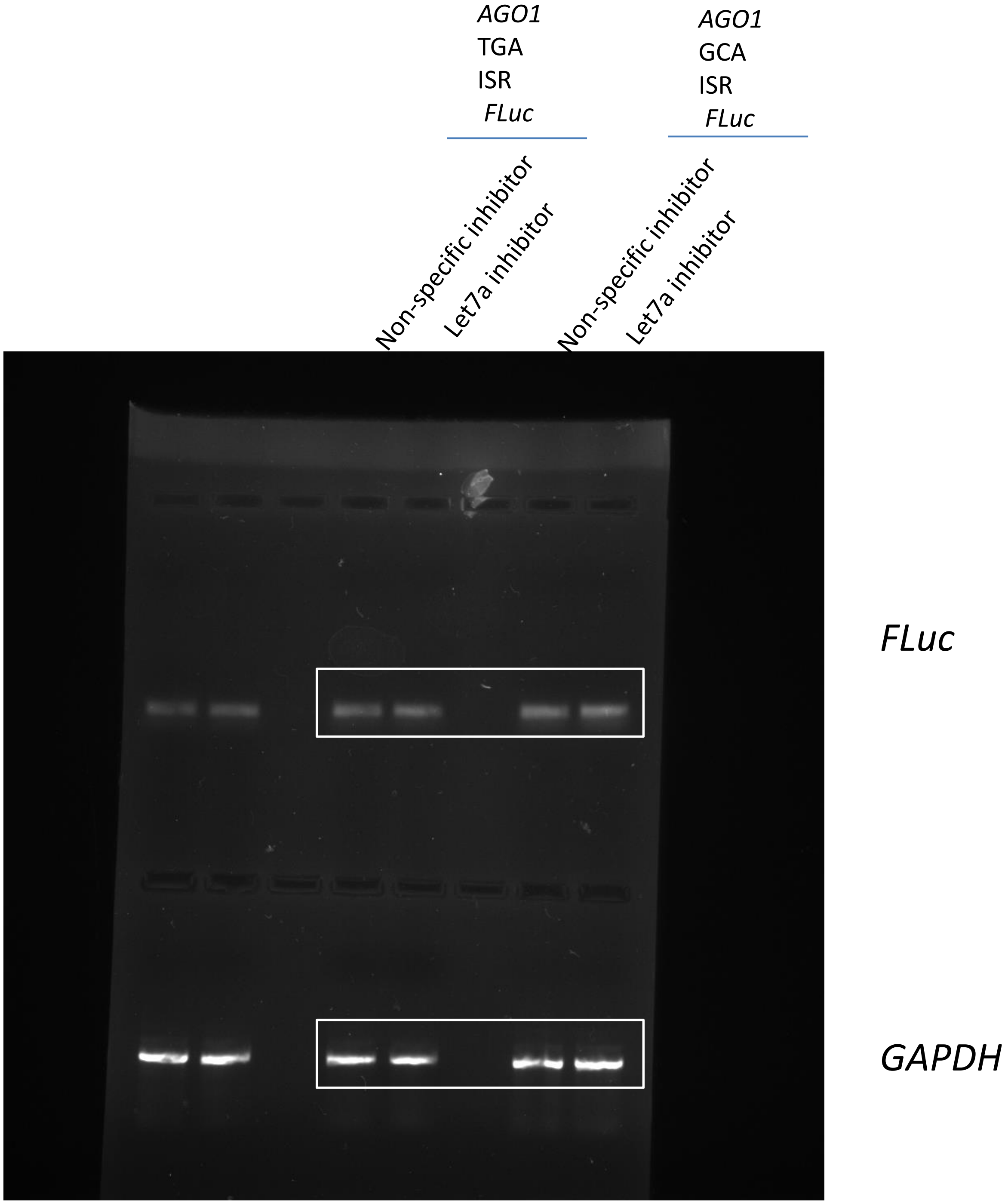

AGO1
TGA
ISR
FLuc
AGO1
GCA
ISR
FLuc
Non-specific inhibitor
Let7a inhibitor
Non-specific inhibitor
Let7a inhibitor
FLuc
GAPDH

Fig 3E

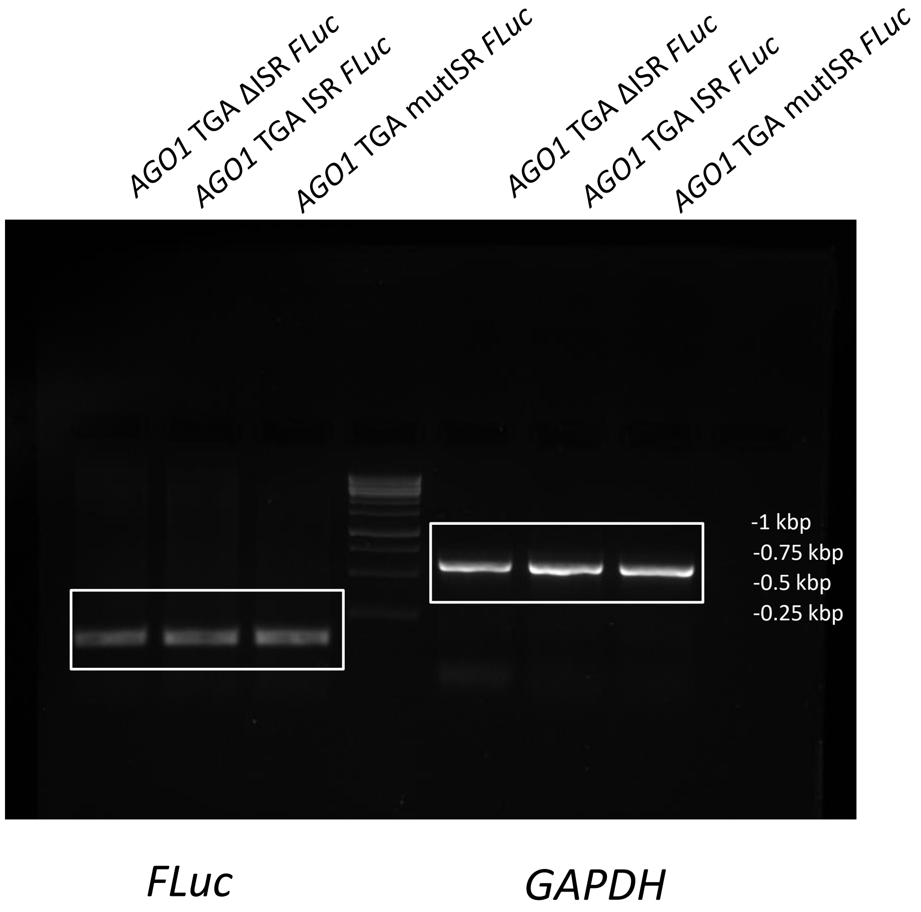
AGO1 TGA ΔISR FLuc
AGO1 TGA ISR FLuc
AGO1 TGA mutISR FLuc
AGO1 TGA ΔISR FLuc
AGO1 TGA ISR FLuc
AGO1 TGA mutISR FLuc
-1 kbp
-0.75 kbp
-0.5 kbp
-0.25 kbp
FLuc
GAPDH

Fig 3F

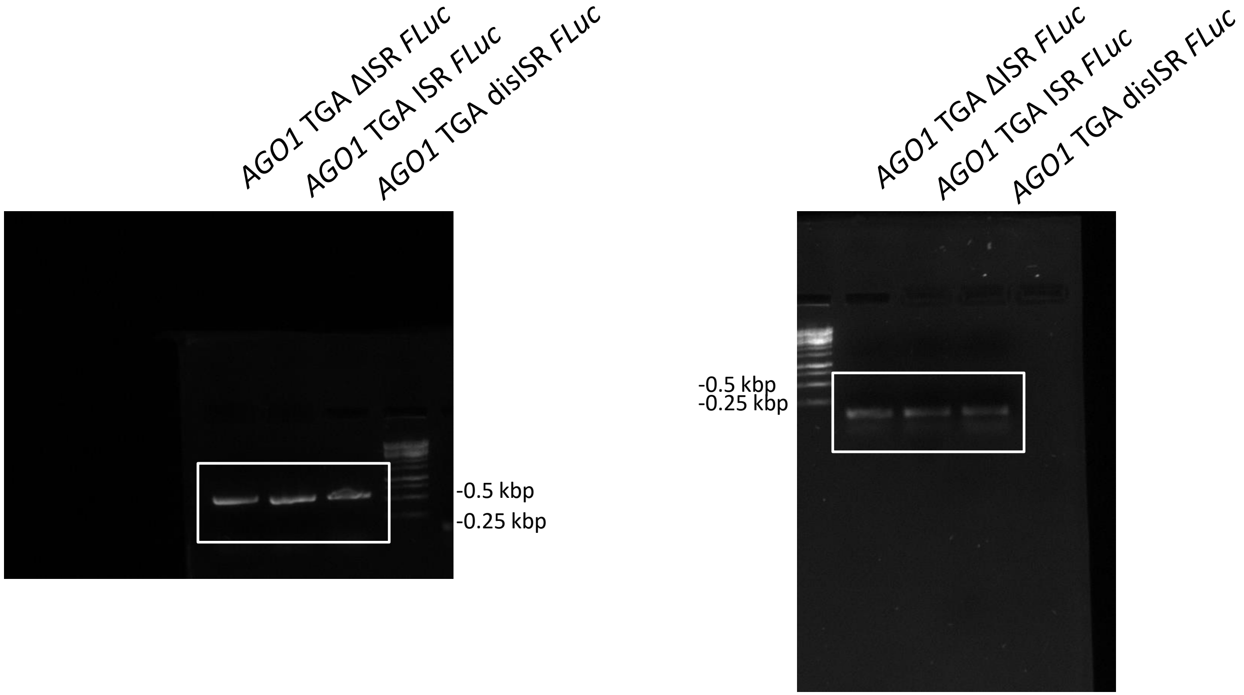

GAPDH

FLuc

FIG_3_B

	Normalised Intensity				
	Rep-1	Rep-2	Rep-3	Average	SEM
AGO1 with LET-7a Inhibitor	1.044109	1.437819	0.670709	1.050879	0.2215
AGO1 with Non-Specific Inhibitor	1	1	1	1	0
AGO1x with LET-7a Inhibitor	0.347928	0.482894	0.527353	0.452725	0.0539
AGO1x Non-Specific Inhibitor	1	1	1	1	0
				P-value:	0.01

FIG_3_C						
		Normalised Intensity				
		Rep-1	Rep-2	Rep-3	Average	SEM
Ago1	Vector	1	1	1	1	0
	Let-7a over-exp	0.430604	1.333301193	1.407001	1.057	0.3139
Ago1x	Vector	1	1	1	1	0
	Let-7a over-exp	2.283398	2.535463914	2.713007	2.5106	0.1246
					P-value:	0.007

FIG_3_D					
Let-7a inh. 10 nm					
		Fluc/Rluc	Average	SEM	
AGO1 TGA ISR Fluc		0.000201	0.000583	3.39E-04	
		0.000288			
		0.001258			
AGO1 GCA ISR Fluc		0.003091	0.013113	6.77E-03	
		0.010229			
		0.026018			
					P-value:
					0.027
non-specific inh. 10nm					
AGO1 TGA ISR Fluc		0.001905	0.002458	4.35E-04	
		0.002153			
		0.003315			
AGO1 GCA ISR Fluc		0.010621	0.011665	6.13E-04	
		0.011631			
		0.012744			

**FIG_3_E**							
		**Fluc/Rluc**	**Average**	**SEM**			
**AGO1 TGA Delta ISR Fluc**		2.77941E-05	2.99675E-05	1.49E-06			
		3.28313E-05					
		2.92771E-05					
**AGO1 TGA ISR Fluc**		0.00044249	0.000615335	8.67E-05			
		0.000713433					
		0.000690083					
**AGO1 TGA Mut. ISR Fluc**		0.000175624	0.000182688	9.82E-06		**P-value:**	0.008
		0.000170343					
		0.000202096					

FIG_3_F							
		Fluc/Rluc	Average	SEM			
AGO1 TGA Delta ISR Fluc		6.93E-05	7.05534E-05	6.49E-07			
		7.14E-05					
		7.09E-05					
AGO1 TGA ISR Fluc		0.000977	0.000902616	3.97E-05			
		0.000888					
		0.000843					
AGO1 TGA disISR Fluc		0.000269	0.000262889	5.79E-06		P-value:	< 0.0001
		0.000251					
		0.000268					
