# Supplementary material for: Let‐7a‐regulated translational readthrough of mammalian AGO1 generates a microRNA pathway inhibitor
Source: EMBO J. 2019 Jul 22;38(16):e100727. doi: 10.15252/embj.2018100727 (PMC6694283; doi:10.15252/embj.2018100727)
Supplement: Supplementary file 10 — Source Data for Figure 4 [file EMBJ-38-e100727-s008.pdf]

Fig 4 A

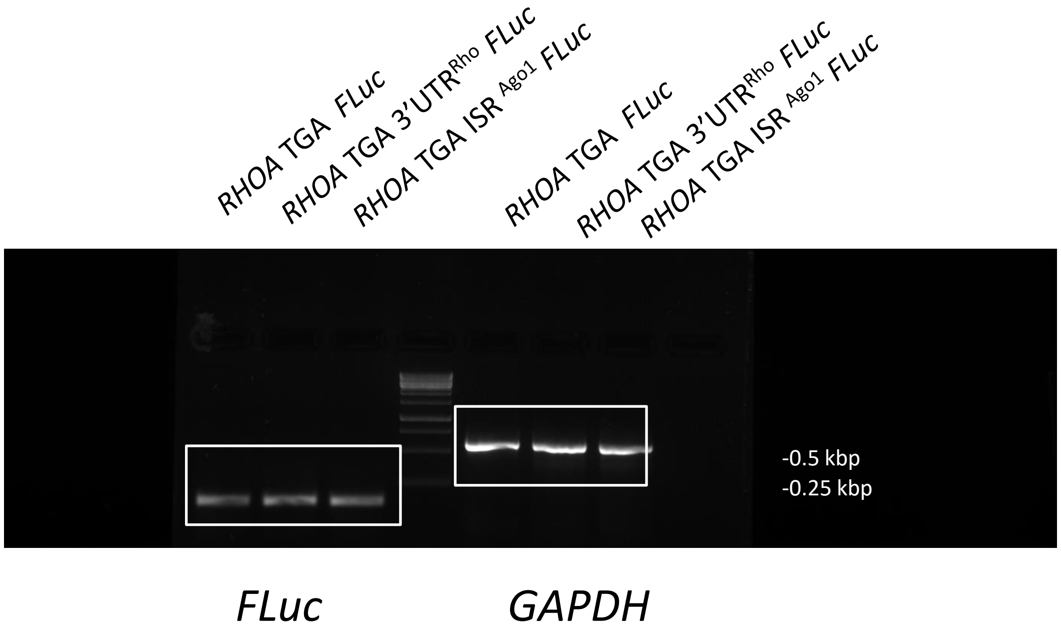
RHOA TGA FLuc
RHOA TGA 3'UTR Rho FLuc
RHOA TGA ISR Ago1 FLuc
RHOA TGA FLuc
RHOA TGA 3'UTR Rho FLuc
RHOA TGA ISR Ago1 FLuc
-0.5 kbp
-0.25 kbp
FLuc
GAPDH

Fig 4 C

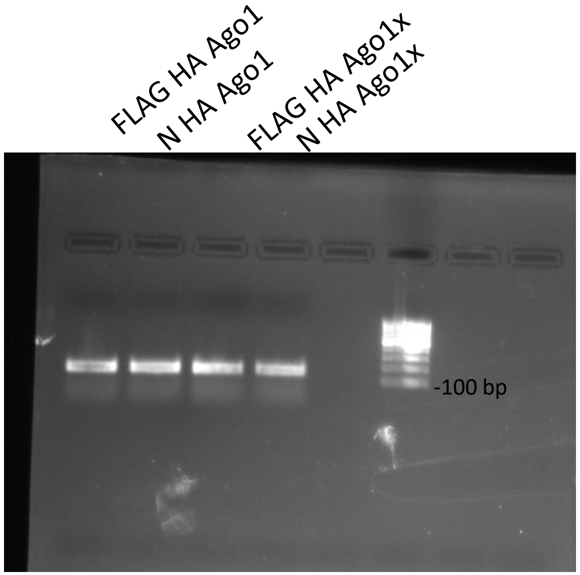


*FLuc*

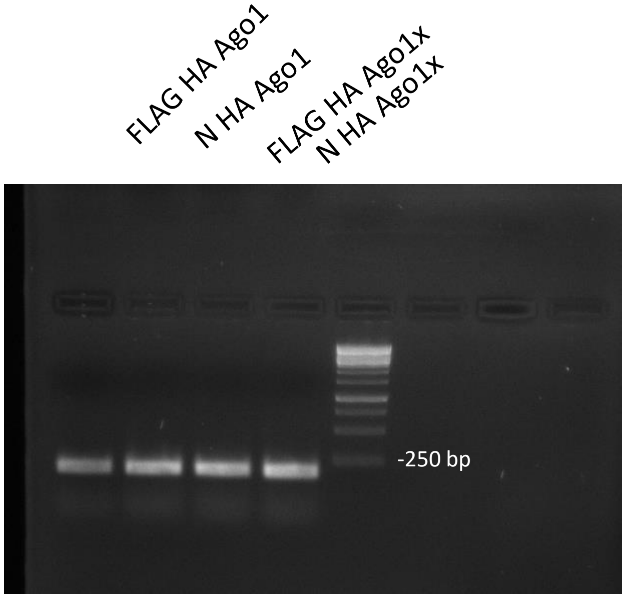


*Actin*

| | | | | | | |
|---|---|---|---|---|---|---|
| **FIG_4_A** | | | | | | |
| | | | | | | |
| | **Fluc/Rluc** | **Average** | **SEM** | | | |
| **RHOA TGA Fluc** | 3.67688E-05 | 3.40642E-05 | 8.18E-06 | | | |
| | 1.87463E-05 | | | | | |
| | 4.66775E-05 | | | | | |
| **RHOA TGA 3'UTR RHO Fluc** | 3.19249E-05 | 4.20164E-05 | 1.09E-05 | | | |
| | 3.03944E-05 | | | | | |
| | 6.37298E-05 | | | | | |
| **RHOA TGA ISR AGO1 Fluc** | 0.000258907 | 0.000254826 | 2.79E-06 | | **P-value:** | <0.0001 |
| | 0.000256071 | | | | | |
| | 0.000249501 | | | | | |
| | | | | | | |

| | | | | | | | |
|---|---|---|---|---|---|---|---|
| FIG_4_B | | | | | | | |
| in vitro translation | | | | | | | |
| | | Fluc/Rluc | Average | SEM | | | |
| Rluc TAA non-specific Fluc | | 0.001355 | 0.001301 | 3.40E-05 | | | |
| | | 0.001308 | | | | | |
| | | 0.001238 | | | | | |
| Rluc TAA ISR Fluc | | 0.008087 | 0.007289 | 4.14E-04 | | P-value: | 0.0001 |
| | | 0.007081 | | | | | |
| | | 0.0067 | | | | | |
| Rluc TCA ISR Fluc | | 0.084791 | 0.082046 | 6.17E-03 | | | |
| | | 0.070251 | | | | | |
| | | 0.091096 | | | | | |
| transfection | | | | | | | |
| | | Fluc/Rluc | Average | SEM | | | |
| Rluc TAA non-specific Fluc | | 0.0044 | 0.004848 | 8.06E-04 | | | |
| | | 0.006412 | | | | | |
| | | 0.003731 | | | | | |
| Rluc TAA ISR Fluc | | 0.011739 | 0.013515 | 1.39E-03 | | | |
| | | 0.01625 | | | | P-value: | 0.006 |
| | | 0.012556 | | | | | |
| Rluc TCA ISR Fluc | | 0.209804 | 0.200688 | 0.0181 | | | |
| | | 0.226506 | | | | | |
| | | 0.165753 | | | | | |

| | | | | | | |
|---|---|---|---|---|---|---|
| FIG_4_C | | | | | | |
| | | | | | | |
| | Fluc/Rluc | Average | SEM | | | |
| nha ago1 | 0.00092 | 0.000843 | 1.19E-04 | | | |
| | 0.001 | | | | | |
| | 0.000609 | | | | | |
| | | | | | | |
| | | | | | | |
| | Fluc/Rluc | Average | SEM | | | |
| nha ago1x | 0.000506 | 0.000389 | 6.49E-05 | | | |
| | 0.00038 | | | | | |
| | 0.000282 | | | | | |
| | | | | | | |
| | | | | | | |
| | Fluc/Rluc | Average | SEM | | | |
| flag ha ago1 | 0.000347 | 0.00036 | 1.20E-05 | | | |
| | 0.000384 | | | P-value: | 0.016 | |
| | 0.000348 | | | | | |
| | | | | | | |
| | | | | | | |
| | Fluc/Rluc | Average | SEM | | | |
| fha ago1x | 4.77E-05 | 5.98E-05 | 6.25E-06 | | | |
| | 6.85E-05 | | | P-value: | 0.0355 | |
| | 6.33E-05 | | | | with welch correction | |
| | | | | | | |
