# Supplementary material for: Let‐7a‐regulated translational readthrough of mammalian AGO1 generates a microRNA pathway inhibitor
Source: EMBO J. 2019 Jul 22;38(16):e100727. doi: 10.15252/embj.2018100727 (PMC6694283; doi:10.15252/embj.2018100727)
Supplement: Supplementary file 12 — Source Data for Figure 6 [file EMBJ-38-e100727-s010.pdf]

Fig 6 A

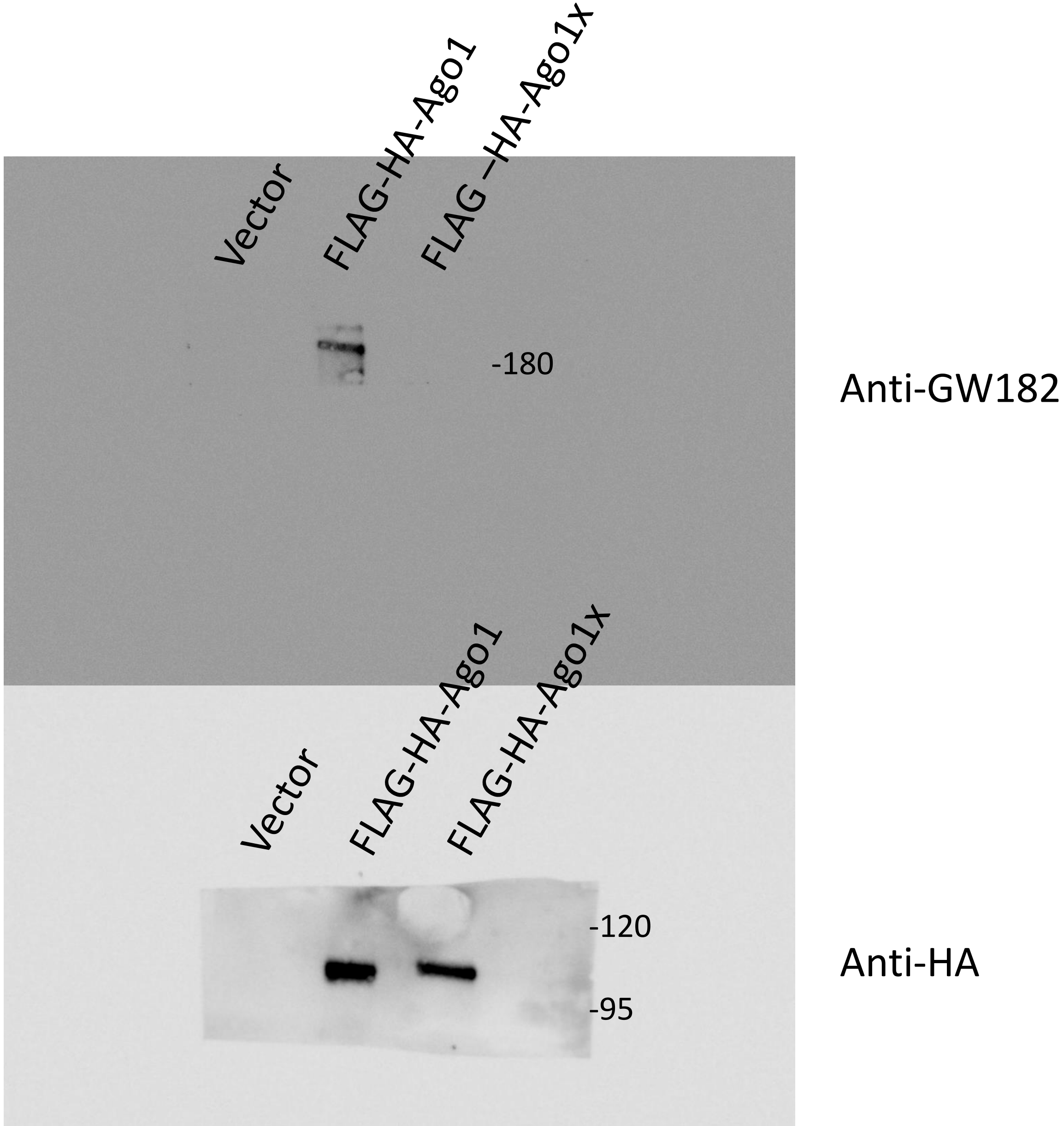
Vector
FLAG-HA-Ago1
FLAG –HA-Ago1x
-180
Anti-GW182
Vector
FLAG-HA-Ago1
FLAG-HA-Ago1x
-120
-95
Anti-HA

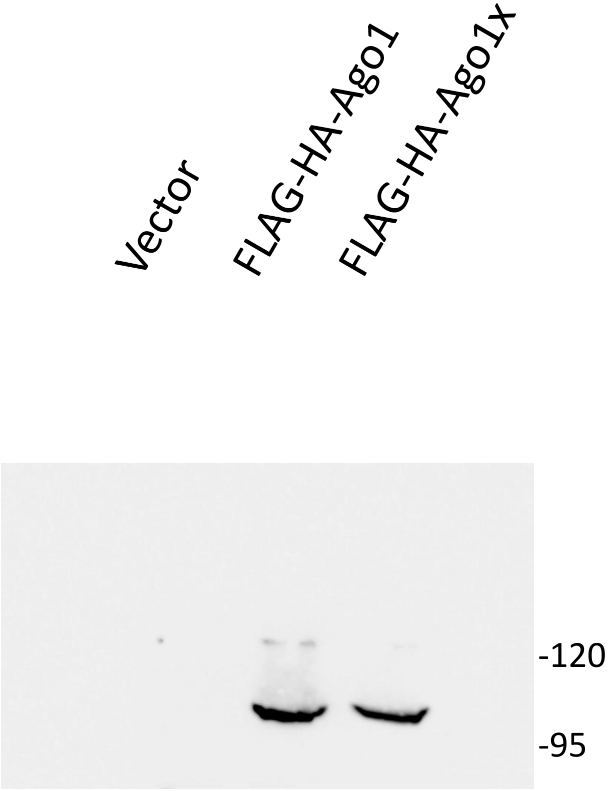

Anti-HA

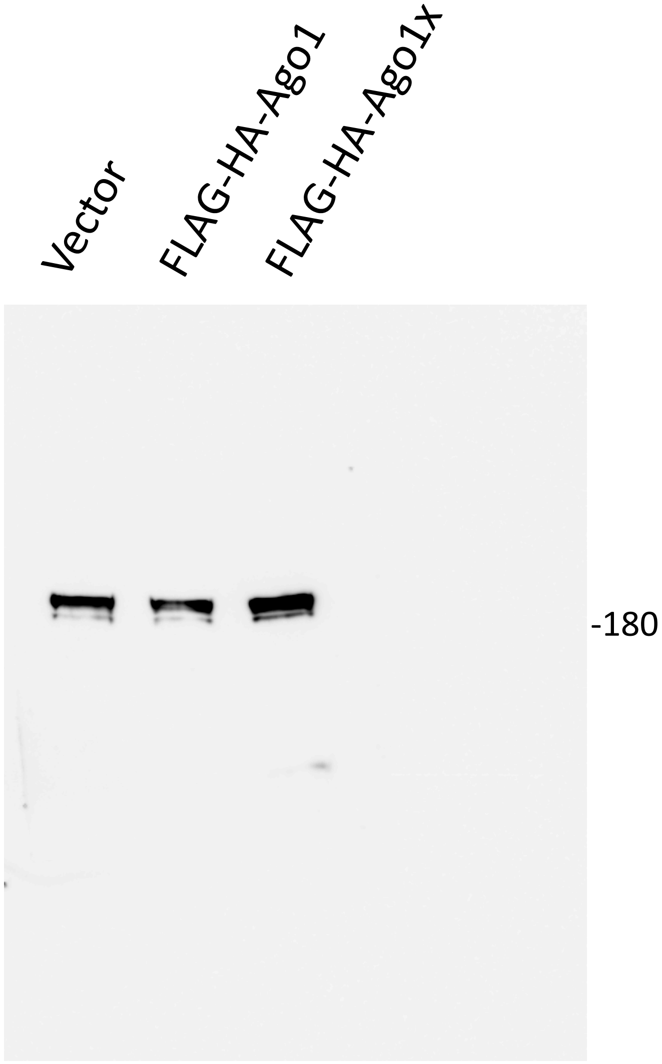

Anti-GW182

FIG_6_C													
		Ago1					Ago1x					Vector	
Microcopic field	no. of cells	no. of particles	particles/cell		Microcopic field	no. of cells	no. of particles	particles/cell		Microcopic fi	no. of cells	no. of particles	particles/cell
1	19	904	47.57894737		1	7	0	0		1	66	4	0.060606061
2	11	448	40.72727273		2	5	3	0.6		2	40	5	0.125
3	6	145	24.16666667		3	15	6	0.4		3	24	3	0.125
4	24	876	36.5		4	16	32	2		4	21	2	0.095238095
5	10	60	6		5	17	36	2.117647059		5	22	6	0.272727273
6	6	30	5		6	18	7	0.388888889		6	18	5	0.277777778
7	5	39	7.8		7	10	1	0.1		7	17	1	0.058823529
8	6	57	9.5		8	19	8	0.421052632		8	17	2	0.117647059
9	5	72	14.4		9	12	1	0.083333333		9	26	2	0.076923077
10	4	151	37.75		10	15	1	0.066666667		10	28	4	0.142857143
11	5	51	10.2		11	10	0	0					
12	19	65	3.421052632										
	average	sem		p value									
ago1	20.2537	4.6681		<0.0001	mann whitney test								
ago1x	0.5616	0.2313											
vector	0.1353	0.025											
