# Supplementary material for: Let‐7a‐regulated translational readthrough of mammalian AGO1 generates a microRNA pathway inhibitor
Source: EMBO J. 2019 Jul 22;38(16):e100727. doi: 10.15252/embj.2018100727 (PMC6694283; doi:10.15252/embj.2018100727)
Supplement: Supplementary file 13 — Source Data for Figure 7 [file EMBJ-38-e100727-s011.pdf]

Fig 7B

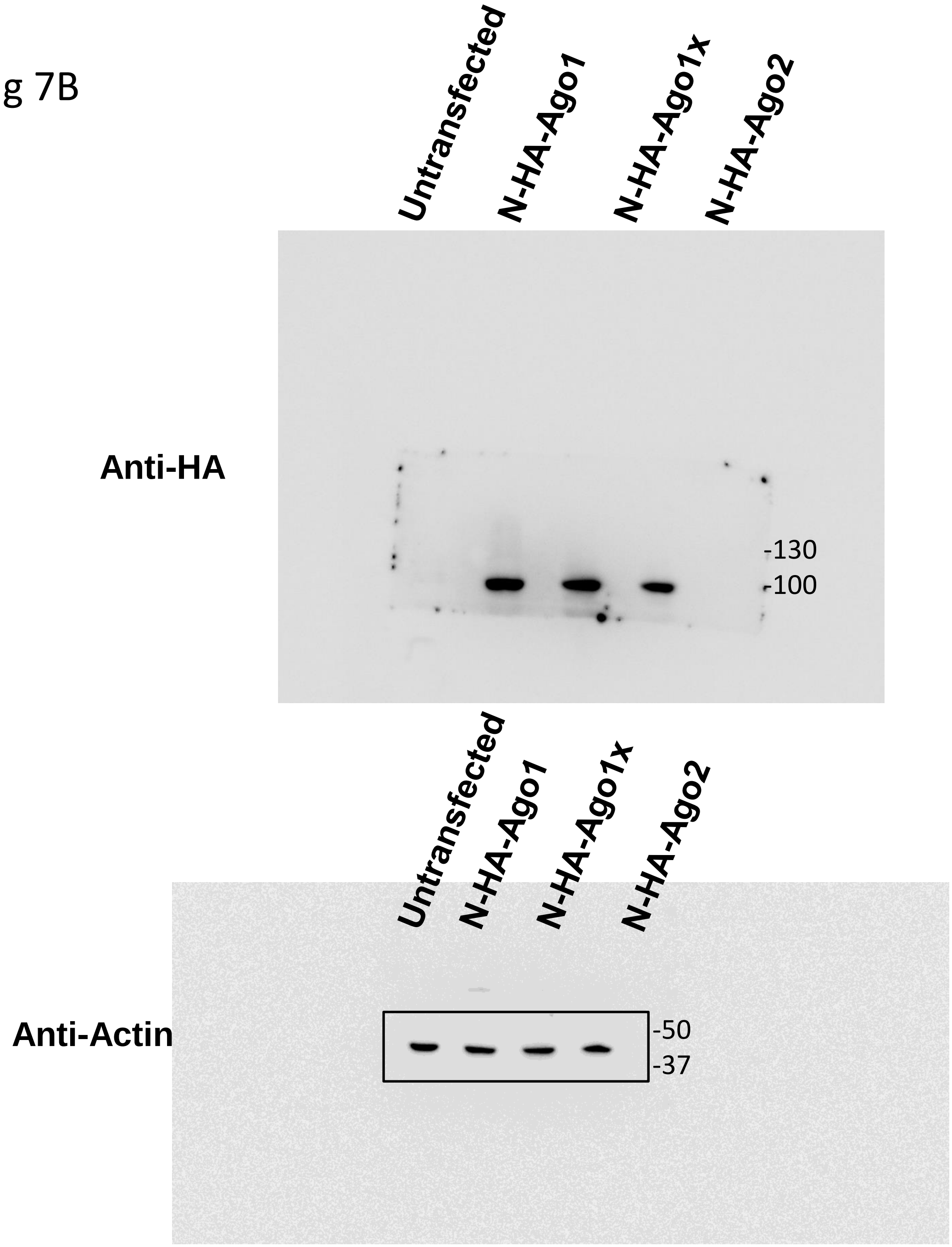
Untransfected
N-HA-Ago1
N-HA-Ago1x
N-HA-Ago2
Anti-HA
-130
-100
Untransfected
N-HA-Ago1
N-HA-Ago1x
N-HA-Ago2
Anti-Actin
-50
-37

Fig 7C

Anti-HA

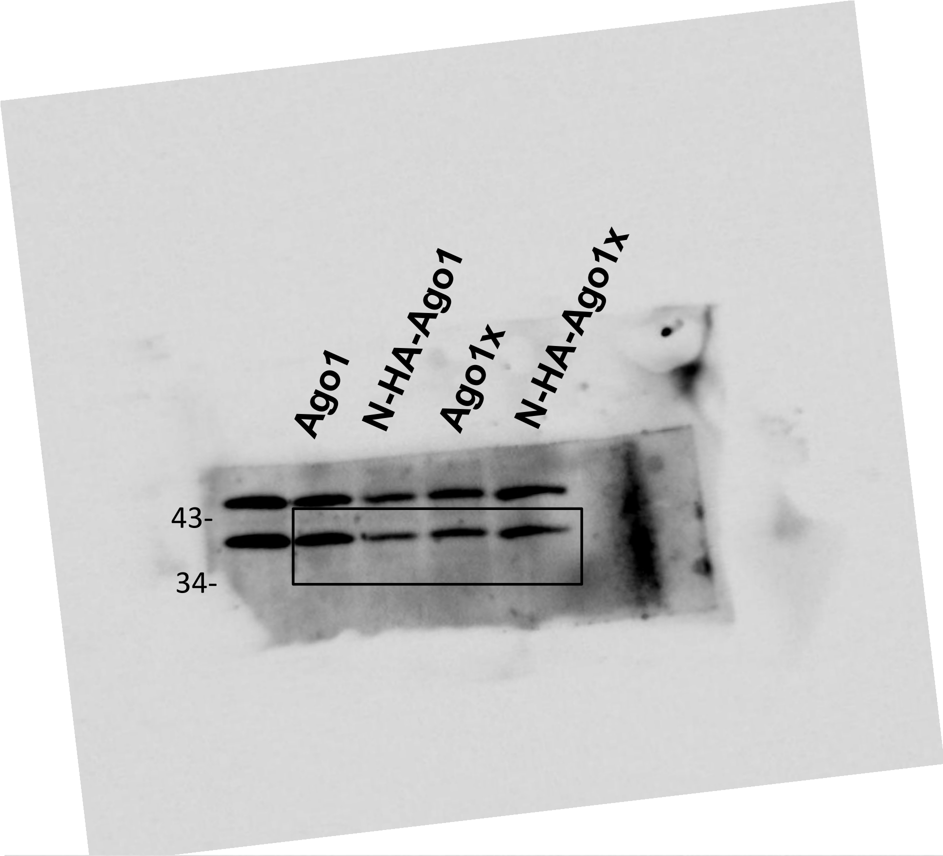

Anti-Actin

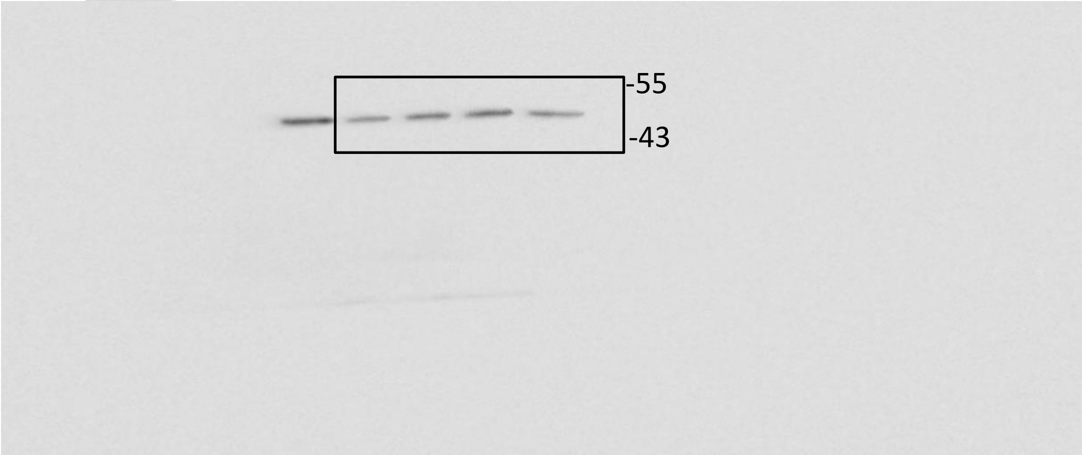

Fig 7C

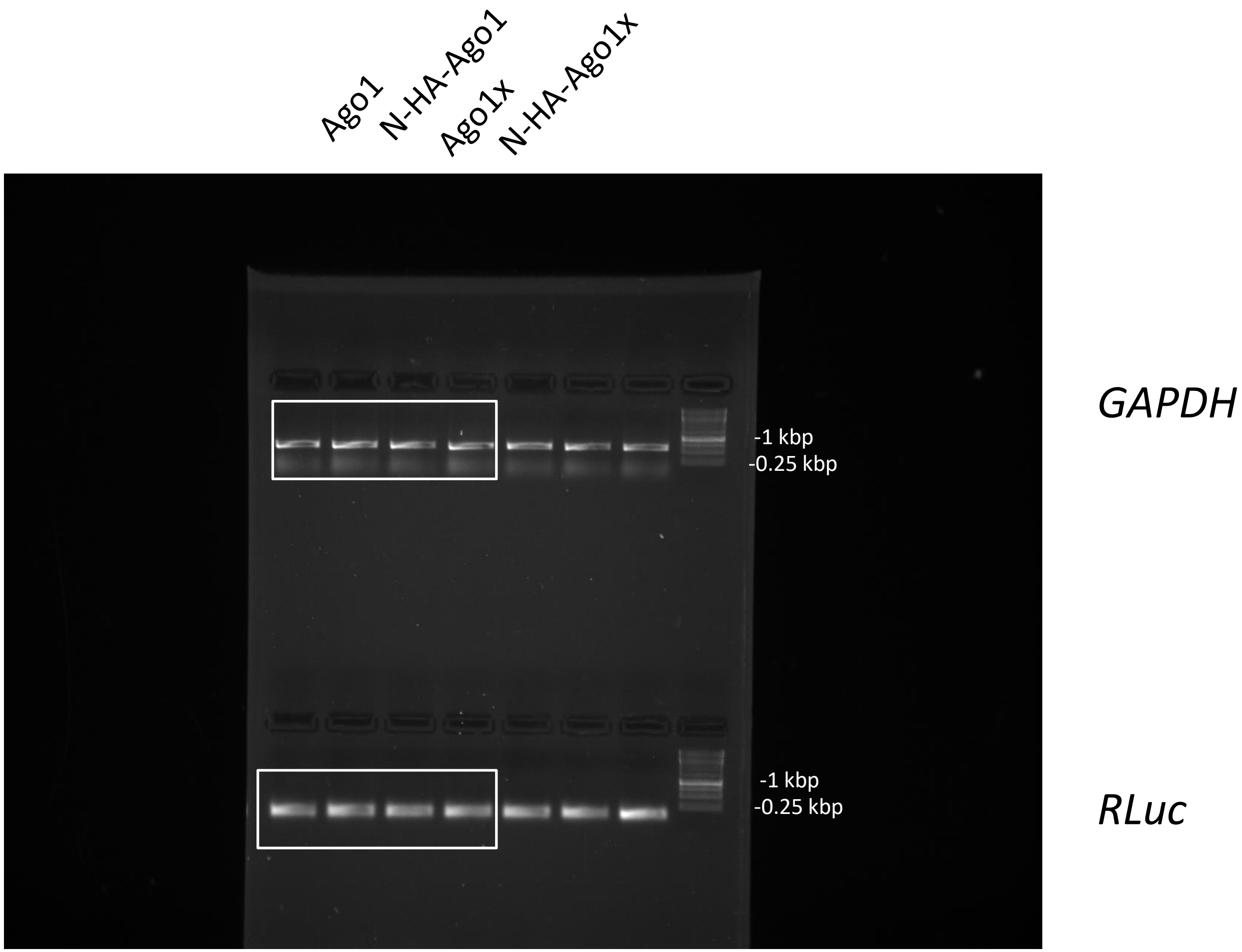
Ago1
N-HA-Ago1
Ago1x
N-HA-Ago1x
-1 kbp
-0.25 kbp
-1 kbp
-0.25 kbp
GAPDH
RLuc

Fig 7D

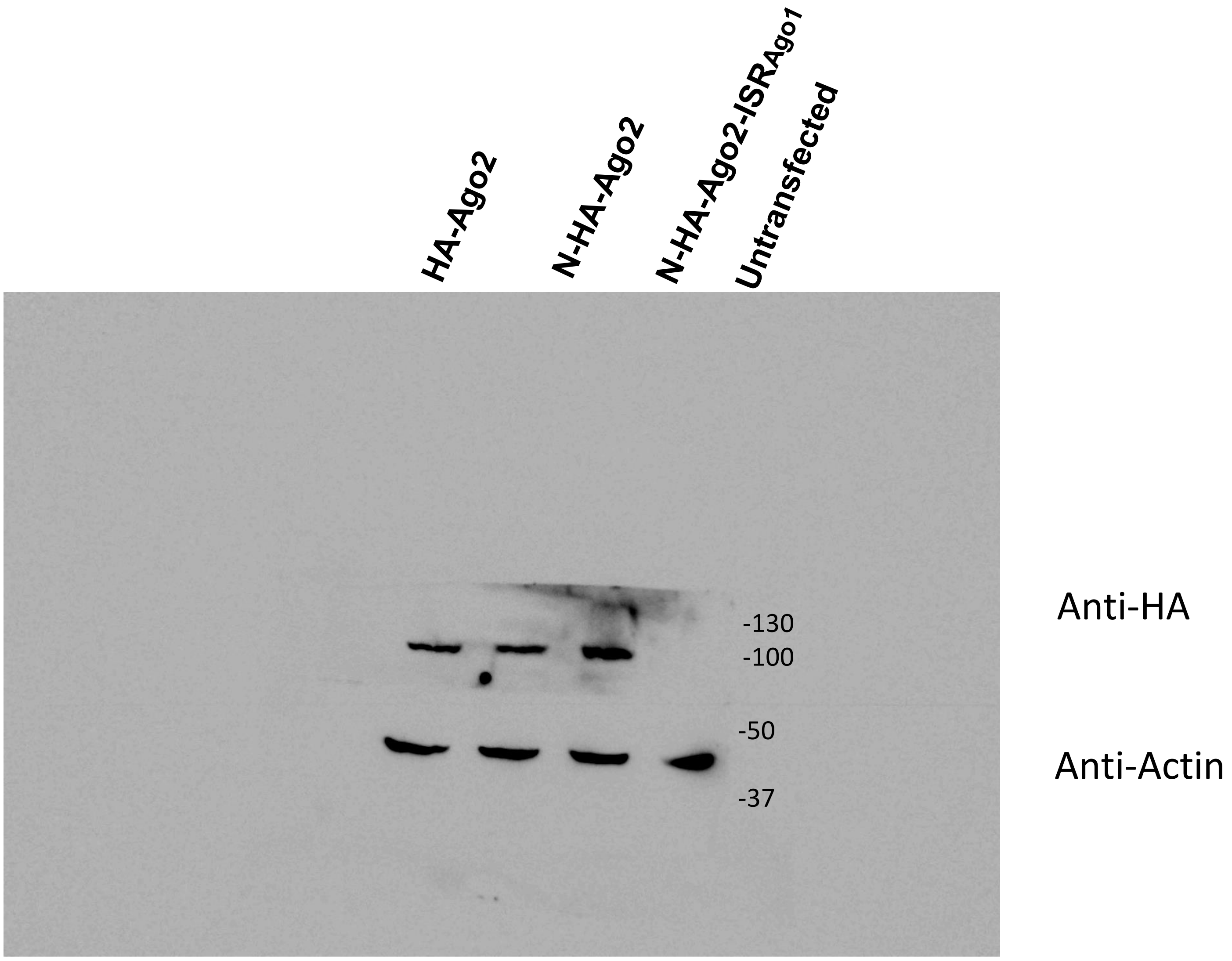
HA-Ago2
N-HA-Ago2
N-HA-Ago2-ISRAgo1
Untransfected
-130
-100
-50
-37
Anti-HA
Anti-Actin

FIG_7_B								
	Rluc/Fluc	Average	SEM					
NHA AGO2	28553.3	36083.63	6556.279					
	30552.29							
	49145.3						P-value:	
HA AGO2	68479.36	65112.59	1891.05				AGO2	0.013
	61936.94						AGO1	0.018
	64921.47						AGO1x	0.041
NHA AGO1X	55059.52	51226.14	2348.663					
	51660.52							
	46958.38							
AGO1X	30000	37750.67	3876.088					
	41493.78							
	41758.24							
NHA AGO1	42301.18	35108.89	4121.401					
	28025.48							
	35000							
AGO1	49071.62	52491	1818.996					
	55276.38							
	53125							

FIG_7_C							
	Normalised Band Density						
	Rep-1	Rep-2	Rep-3	Average	SEM	P-value:	
ago1	1	1	1	1	0		
n ha ago1	0.482573	0.406421371	0.215145	0.368	0.0795	0.001	
ago1x	1	1	1	1	0		
n ha ago1x	1.396185	1.259866871	1.853083	1.503	0.1794	Non-significant	

FIG_7_D				
	Rluc/Fluc	Average	SEM	P-value:
N-HA-Ago2	1278.195489	1424.241	92.6934	<0.0001
	1596.153846			
	1398.373984			
N-HA-Ago2 ISR Ago1	3046.728972	3302.946	140.6308	0.0004
	3531.531532			
	3330.578512			
HA-Ago2	3320.38835	3433.412	57.6039	
	3509.259259			
	3470.588235			
