# Supplementary material for: Let‐7a‐regulated translational readthrough of mammalian AGO1 generates a microRNA pathway inhibitor
Source: EMBO J. 2019 Jul 22;38(16):e100727. doi: 10.15252/embj.2018100727 (PMC6694283; doi:10.15252/embj.2018100727)
Supplement: Supplementary file 14 — Source Data for Figure 8 [file EMBJ-38-e100727-s012.pdf]

Fig_8A

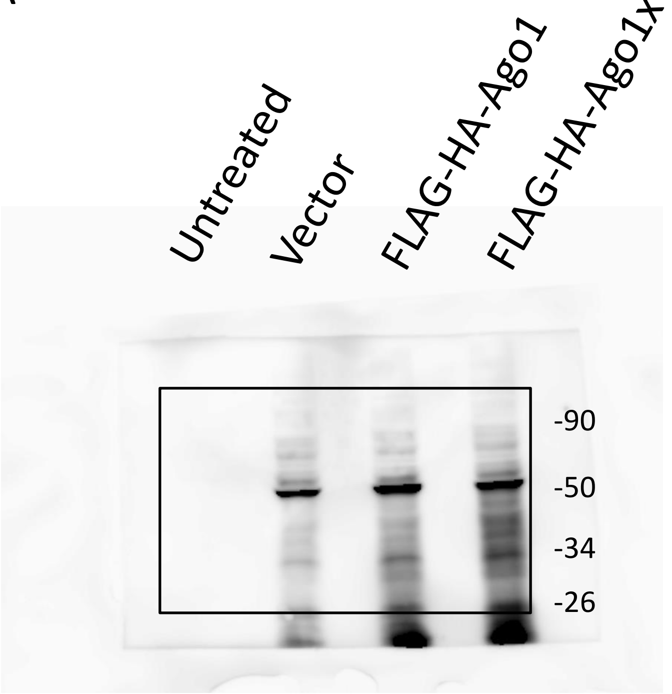

Anti- Puromycin

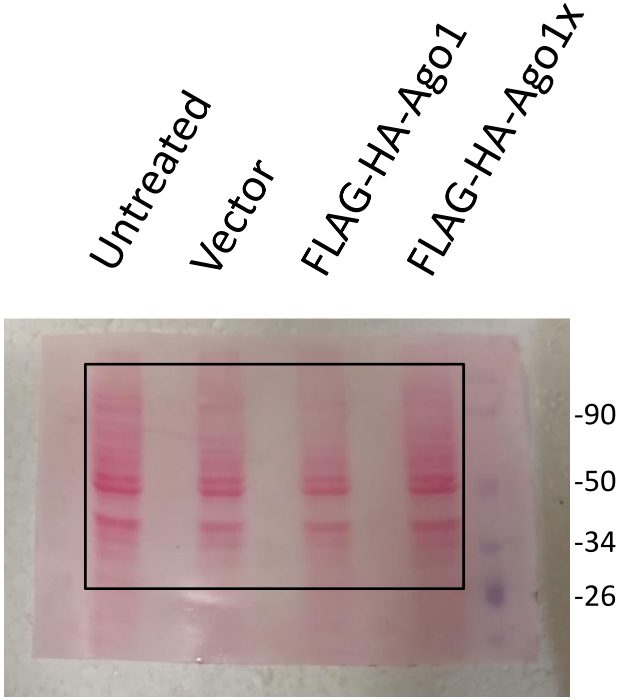

Ponceau S

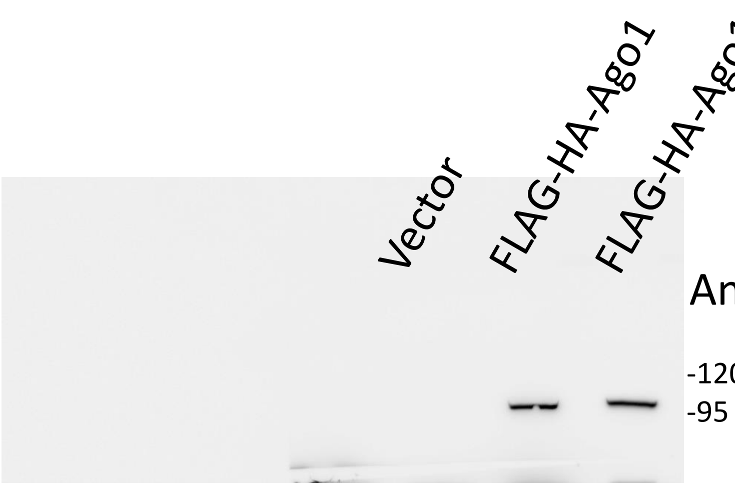

Fig_8B

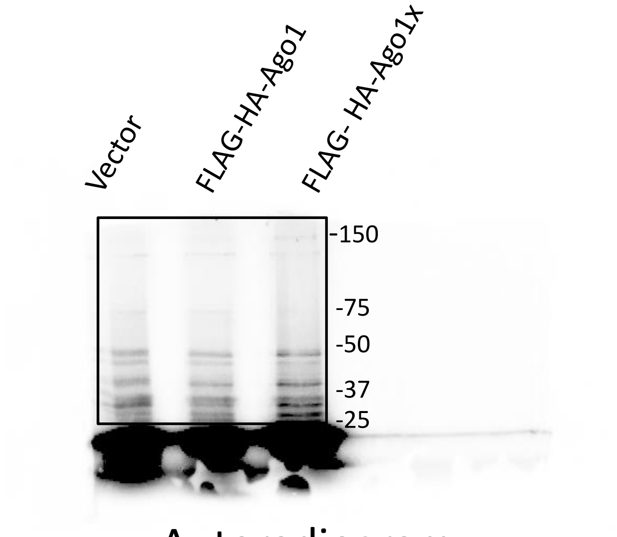

Autoradiogram

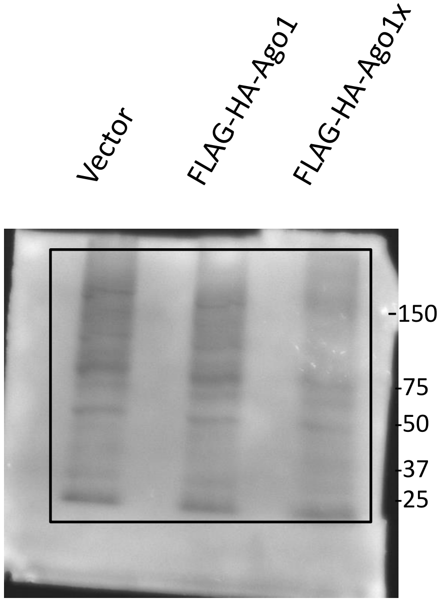

Coomassie

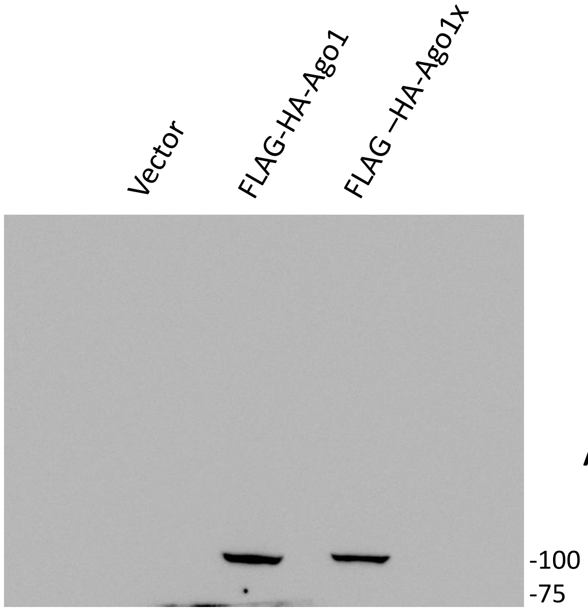

Anti-HA

FIG_8_A							
	Rep-1	Rep-2	Rep-3	Rep-4	Average	SEM	
Ago1x	2.34569898	4.00685303	4.00685303	3.748284168	3.5269	0.3984	
Ago1	1.77201277	1.73197507	1.73197507	0.755511267	1.4979	0.2476	
vector	1	1	1	1	1	0	
	P-value:	0.029	Mann-Whitney test				

**FIG_8_B**						
	**Rep-1**	**Rep-2**	**Rep-3**	**Rep-4**	**Average**	**SEM**
**Vector**	1	1	1	1	1	0
**AGO1**	1.17	1.1642	0.68	1.1611	1.0438	0.1213
**AGO1x**	2.6	2.4066	2.48	2.4266	2.4783	0.0434
			**P-value**	0.029	Mann-Whitney test	
